# A molecular basis for stoichiometric enzyme encapsulation in the vitamin B2 biosynthesis compartment

**DOI:** 10.1038/s41467-026-73260-4

**Published:** 2026-05-16

**Authors:** Lukasz Koziej, Jedrzej Pankowski, Monika Stefanska, Daniel Jankowski, Agnieszka Gawin, V. Vishal Malolan, Juha T. Huiskonen, Takahiro Kosugi, Yusuke Azuma

**Affiliations:** 1 Malopolska Centre of Biotechnology, Jagiellonian University, Krakow, Poland; 2 Faculty of Biochemistry, Biophysics, and Biotechnology, Jagiellonian University, Krakow, Poland; 3 Doctoral School of Exact and Natural Sciences, Jagiellonian University, Krakow, Poland; 4 Institute of Biotechnology, Helsinki Institute of Life Science HiLIFE, University of Helsinki, Helsinki, Finland; 5 Research Center of Integrative Molecular Systems (CIMoS), Institute for Molecular Science (IMS), National Institutes of Natural Sciences (NINS), Okazaki, Aichi Japan; 6 Molecular Science Program, SOKENDAI (The Graduate University for Advanced Studies), Hayama, Kanagawa Japan; 7 PRESTO, Japan Science and Technology Agency, Kawaguchi, Saitama Japan; 8 Present Address: Faculty of Pharmacy, Institute of Medical, Pharmaceutical and Health Sciences, Kanazawa University, Kanazawa, Ishikawa Japan

**Keywords:** Cryoelectron microscopy, Multienzyme complexes, Computational biophysics

## Abstract

Encapsulating metabolic enzymes within protein cages enhances catalytic efficiency through substrate channeling. The vitamin B2 biosynthesis system, in which a dodecahedral lumazine synthase (LS) cage encapsulates a homotrimeric riboflavin synthase (RS), exemplifies this strategy, yet the molecular basis for this stoichiometric enzyme encapsulation has remained elusive. Here, cryogenic electron microscopy structures reveal a hierarchical assembly mechanism that ensures the defined host-guest ratio. RS C-terminal cage-localization signal peptides anchor at LS pentamer-pentamer interfaces early during assembly, stabilizing open intermediates that, together with delayed later-stage cage closure, extend the loading window until guest incorporation is complete. RS spatial occupancy avoids overloading, while a molecular lock upon final closure prevents disassembly. The elucidated anchoring mechanism enabled structure-based phylogenetic analysis across diverse organisms, suggesting multiple independent evolutionary origins of this modular encapsulation strategy. This naturally occurring architecture provides design principles for engineering synthetic catalytic compartments with programmable stoichiometric control.

## Abstract (web summary)

Many bacteria sequester enzymes within protein cages for efficient vitamin B2 production. Cryo-EM captures rare cage assembly intermediates, revealing multilayered mechanisms behind architectural precision.

## Introduction

Enzyme sequestration within proteinaceous compartments is a prevalent cellular strategy for optimizing metabolic and biosynthetic reactions^1–5^. By controlling molecular flux across physical barriers, these shells establish high local concentrations of catalysts and intermediates, enhancing pathway efficiency and minimizing undesired side reactions. A prominent example is the cyanobacterial organelle-like compartment, the carboxysome, which comprises a protein shell that encapsulates ribulose 1,5-bisphosphate carboxylase oxygenase (RuBisCO) and carbonic anhydrase (CA)^6,7^. It has been hypothesized that selective bicarbonate uptake and its conversion to carbon dioxide by CA within the lumen overcomes the slow turnover rate and poor substrate specificity of RuBisCO^8,9^. In such systems, appropriate proportions of the constituent shell-forming proteins and guest enzymes are crucial for their expected morphology and reaction throughput^10–13^. Elucidating the molecular mechanisms governing the formation of those enzymatic compartments provides a foundation for prospective catalysis development in synthetic biology and metabolic engineering^14–16^.

**Fig. 1:**
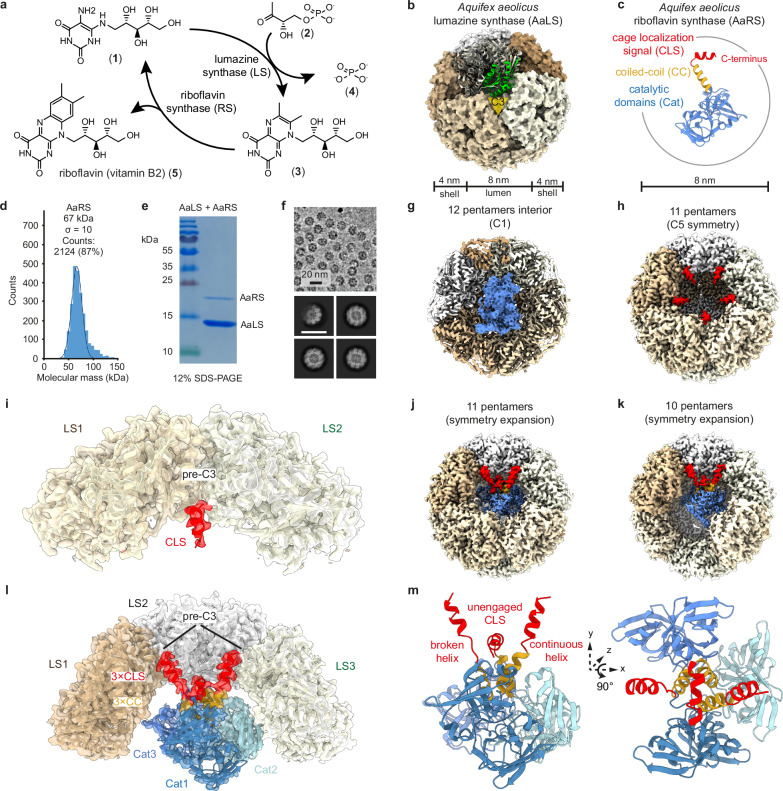
Structure of AaRS/AaLS inclusion complex. **a** Final steps of vitamin B2 biosynthesis. Lumazine synthase (LS) condenses 5-amino-6-(_D_-ribitylamino)uracil (**1**) with L-3,4-dihydroxy-2-butanone 4-phosphate (**2**) to produce 6,7-dimethyl-8-ribityllumazine (**3**) and phosphate (**4**); riboflavin synthase (RS) then converts two lumazine (**3**) molecules to one riboflavin (**5**), regenerating one uracil intermediate (**1**). **b** Cryo-EM structure (PDB ID: 9RYN) of the *Aquifex aeolicus* LS (AaLS) dodecahedral cage composed of 12 identical pentamers shown in distinct white-brown shades. One pentamer is displayed as ribbon with a single protomer highlighted in green, while the remaining pentamers are shown in surface representation. Yellow triangle (C3) indicates the threefold symmetry region of the assembly. **c** AlphaFold–predicted structure of AaRS monomer shown in cartoon representation with the cage-localization signal (CLS, red), coiled-coil (CC, orange), and tandem catalytic domains (Cat, blue). The circle indicates the cage-lumen diameter for size reference. **d** Mass photometry of AaRS showing an apparent molecular mass of 67 kDa, consistent with its homotrimer (69 kDa). **e** SDS-PAGE showing AaRS (23 kDa) co-purified with AaLS (18 kDa). Data are representative of at least three independent experiments with similar results. Source data are provided as a Source Data file. **f** Cryo-EM micrograph (top) and representative 2D class averages (bottom) of AaLS/AaRS inclusion complex. Data are representative of two independent experiments using different protein batches; see Supplementary Fig. 1 for additional 2D classes. **g** 3D reconstruction of the inclusion complex without imposed symmetry (C1). The cross-section shows lumenal density (blue), though poorly defined, corresponding to encapsulated AaRS. **h** C5-symmetrized cryo-EM reconstruction of the AaLS 11-pentamer assembly. Extra density (red) at the pentamer-pentamer interaction interface (pre-C3 region) is identified as AaRS-CLS. **i** Enlarged view of two neighboring AaLS pentamers (LS1 and LS2) bound to an AaRS-CLS (red) at the pre-C3 region, shown as cryo-EM density and the fitted molecular model (PDB ID: 9RYJ). **j**, **k** Symmetry-expanded 3D reconstructions of 11- (**j**) and 10-pentamer (**k**) AaLS inclusion complexes with an AaRS trimer. **l** Enlarged view of three neighboring AaLS pentamers (LS1–LS3) and the bound AaRS homotrimer from the symmetry-expanded 11-pentamer cage, shown as cryo-EM density and the fitted model (PDB ID: 9RYK). AaRS domains are colored as in (**c**). The presented cryo-EM density was post-processed using DeepEMhancer^76^ for clear visibility of the AaRS region, while the unsharpened map, used for molecular model fitting, is provided in Supplementary Fig. 5. **m** The corresponding molecular model of the AaRS homotrimer in two orientations, showing individual domain architecture and relative positioning.

The riboflavin synthase (RS)/lumazine synthase (LS) complex is a proteinaceous compartment for riboflavin (vitamin B2) biosynthesis found in certain bacteria^3^. A dodecahedral cage formed by LS^17^, which catalyzes the penultimate step in riboflavin biosynthesis, encapsulates a homotrimeric RS, the subsequent enzyme in the pathway (Fig. 1a–c). This spatial organization has been shown to enhance the overall riboflavin formation rate when the substrate concentrations are low^18^. Unlike the numerous distinct protein components required for the formation of carboxysomes and other bacterial microcompartments (BMCs)^2,19,20^, this vitamin B2 biosynthetic system comprises only two types of enzymes and is readily reconstituted recombinantly^21^, making it a practical model for investigating the molecular basis of precise inclusion complex formation with defined morphology and constituent stoichiometry underlying efficient biocatalysis.

The C-terminal segment of RS acts as a cage-localization signal (CLS) that directs its encapsulation by LS cages. Fusion of RS-derived CLS to a non-associated protein, i.e., green fluorescent protein (GFP), and its co-production with LS in *Escherichia coli* results in inclusion complex formation, as demonstrated with variants from *Aquifex aeolicus*^22^ and *Bacillus subtilis*^23^. The CLS peptides have been hypothesized to form an amphipathic α-helix and to recognize the LS lumenal surface at shell protein junctions through hydrophobic interactions^22^, based on analogy with the homotrimeric dye-decolorizing peroxidase (Dyp) enclosed by encapsulin shell^24^. Such junction recognition has also been observed in carboxysomes^25^ and propanediol utilization (Pdu) compartments^26^. However, the actual binding mode of the RS-CLS to LS has remained elusive due to the absence of structural evidence. X-ray crystallography has been hindered by the non-aligned orientation of the guest RS in crystals formed by highly symmetrical LS cages^27^.

Here, we report cryogenic electron microscopy (cryo-EM) structures of *Aquifex aeolicus* riboflavin synthase/lumazine synthase (AaRS/AaLS) complex intermediates that reveal the CLS interaction mode and the assembly mechanism enforcing the precise 3:60 (RS:LS) stoichiometry of the vitamin B2 biosynthesis compartment. The elucidated structure enabled prediction of RS/LS complex formation across organisms, suggesting the evolutionary distribution and modularity of this encapsulation strategy. The regulatory assembly mechanism demonstrated by bacteria provides design principles for constructing synthetic catalytic compartments with defined stoichiometry.

## Results

### AaRS anchors at the AaLS pentamer-pentamer interface

We first characterized the individual quaternary structures of AaLS and AaRS as the starting point for investigating their interaction. Both proteins were heterologously produced in *E. coli* and purified for structural characterization. AaLS is known to form a dodecahedral structure comprising 60 protomers, which was also confirmed in this study by cryo-EM single-particle reconstruction (Fig. 1b). AaRS comprises tandem catalytic sites, a coiled-coil domain, and CLS (Fig. 1c) and was predicted to form a homotrimer, analogous to the crystal structure of *E. coli* ortholog, where the coiled-coil domain mediates trimerization^28^. Mass photometry analysis of purified AaRS validated this prediction, revealing an apparent molecular mass of 67 kDa, closely aligning with the theoretical mass of a homotrimer (69 kDa) (Fig. 1d). These results confirm that recombinant AaLS and AaRS assemble inherently into dodecahedrons and homotrimers, respectively.

To characterize the AaRS/AaLS complex, AaRS and Strep-tagged AaLS were coexpressed in *E. coli* and isolated using affinity and size-exclusion chromatography. SDS-PAGE analysis confirmed the copurification (Fig. 1e), and cryo-EM revealed particles with the expected diameter of ~16 nm (Fig. 1f). Three-dimensional reconstruction without imposed symmetry (C1) yielded substantial density inside the dodecahedral structure, indicating successful encapsulation of AaRS (Fig. 1g). However, the guest cryo-EM density was weak and poorly defined, particularly at the anticipated interface between AaRS and AaLS, precluding atomic-level insight into their interaction. Despite testing chemical crosslinking and various reconstruction strategies, including local refinement, symmetry expansion, and subtomogram averaging, no significant improvement was achieved. The persistent weak guest density suggests conformational heterogeneity of AaRS within the AaLS cages rather than binding to a specific lumenal site.

Upon analyzing the cryo-EM data of the AaRS/AaLS complex by 3D classification, we realized that a substantial population of particles consisted of incomplete AaLS dodecahedra (Supplementary Figs. 1 and 2). These structures lacked one to three pentameric units. In contrast, when AaLS was produced alone under identical conditions, incomplete assemblies were less frequent. Specifically, only 42% of particles were classified as complete 12-pentamer assemblies when AaRS was present (Supplementary Fig. 1a), compared to 80% in the absence of guest cargo (Supplementary Fig. 3a). These findings indicate that coproduction with AaRS hinders AaLS cage completion.

The presence of incomplete AaLS assemblies prompted us to investigate whether AaRS might interact where pentameric building blocks are missing. As this region features a local fivefold symmetry, we performed a 3D reconstruction of the 11-pentamer particles by applying C5 symmetry. This approach revealed clearly defined additional cryo-EM density attributed to parts of AaRS (Fig. 1h, red, and Supplementary Fig. 1b). Notably, this extra density was localized not to the lumenal surface but to the AaLS pentamer-pentamer interaction interface, the region forming threefold symmetry upon docking of another pentamer, referred to hereinafter as the “pre-C3” area (Fig. 1i). This cargo recognition mechanism, targeting the actively assembling interface rather than the lumenal surface, has never been observed in any other proteinaceous compartment.

The homotrimeric AaRS presents three CLS peptides, while the pentagonal pore provides five potential pre-C3 binding sites, indicating that not all binding sites would be equivalently occupied. To resolve the AaRS arrangement, we applied symmetry expansion to the 11-pentamer particles. The resulting density map shows two CLS peptides from the AaRS trimer occupying adjacent corners of the pentagonal pore, while the third CLS lies between them without contacting the AaLS surface (Fig. 1j and Supplementary Fig. 1b). The same configuration was observed in the 10-pentamer assembly (Fig. 1k and Supplementary Fig. 1c). The identical binding mode in both complexes suggests that AaRS recruitment occurs at early assembly stages, with the captured 10- and 11-pentamer particles representing relatively abundant late intermediates of cage formation.

Size comparison of the pentagonal pore and AaRS trimer reveals that the guest cargo requires at least three pentamers missing from the complete cage to pass through the opening (Supplementary Fig. 4a, b). This suggests that the majority of AaRS recruitment occurs between early and intermediate assembly stages, consistent with the observation of AaRS trimer bound to both 10- and 11-pentamer assemblies. Meanwhile, if AaRS fail to trimerize beforehand, individual monomers can fit through smaller pores and may enter to trimerize within the cage (Supplementary Fig. 4c), possibly providing an additional layer of stoichiometry control.

The refined structure revealed the role of the AaRS coiled-coil domain as a primary organizing unit (Fig. 1l, m). It spans and directs the two CLS peptides toward adjacent pre-C3 binding sites, and the resulting multivalency may enhance overall binding affinity. To achieve this arrangement, the two CLS peptides adopt different secondary structure conformations at their junctions with the coiled-coil. One CLS maintains a nearly continuous α-helix with the coiled-coil, while the linker to the second CLS contains a break for positional adjustment (Fig. 1m). This architectural asymmetry, particularly the non-helical linker, would modulate the overall binding affinity, making it sufficient for anchoring without completely blocking the final pentamer docking during cage completion.

Beyond CLS positioning, the coiled-coil domain constrains the spatial arrangement of the three catalytic domains within the lumenal space. The cryo-EM density for catalytic domains was less well-defined compared to the CLS regions (Supplementary Fig. 2), and no substantial contacts with the AaLS shell were identified. While the coiled-coil exhibits threefold symmetry, the catalytic domains adopt non-symmetric arrangements (Fig. 1m), indicating flexibility in their interdomain connections. These observations collectively indicate that the catalytic domains remain tethered to the anchored structure primarily through the coiled-coil and CLS peptides. Despite this flexibility, the complete trimeric AaRS model could be confidently fitted into the available density, demonstrating that the space at the anchoring site accommodates a single homotrimer (Fig. 1l and Supplementary Fig. 5). Notably, this occupancy leaves minimal excess volume insufficient for another trimeric AaRS. This spatial organization, imposed by the coiled-coil domain, likely prevents guest overloading during the assembly.

### Interaction mode of the AaRS-CLS

**Fig. 2:**
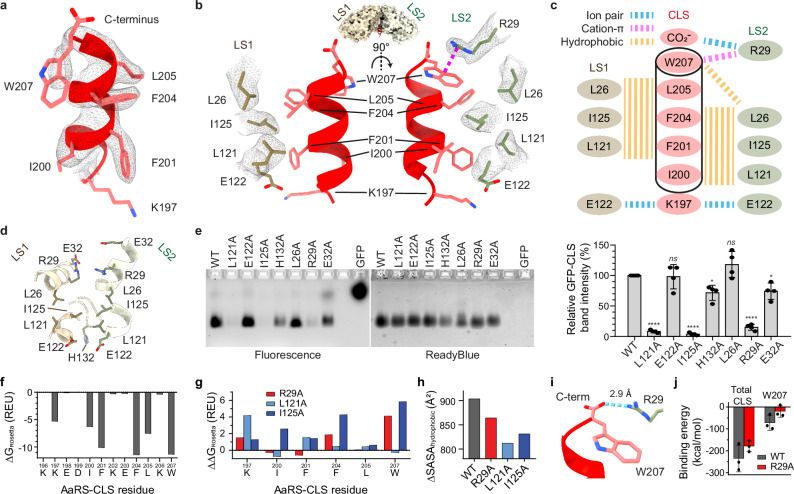
Structural basis and energetics of AaRS-CLS binding to AaLS. **a** The structure of AaRS cage-localization signal (CLS) shown in cartoon representation with key side chains as sticks (PDB ID: 9RYJ). Cryo-EM density (mesh) from the C5-symmetrized AaLS/AaRS inclusion complex reconstruction is contoured around the CLS. **b** Binding mode of CLS at the interface between two LS pentamers labelled as LS1 (left, brown) and LS2 (right, green). The R29–W207 cation–π interaction is highlighted in magenta. **c** Contact map derived from the cryo-EM and molecular dynamics (MD). CLS and LS are colored as in (**b**). Dashed lines connect interacting residues, color-coded by bond type (blue, ion pair; magenta, cation–π; orange, hydrophobic). **d** LS residues at the pre-C3 region subjected to alanine-scanning mutagenesis (PDB ID: 9RYO). **e** Native agarose gel electrophoresis for pull-downs of AaLS wild-type (WT) and alanine mutants co-produced with GFP-CLS. The same gel was visualized by fluorescence imaging (left) and ReadyBlue staining (right). Free GFP-CLS is present at concentrations below the detection limit of ReadyBlue staining under these loading conditions and is visualized only by fluorescence. Bar graph shows relative GFP fluorescence normalized to the AaLS band intensity, with the values for WT set to 100%. Data represent means ± standard deviations from biological quadruplicates with individual data points overlaid (one-way ANOVA: *F*(7, 24) = 54.54, *p* < 0.0001; followed by Dunnett’s test: *ns*, *p* ≥ 0.05; *, *p* < 0.05; ****, *p* < 0.0001). Adjusted *p* values and 95% confidence intervals (Cl) for mean differences relative to WT are as follows: L121A (*p* < 0.0001, 95% CI [67.1, 116.3]), E122A (*ns*, *p* = 0.9997, 95% CI [−22.8, 26.5]), I125A (*p* < 0.0001, 95% CI [71.4, 120.7]), H132A (*p* = 0.0191, 95% CI [3.8, 53.0]), L26A (*ns*, *p* = 0.2173, 95% CI [−42.7, 6.5]), R29A (*, *p* < 0.0001, 95% CI [60.3, 109.6]), and E32A (*p* = 0.0369, 95% CI [1.2, 50.4]). **f** Binding energy contribution (ΔG_rosetta_, in Rosetta Energy Units, REU) for each AaRS-CLS residue upon binding to the AaLS pre-C3 interface, calculated using Rosetta InterfaceAnalyzer. Negative values indicate a favorable contribution to binding stability. **g** Effect of AaLS pre-C3 mutations (R29A red; L121A light blue; I125A dark blue) on binding of CLS, presented as ΔΔG_Rosetta_ = ΔG_Rosetta,WT_ – ΔG_Rosetta,mut_ (REU) for CLS residues with favorable contributions in (**f**). Positive values indicate destabilizing mutations that reduce binding affinity. Per-residue ΔG_Rosetta_ values for all CLS residues are shown in Supplementary Fig. 8a. **h** Buried hydrophobic solvent-accessible surface area (ΔSASA_hydrophobic_) of CLS at the AaLS pre-C3 interface for wild-type (gray) and mutants (colors as in **g**), computed with Rosetta. **i** MD–derived model showing R29 forming an ion-pair interaction with the CLS C-terminus as an alternative to the cation–π contact with W207. See also Supplementary Movie 1, showing this simulation over 1 μs (100 frames). **j** MD-derived binding energies of interaction between CLS and pre-C3 for AaLS WT (gray) or R29A (red); left, total CLS; right, W207 residue contribution. Per-residue binding energy values for all CLS residues are shown in Supplementary Fig. 8b. Data present means ± standard deviations from triplicate simulations with individual data points overlaid. Source data for (**e**–**h** and **j**) are provided as a Source Data file.

Close examination of the cryo-EM structure reveals how the AaRS-CLS binds with the AaLS pentamer interface (Fig. 2a–c). The AaRS-CLS adopts an amphipathic α-helix displaying the side chains of the amino acid residues, I200, F201, F204, L205, and W207 (Fig. 2a), toward the pre-C3 hydrophobic surface comprising L26, L121, and I125 from two AaLS protomers (Fig. 2b). E122 of AaLS caps the hydrophobic cluster through the aliphatic moiety at the interior side of the cage assembly. At the exterior side, an R29 residue of AaLS binds with W207 of the AaRS-CLS through cationic-π interaction. This R29 residue is also near the carboxyl terminus of AaRS, and E122 is close to K197 of the CLS, possibly forming salt bridges. However, these electrostatic interactions are likely dynamic. Cryo-EM density could not determine a specific rotamer for the charged moiety of these amino acid side chains.

Based on the observed interaction mode, we introduced single alanine mutations into AaLS at different positions and tested their ability to form a complex with AaRS-CLS fused to GFP, GFP-CLS^22^. In addition to L26, R29, L121, E122, and I125, we also examined another residue located near the threefold symmetry region, E32, and one facing the interior, H132 (Fig. 2d). These AaLS mutants possessing StrepII-tag were coproduced with GFP-CLS in *E. coli* cells, isolated using Strep-Tactin affinity chromatography, and subsequently analyzed by native agarose gel electrophoresis (AGE) (Fig. 2e). Significant decreases in the complex formation efficiency with the GFP-CLS were observed for the AaLS variants containing R29A, L121A, or I125A mutations, with 92, 96, or 85% drops compared to that for wild-type AaLS (WT), respectively. In contrast, the other mutations (E122A, H132A, L26A, and E32A) showed minor effects on the guest encapsulation, retaining 98, 72, 118, and 74% of wild-type complex formation efficiency, respectively. L26A retains full CLS binding despite its position in the hydrophobic packing region, which may reflect its structural role in supporting neighboring residues, particularly I125, rather than forming extensive direct contacts with CLS. While AaLS is known to adopt a variety of assembly geometries upon mutagenesis^29–33^, such a morphological alteration is not the reason for the decreased encapsulation efficiency. Cryo-EM structures of the AaLS R29A, L121A, and I125A variants without cargo all show the wild-type-like dodecahedral and 11-pentamer assembly without significant changes in protein folding (Supplementary Figs. 3, 6, and 7). These results establish R29, L121, and I125 of AaLS as the essential residues in the CLS-mediated encapsulation.

To quantify the contributions of individual AaRS-CLS residues to the interaction with AaLS, we performed computational simulations using Rosetta InterfaceAnalyzer (Fig. 2f–h and Supplementary Fig. 8a). These confirmed that hydrophobic residues of the CLS contribute substantially to interface stability (Fig. 2f). The L121A and I125A mutations resulted in significant energy losses (Fig. 2g, light and dark blue bars), accompanied by marked decreases in the delta hydrophobic solvent-accessible surface area (ΔSASA) at the binding interface (Fig. 2h). The R29A mutation resulted in significant loss in the local binding energy contribution of W207 as well as reduced ΔSASA (Fig. 2g, h, red bars), indicating that R29 is important for maintaining the complementary hydrophobic surface likely through its cationic-π interaction with W207.

Molecular dynamics (MD) simulations revealed that R29 of AaLS dynamically forms electrostatic interactions with the C-terminus of AaRS-CLS in addition to the cationic-π interactions with W207. Snapshots captured the guanidinium head within 2.9 Å of the terminal carboxyl group (Fig. 2i and Supplementary Movie 1), which was unresolved in the cryo-EM structure (Fig. 2a, b). This essential role was validated by the R29A mutation, which resulted in a dramatic energy loss in the MD simulations (Fig. 2j and Supplementary Fig. 8b) and a 92% decrease in complex formation efficiency in the pull-down assays with GFP-CLS (Fig. 2e). While MD simulations suggested contributions from interactions between K196 and K197 of CLS with E122 of AaLS (Supplementary Fig. 8c and Supplementary Movie 1), the E122A mutation showed no significant impact in the pull-down assay (Fig. 2e), indicating a supplementary role for binding. Together, these results establish R29 as the key anchor for CLS recognition through its dual cationic-π and electrostatic interactions.

### Molecular gate and lock for the final pentamer docking into AaLS assembly

**Fig. 3:**
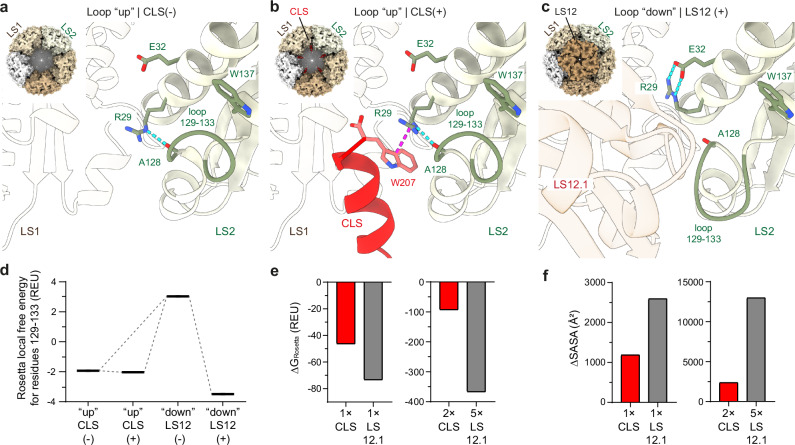
Gate-and-lock mechanism for final pentamer docking in AaLS cage. **a** Pentagonal pore in empty 11-pentamer AaLS (PDB ID: 9RYO). The loop spanning residues 129–133 adopts a conformation, “up” state, that occludes W137. The backbone carbonyl oxygen of A128 forms a hydrogen bond (cyan dashed line) with the guanidinium group of R29, orienting it toward the CLS binding site. **b** Same view of 11-pentamer AaLS assembly bound with CLS (red, PDB ID: 9RYJ). The loop state persists as CLS engages the pre-C3 site. The R29–W207 cation–π interaction is shown (magenta dashed line). **c** Fully assembled dodecahedral cage (12 pentamers, PDB ID: 9RYI). Final pentamer docking induces the loop transition to the “down” state, exposing W137 and redirecting R29 to E32 to form an electrostatic lock (cyan dashed lines) that sequesters R29 from the CLS-binding configuration. The protomer interacting with the W137 is not displayed for clear visualization of the loop region. Panels use cartoon representation with selected side chains in sticks, with oxygen and nitrogen atoms colored red and blue, respectively. The top-left insets show the overall view of the corresponding assembly. Pentamers are labeled as LS1, LS2, and LS12 to link overall and amplified views. **d** Rosetta local free energy for the loop region (residues 129–133), given in Rosetta Energy Units (REU). Negative values indicate stable conformation. Calculations were performed on AaLS protomers from the 11-pentamer assemblies with and without CLS (“up” CLS (+) and (−)), and from fully assembled dodecahedron (“down” LS12 (+)), where LS12 represents the final pentamer docked in the assembly. The energy for “down” LS12 (−) was estimated by removing this pentamer unit in silico. **e**, **f** Binding energy (**e**, ΔG_Rosetta_) and buried solvent-accessible surface area (**f**, ΔSASA) for two AaLS adjacent pentamers (LS1 and LS2) binding to either one CLS or one AaLS protomer (LS12.1). Calculation was performed per binding site (left graphs), and scaled by the number of actual bindings, 2× for CLS or 5× for final pentamer insertion (right graphs). Negative values for ΔG_Rosetta_ and positive values for ΔSASA indicate a favorable contribution to binding stability and large contact surfaces, respectively. Source data for (**d**–**f**) are provided as a Source Data file.

Comparison of partially and fully closed dodecahedral AaLS structures revealed a potential molecular mechanism for the kinetic accumulation of late assembly intermediates. In any incomplete AaLS assembly, including 11-pentamer structures and regardless of guest AaRS presence, the loop comprising residues 129–133 adopts an “up” conformation that occludes W137 (Fig. 3a, b). Upon the final pentamer docking, the loop transitions to a “down” state, exposing W137 for hydrophobic interaction with the incoming partner (Fig. 3c). Rosetta free energy calculation reveals that the “up” conformation is inherently preferred mainly due to W137 interactions with the loop region, where CLS-binding at the pre-C3 site provides minor additional stabilization (Fig. 3d). These results suggest each AaLS pentamer-pentamer interaction must overcome this barrier. At the pentagonal gap of the 11-pentamer assembly, five copies of 129–133 loop are positioned directly in the insertion pathway, and the incoming final pentamer harbors five additional loops. The final docking step would, therefore, require coordinated shifts of ten loops, presumably driven by spontaneous fluctuations and/or mechanical displacement. While these loops can rearrange through induced fit during earlier lateral assembly, the approach angles available for incoming pentamers become increasingly restricted as assembly advances. This restricted geometry likely increases the difficulty of overcoming the loop-mediated barrier, resulting in a progressive slowdown in assembly. This anti-cooperative behavior, reversing the typical acceleration in self-assembly^16,34,35^, explains an inherent kinetic barrier in AaLS that delays final pentamer docking even without CLS-mediated anchoring.

While the kinetic gating mechanism and CLS-mediated blocking may facilitate guest protein loading by maintaining a transiently open state during assembly, the porous configuration is ultimately unfavorable for the catalytic function of the compartment. The large gap in 11-pentamer assembly prevents sequestration of reaction intermediates (Fig. 1a), thereby precluding the expected enhancement of riboflavin biosynthesis. Moreover, even after the final pentamer docks to complete the dodecahedron, CLS peptides could continue to target the same pre-C3 binding sites from the inside, posing the risk of pentamer displacement and cage reopening. A mechanism to stabilize the completed structure is therefore essential to maintain substrate channeling within the biosynthetic pathway for efficient vitamin production.

Thermodynamic preference serves as a basis for preventing competing interactions that could arise from CLS rebinding and AaLS pentamer displacement. Rosetta InterfaceAnalyzer calculations show that AaLS protomer binding to two adjacent pentamers is favored over CLS anchoring, with approximately 1.6- and twofold difference in binding energy and ΔSASA, respectively (Fig. 3e, f, left graphs). The final pentamer docking also locally stabilizes the otherwise unfavored “down” loop conformation (Fig. 3d). The energetic difference becomes even more pronounced when considering that five protomers are involved in the final pentamer insertion, versus only two for CLS binding (Fig. 3e, f, right graphs). Despite this thermodynamic preference, CLS appears to remain anchored at pre-C3 sites during assembly, possibly because incoming pentamers may preferentially dock to available CLS-free sites. Such binding site selectivity would delay engagement at specifically CLS-occupied sites until cage completion becomes necessary.

Beyond the thermodynamic preference, the loop conformational change upon final pentamer docking provides a mechanistic solution to prevent the unwanted AaLS cage disassembly. In the “up” state observed in the 11-pentamer structure, carbonyl oxygen atom of A128 in the loop interacts via hydrogen bond with R29, the essential residue for CLS peptide recognition, and orients it to the corresponding binding site at the pre-C3 site (Fig. 3a, b). Upon final pentamer docking and loop repositioning to the “down” state, R29 flips to form a new electrostatic interaction with E32 (Fig. 3b). We propose that this R29-E32 interaction acts as a molecular lock that sequesters R29 away from the CLS-binding configuration, thereby preventing both CLS peptide rebinding and pentamer dissociation. Supporting this model, the E32A mutant, which disrupts the R29-E32 interaction, exhibited a substantial band corresponding to unbound GFP-CLS in pulldown experiments (Fig. 2e), suggesting that guest proteins had escaped from cage assemblies after isolation. Together, the conformational gate and molecular lock provide temporal control over AaLS assembly, with the gate extending the window for guest loading and the lock ensuring irreversible cage completion.

### RS-LS complex formation across taxa

**Fig. 4:**
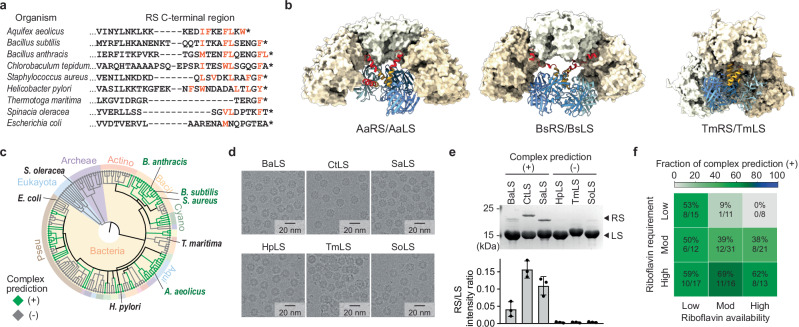
Taxonomic distribution and lifestyle correlation of RS/LS complex formation. **a** Amino acid sequence alignment of RS C-termini from selected organisms. Bulky hydrophobic residues are highlighted in orange. Asterisks indicate sequence termini. **b** Representative AlphaFold predictions of RS/LS complex formation with the variants from *Aquifex aeolicus* (AaRS/AaLS), *Bacillus subtilis* (BsRS/BsLS), and *Thermotoga maritima* (TmRS/TmLS). Three LS pentamers (15 chains total) are shown as surfaces in white-brown shades; RS homotrimer (3 chains) shown as ribbons with domains colored distinctly: catalytic domain (blue), coiled-coil (orange), and CLS (red). All the models predicted for these 3 variants and enlarged views of a CLS-pre-C3 interaction are provided in Supplementary Figs. 9 and 10, respectively. **c** Taxonomic distribution of 166 analyzed organisms displayed as a dendritic tree. Complex-forming organisms (green, +) were classified based on AlphaFold predictions showing CLS binding to pentamer-pentamer interface at the pre-C3 region in at least one out of 15 models (see the details in Methods), and others are shown in gray (−). Bacterial phyla are indicated by colored bands, with major phyla labeled: Actino Actinomycetota, Bacil Bacillota, Cyano Cyanobacteriota, Aqui Aquificota, Pseu Pseudomonadota. **d**, **e** Experimental validation of predicted complex formation: representative cryo-EM micrographs (**d**) and SDS-PAGE analysis (**e**) of LS co-produced with cognate RS in *E. coli* cells. The bar graph shows RS/LS band intensity ratios. Data are presented as means ± standard deviations with individual data points from three biological replicates. Source data are provided as a Source Data file. **f** Correlation between complex formation and lifestyle. Heat map showing the percentage of organisms with predicted RS/LS complex formation as a function of riboflavin requirement and its environmental availability. Numbers in each box indicate the percentage (top) and the ratio of complex-positive organisms to total organisms in that category (bottom).

*A. aeolicus* and *B. subtilis* utilize CLS-mediated encapsulation of RS within LS cages, yet this enzyme compartmentalization is not universal across organisms. LSs from fungi, archaea, and certain eubacteria are known not to form dodecahedra, but exist as pentamers or dimers of pentamers^36–41^. Furthermore, cage-forming LS does not universally encapsulate RS, as exemplified by *E. coli* LS (EcLS), which has been isolated without associated EcRS^42^. It has been proposed that C-terminal sequences of RS potentially serve as an indicator for predicting complex formation^22^, as RS proteins from several organisms possess sequences that could form amphiphilic α-helices similar to AaRS-CLS (Fig. 4a). However, the extent and mechanism of this RS/LS association across different species have remained unknown.

To investigate the prevalence of CLS-mediated encapsulation, we employed AlphaFold-based structure prediction. Based on the observed CLS anchoring at the pre-C3 region, the interaction prediction was performed using three copies of RS and 15 copies of LS monomers as input. AlphaFold successfully recapitulated CLS binding to pre-C3 sites for RS/LS from *A. aeolicus* (AaRS/AaLS) and *B. subtilis* (BsRS/BsLS), while predicting no such binding for *Thermotoga maritima* enzymes (TmRS/TmLS), where the RS C-terminal extension peptide is shorter than the former two variants, unlikely sufficient for the canonical binding (Fig. 4b and Supplementary Figs. 9 and 10a–c). These results prompted us to extend the analysis to RS/LS pairs from 166 organisms spanning bacteria, archaea, fungi, and plants. Sixty variants, all derived from bacteria, were predicted to utilize CLS-mediated complex formation based on CLS engagement with pre-C3 sites in at least one top-ranked model (Fig. 4c and Supplementary Tables 1 and 2).

To validate these predictions experimentally, we heterologously coproduced RS and LS pairs from six organisms in *E. coli*. Following affinity purification of Strep-tagged LS, cryo-EM confirmed that all variants formed cage-like particles similar to AaLS (Fig. 4d). SDS-PAGE analysis revealed clear RS/LS copurification for three variants predicted to form CLS-mediated complexes: *Bacillus anthracis* (Ba), *Chlorobaculum tepidum* (Ct), and *Staphylococcus aureus* (Sa) (Fig. 4e and Supplementary Figs. 10d–f and 11). In contrast, three variants predicted not to utilize pre-C3 CLS anchoring showed no substantial copurification: *Helicobacter pylori* (Hp), *Thermotoga maritima* (Tm), and *Spinacia oleracea* (So) (Fig. 4e and Supplementary Fig. 12). These experimental results showed complete concordance with AlphaFold predictions, demonstrating the utility of this approach for identifying CLS-mediated RS/LS pairs.

Taxonomic classification of the 144 bacterial organisms revealed that CLS-mediated encapsulation is distributed across multiple bacterial phyla (Fig. 4c and Supplementary Fig. 13). To investigate the evolutionary history underlying this broad distribution, we performed phylogenetic analysis of RS sequences (Supplementary Fig. 14). This analysis showed that complex-forming variants form multiple distinct clusters rather than a single monophyletic group, indicating multiple independent evolutionary events to gain/loss the compartmentalization probability. For instance, complex-forming organisms include a large clade of Bacillota (e.g., *Bacillus*, *Clostridium*), a separate clade of photosynthetic Cyanobacteriota, and a distinct thermophilic cluster (i.e., Aquificota and Thermodesulfobacteriota), with these groups separated by substantial phylogenetic distance. This scattered pattern likely reflects the short, modular nature of the CLS peptide motif, which enables facile evolutionary transitions.

To explore potential ecological drivers of this evolutionary lability, we classified bacterial organisms based on their metabolic riboflavin requirements and environmental riboflavin availability (Fig. 4f and Supplementary Table 3). Among organisms with minimal flavin-dependent metabolism, including genome-reduced endosymbionts (*Buchnera aphidicola*) and obligate intracellular pathogens (*Chlamydia*, *Mycobacterium leprae*), the predicted prevalence of complex formation decreased as environmental availability increased (Fig. 4f, top panels). This pattern may reflect reduced evolutionary pressure to maintain the encapsulation system when metabolic demands are minimal and vitamin supply is abundant. However, even within ecological niches where selective pressure would be expected to be strongest, the prevalence of predicted complex formation never exceeded 69% within the tested variants (Fig. 4f, middle and bottom panels), indicating that a substantial fraction of organisms utilizes alternative strategies even when the encapsulation system might be advantageous. This observation suggests that CLS-mediated encapsulation represents one of several evolutionarily labile strategies bacteria have evolved for riboflavin metabolism, with its distribution reflecting adaptation to diverse ecological and metabolic contexts.

## Discussion

**Fig. 5:**
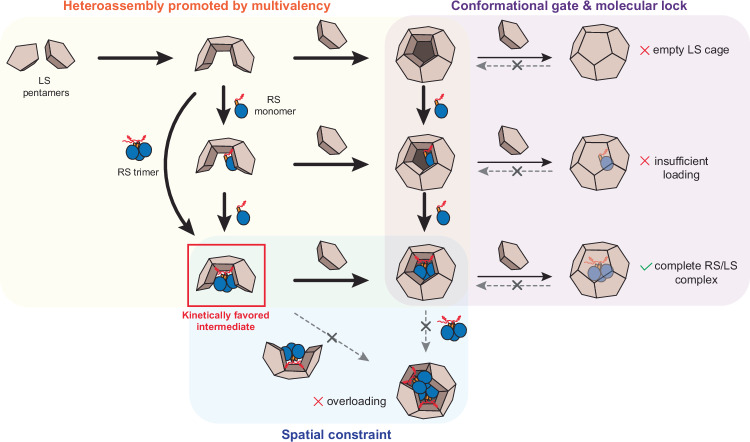
Proposed hierarchical assembly mechanism controlling RS/LS complex stoichiometry. Left panel (orange): Heteroassembly promoted by multivalency. RS trimers display three CLS peptides and early-stage LS cages present multiple pre-C3 sites, enabling multivalent binding that kinetically favors RS-trimer-bound intermediates (downward pathway) over monomer-bound forms (middle pathway) or guest-free LS cage precursors (upper pathway). The pathways illustrate representative examples of potential assembly routes, RS monomers may bind first then trimerize, or pre-formed RS trimers may bind directly, but cooperative binding can also occur through alternative routes, such as two pentamers binding to an RS trimer followed by recruitment of additional pentamers to form further pre-C3 sites. Regardless of the specific route, the multivalent interaction architecture theoretically favors heterocomplex formation (red box). Middle-bottom panel (blue): Spatial constraint. The coiled-coil arrangement of RS catalytic domains within the lumen physically prevents accommodation of more than three RS monomers, blocking overloading (gray dashed arrow indicates blocked pathway). Right panel (purple): Conformational gate and molecular lock. An open conformation of the LS loop and CLS anchoring maintains transiently open intermediates, preventing premature cage closure (thin arrows indicate slower kinetics compared to thick arrows for earlier assembly steps). Upon final pentamer docking, LS loop closure accompanies CLS release and the R29-E32 molecular lock stabilizes the closed complex, preventing disassembly (gray dashed arrows indicate irreversibility of the process). Green checkmark indicates successful complete RS/LS complex formation with defined stoichiometry; red X marks indicate suppressed off-pathway products (empty cage, insufficient loading, overloading). Note that the intermediates shown represent theoretical assembly states, rather than experimentally confirmed structures, inferred from observed binding modes and structural data, designed to illustrate how the layered mechanisms prevent unwanted species and ensure proper stoichiometric control.

Cryo-EM structures reveal a hierarchical mechanism for controlling the stoichiometry of the AaRS/AaLS inclusion complex (Fig. 5). During early assembly, the AaRS oligomer displays multiple CLS peptides via its coiled-coil domain, while nascent AaLS cages present multiple pre-C3 binding sites, enabling multivalent interactions that kinetically favor heterocomplex formation over empty cage assembly (Fig. 5, left, orange). Moreover, the CLS anchoring combined with conformational gating at the pentamer-pentamer interface maintains the cage in a transiently open state, providing sufficient time for complete guest loading and preventing premature closure that would result in under-loaded assemblies (Fig. 5, right, purple). Conversely, the coiled-coil-directed spatial arrangement of tethered catalytic domains within the lumen prevents the opposite problem of overloading by physically blocking accommodation of more than three AaRS monomers per cage (Fig. 5, bottom, blue). Upon completion of the dodecahedron, the thermodynamic preference and the R29-E32 molecular lock prevents pentamer release, ensuring sustained encapsulation required for substrate channeling and catalytic enhancement. These layered mechanisms provide molecular proof for how the defined 3:1 guest-to-cage stoichiometry is achieved, favoring proper complex formation while suppressing under/overloading and disassembly.

The CLS anchoring at the cage-building interface distinguishes the RS/LS loading mechanism from lumenal surface recognition used by other bacterial compartments^24,43^. Carboxysome and the related BMCs typically requires guest enzyme arrays organized via scaffolding proteins to achieve assemblies with defined geometry^44–47^, and disrupting this network leads to irregular shell structures and impaired catalytic function^13^. In such systems where cargo enzymes direct shell construction, premature closure to yield empty or underloaded compartments is unlikely and lumenal recognition, particularly at the shell protein junction region, is well-suited for coordinated assembly. Meanwhile, LS represents a different case. Without guest templates, LS shell proteins spontaneously form native-like, homogeneous assembly with desired geometry. Notably, we have not observed disassembled fragments when AaLS is produced recombinantly^22^, unless substantial mutagenesis or other remodeling is implemented^33,48–50^, indicating that the cage formation proceeds rapidly. This self-standing, quick assembly without guest templating raises the potential issue for formation of empty or insufficiently loaded cages, which are functionally unfavorable. The CLS anchoring mechanism, together with anti-cooperative behavior in late stage assembly, stabilizes incomplete cage assemblies to extend the window for guest packaging, providing a solution for preventing underloaded compartment formation.

While offering advantage for cargo loading, continued delay in LS cage completion raises the risk of impaired substrate channeling. The accumulation of cargo-loaded cage intermediates suggests that the system likely prioritizes avoiding empty or underloaded compartment formation, which would waste cellular resources by synthesizing enzymes in catalytically unfavored configurations. Beyond the thermodynamics and the molecular lock, nature has possibly evolved another layer of regulatory mechanism for immediate cage closure once the guest stoichiometry matures, e.g., RS timers recruiting the final pentamer, while such structures were not identified in the present cryo-EM analysis. Notably, AaRS/AaLS complex characterized in this study was obtained by heterologous production in *E. coli*, which does not fully recapitulate native cellular conditions, including gene expression levels. Questions also remain regarding stoichiometry precision requirement for sufficient vitamin B2 biosynthesis as organisms may tolerate some degree of incomplete cage formation. Structural and functional characterization in native biological contexts would advance understanding of how RS/LS assembly system coordinates stoichiometric control with catalytic performance.

While RS/LS complex formation is an evolutionary solution to enhance catalytic efficiency, organisms employ many other approaches to secure vitamin B2 supply. Uptake from the environment represents one such mechanism, as surveys of 1133 bacterial genomes reveal that 30% encode at least one riboflavin transporter^51^. For some species, e.g., *Staphylococcus pyogenes*^52^, uptake fully replaces biosynthesis, while other organisms, including *Bacillus subtilis*^53^ and *Lactococcus lactis*^54^, maintain both routes, which is likely advantageous for temporal environmental variability. Increasing local substrate availability may provide another strategy to enhance riboflavin biosynthesis, as demonstrated by plants that localize riboflavin biosynthetic enzymes in chloroplasts^55^, potentially facilitating the condensation of pathway intermediates. Supporting this approach, metabolic engineering studies have shown that increasing GTP supply, the precursor for 5-amino-6-(D-ribitylamino)uracil (1) in the pathway (Fig. 1a), significantly enhances riboflavin production^56,57^. These diverse vitamin acquisition pathways, together with the modular nature of the CLS peptide, constitute a rationale for the observed evolutionary lability of this compartmentalization mechanism (Fig. 4c).

Natural enzyme compartmentalization systems have inspired substantial efforts to construct biomimetic synthetic counterparts^48,58–65^. These approaches typically involve repurposing or re-engineering natural protein cages, such as viral capsids, to organize enzymatic reactions spatially. Despite these advances, achieving defined guest enzyme stoichiometry at the single-particle level remains a key challenge, as current synthetic systems yield only population-averaged loading with substantial heterogeneity that compromises catalytic precision^66^. The hierarchical assembly mechanisms revealed here in the riboflavin biosynthesis compartment provide molecular design principles for rational engineering of next-generation biomimetic catalytic systems with precise stoichiometric control, advancing designer metabolic pathways and programmable cellular factories.

## Methods

### Materials

All the salts and buffer components were purchased from Merck-Millipore (Burlington, MA, USA), New England BioLabs (Ipswich, MA, USA), or Thermo Fisher Scientific (Waltham, MA, USA) unless specified. Oligonucleotides were synthesized by Merck-Millipore. *E. coli* strains BL21-Gold(DE3) and DH5α competent cells were purchased from Agilent (Santa Clara, CA, USA) and Thermo Fisher Scientific, respectively. The plasmid pMG_AaLS-wt, pACYC_Ptet_AaRS, and pACYC_Ptet_sfGFP-AaRS196-207 were kindly provided by Prof. Donald Hilvert (ETH Zurich, Switzerland)^22^. All proteins, plasmids, and oligonucleotides used in this study are listed in Supplementary Tables 4–6, respectively.

### Molecular cloning

The plasmids for producing AaLS mutants containing a single alanine were prepared by quick change polymerase chain reaction using pET15_AaLS-wt-Strep^67^ as a template and a pair of oligonucleotides, FW_AaLS_XX and RV_AaLS XX, where XX corresponds to the introduced mutation (Supplementary Table 6), as primers. The codon-optimized LS genes encoding BaLS, CtLS, HpLS, SaLS, TmLS, or SoLS, with a C-terminal Strep-tag, and the RS genes encoding BaRS, CtRS, HpRS, SaRS, TmRS, or SoRS were synthesized and subcloned into pET15 or a pACYC_Ptet vector both via NdeI/XhoI sites, performed by GenScript Biotech B.V. (Rijswijk, Netherlands). *E. coli* strains DH5α were used as the host cells for every cloning step. Sequences of plasmids were confirmed by DNA Sanger sequencing performed by Eurofins Genomics Europe Sequencing GmbH (München, Germany).

### Production and purification of AaRS/AaLS complex^22^

*E. coli* strain BL21(DE3)-gold that transformed with plasmids pMG_AaLS-wt and pAYC-Ptet_AaRS were cultured in LB medium (50 mL) until OD600 reached 0.6–0.8, at which protein production was induced by 0.2 mM isopropyl β-_D_-1-thiogalactopyranoside (IPTG) and 5 µg/mL tetracycline. After culturing at 25 °C for 18–20 h, cells were harvested by centrifugation at 5000 × *g* and 4 °C for 10 min and stored at −20 °C until purification. Cell pellet was resuspended in 25 mL of 0.1 M sodium-phosphate (Na-Pi) buffer (pH 8.0) containing 200 mM NaCl, supplemented with 0.5 mg/mL lysozyme and 5 µg/mL DNase I. After bacteriolysis using sonication, the insoluble fraction was removed by centrifugation at 9500 × *g* and 25 °C for 25 min. AaLS protein, which possesses a His6 tag at the C-terminus, was purified from the resulting supernatant by affinity chromatography using a HisPur Ni-NTA resin (Thermo Fisher Scientific). Washing was performed with 50 mM Na-Pi buffer (pH 8.0) containing 200 mM NaCl and 20 mM imidazole, and protein was eluted using 50 mM Na-Pi buffer (pH 8.0) containing 200 mM NaCl and 500 mM imidazole. The buffer was then replaced with 50 mM Na-Pi buffer (pH 8.0) containing 200 mM NaCl and 5 mM ethylenediaminetetraacetic acid (EDTA) using Amicon Ultra centrifugal units (50 kDa MWCO). The resulting solution was subjected to size-exclusion chromatography using a Superose 6 increase 10/300 column (Cytiva) running with 50 mM Na-Pi buffer (pH 8.0) containing 200 mM NaCl and 5 mM EDTA at room temperature (RT) and flow rate of 0.75 mL/min. Peak fractions were pooled, concentrated, and kept at RT until further experiments. Protein concentration was determined by absorbance at 280 nm using the extinction coefficient calculated for AaLS (*ε*_280_ = 13,980 M^−1^cm^−1^). Protein purity as well as complex formation with AaRS were confirmed by sodium dodecyl-sulfate polyacrylamide gel electrophoresis (SDS-PAGE) with Coomassie R350 staining.

AaLS alone was produced and purified using the same protocol with cells harboring only pMG_AaLS-wt and induced with 0.2 mM IPTG. For AaRS alone, cells harboring only pAYC-Ptet_AaRS were induced with 5 µg/mL tetracycline and processed through cell lysis as described above. The clarified lysate was filtered (0.22 μm) and subjected to ammonium-sulfate precipitation. The precipitated protein was collected by centrifugation, dissolved in 10 mM Tris-HCl (pH 8.9), desalted on a PD-10 column (Cytiva), and purified by anion-exchange chromatography using a HiTrap Q HP column (Cytiva) with elution over a linear 0–50% gradient of 1 M NaCl in 10 mM Tris-HCl (pH 8.9). Peak fractions were pooled, buffer-exchanged into 10 mM Tris-HCl (pH 8.9), and further polished on a Mono Q 5/50 GL column (Cytiva). The purified protein was then subjected to size-exclusion chromatography on a Superdex 200 Increase 10/300 column (Cytiva) equilibrated in 50 mM Na-Pi buffer (pH 8.0) containing 200 mM NaCl and 5 mM EDTA. Peak fractions were pooled, concentrated, and used for mass photometry.

### Mass photometry of AaRS

Mass photometry: Measurements were performed on a Refeyn Ltd. OneMP mass photometer. Borosilicate coverslips (#1.5, 24 × 50 mm²; Thorlabs) were cleaned by sequential sonication in water, isopropanol, and water, and dried under a nitrogen stream. Clean silicone gaskets were mounted onto the coverslips, and 9 μL of measurement buffer was added for focusing. Purified AaRS was diluted in the same buffer to approximately 10–30 μg/mL, and 1 μL of the diluted sample was introduced immediately prior to acquisition. Movies were recorded at 100 frames s⁻¹ using 5× frame-binning and 4× pixel-binning, with a total of 100,000 frames collected per measurement. Raw data were processed in DiscoverMP v2.0.3 using default parameters, with averaging and reflectivity correction enabled, and molecular-mass distributions were obtained from calibrated contrast values.

### Cryogenic electron microscopy (cryo-EM) grid preparation

Purified LS cages were concentrated using an Amicon Ultra centrifugal unit (30 kDa MWCO) to 1 mg/mL, and 3.5 μL of the sample was applied to freshly glow-discharged Quantifoil R2/1 Cu 200 mesh grids (Quantifoil Micro Tools GmbH, Großlöbichau, Germany). For the AaRS/AaLS inclusion complex, 0.5 mg/mL protein and a grid coated with graphene oxide were used. Grids were vitrified in liquid ethane using a Vitrobot Mark IV (Thermo Fisher Scientific) at 100% relative humidity and 4 °C. Vitrification parameters included a wait time of 30 s, blot total of 2, blot force of 3, and blot time of 3 s.

### Cryo-EM data collection for single-particle reconstruction

Cryo-EM movie datasets were acquired on a Titan Krios G3 microscope (Thermo Fisher Scientific) at the National Synchrotron Radiation Centre in Poland (SOLARIS), operated at 300 kV accelerating voltage with a nominal magnification of ×105,000 and a calibrated pixel size of 0.8456 Å per pixel. A K3 direct electron detector (Gatan, Pleasanton, CA, USA), equipped with BioQuantum Imaging Filter using a 20-eV slit, was operated in counting mode. Imaged areas were exposed to a total dose of 40e⁻/Å², corresponding to a dose rate of ~16e⁻/pixel/s. Forty-frame movie stacks were recorded under defocus values ranging from 0.9 to 2.1 μm, using 0.3μm increments. The number of movies collected per dataset ranged from 5000 to 10,000 (Supplementary Figs. 1 and 3). The collected datasets were processed using cryoSPARC v4.5.3^68^.

### Cryo-EM single-particle reconstructions of AaRS/AaLS inclusion complexes

A dataset of 8625 movies was processed using patch motion correction and patch CTF estimation. Subsequent 3D template-based particle picking yielded 2,539,751 2× binned particles, which were subjected to 2D classification, resulting in 1,749,455 (100% of the dataset) being selected (Supplementary Fig. 1a). An ab initio reconstruction was performed without imposed symmetry (C1), followed by homogeneous 3D refinement using tetrahedral (T) symmetry with symmetry relaxation into C1^69^. The resulting 12-pentamer cage was used to generate a soft 3D mask encapsulating the shell of the assembly. The corresponding particles were then subjected to four rounds of 3D classification into ten classes at 4 Å resolution, with the soft mask applied. Manual inspection and reassignment of particles between classes, based on structural features, resulted in five groups: a 12-pentamer cage with icosahedral (I) symmetry, an 11-pentamer cage with C5 symmetry, a 10-pentamer cage with C2 symmetry, a 9-pentamer cage with C3 symmetry, and classes representing unresolved or heterogeneous states. A subset of 732,976 particles (42%) corresponding to the 12-pentamer cage was unbinned, reference-based motion corrected, and refined with I symmetry. The 288,354 (16%) and 135,997 (8%) particles assigned to the 11- and 10-pentamer cages, respectively, were subjected to downstream analyses, while the remaining particles were discarded. The 11- and 10-pentamer particle subsets were not completely homogeneous, as residual cryo-EM density from the missing pentamers remained visible at higher volume threshold levels (Supplementary Fig. 1b, c). Soft 3D masks were generated to cover the locations of the absent pentamers. The particles were then subjected to two rounds of 3D classification into ten classes, at 8 Å followed by 3 Å resolution, with the soft mask applied. Selected particles were unbinned, reference-based motion corrected, and refined; in the case of the 11-pentamer cage, they were further classified into five classes at a 3 Å resolution. Local refinement of the resulting 116,229 (7%) and 81,039 (5%) particles revealed C5- and C2-symmetrized C-termini of AaRS associated with the 11- and 10-pentamer cages, respectively. These subsets were symmetry-expanded according to their respective symmetries, and the signal corresponding to the AaLS shell was subtracted using 3D masks. Unmasked 3D classification of the resulting particles revealed the unsymmetrized AaRS trimer (Supplementary Fig. 1b, c), which was used to generate soft 3D masks. The particles were then subjected to 3D classification into ten classes at 8 Å resolution, followed by classification into five classes and 3 Å in the case of the 11-pentamer cage. Following AaLS shell “unsubtraction” (i.e., the assignment of the shift and orientation parameters on the original, unsubtracted particles), the final subsets consisted of 25,459 (1%) and 17,809 (1%) particles, respectively, which were subjected to final local refinement without imposed symmetry (C1). Global and local resolution of the final volumes was assessed using 0.143 Fourier shell correlation (FSC) cut-off (Supplementary Figs. 2, 15 and Supplementary Table 7).

### Cryo-EM single-particle reconstruction of empty AaLS cages

Datasets collected for wild-type AaLS and its mutants (Supplementary Fig. 3) were processed as described above, with minor modifications. The particles were not subjected to symmetry expansion and subsequent signal subtraction. The final reconstruction yielded 12-pentamer cages with icosahedral (I) symmetry and 11-pentamer cages with C5 symmetry. Global and local resolution of the final volumes was assessed using 0.143 FSC cut-off (Supplementary Figs. 6 and 15 and Supplementary Table 8).

### Molecular modeling

The initial atomic model of AaLS was derived from the X-ray crystal structure of wild-type AaLS at 1.6 Å resolution (PDB: 1HQK)^17^. The structure of AaRS trimer was predicted using AlphaFold 3^70^. Rigid-body fitting of the initial models into cryo-EM maps was performed using ChimeraX v1.10.1^71^, followed by manual adjustment in WinCoot v0.9.8.96^72^. Flexible fitting was conducted using ISOLDE v1.9^73^. Real-space refinement of the models was carried out with Phenix v1.20.1-4487^74^. Model validation was performed using MolProbity^75^, and the statistics are presented in Supplementary Tables 7 and 8. Structures were visualized using ChimeraX. For visualization purposes in Fig. 1l, the unsharpened map of the symmetry-expanded 11-pentamer cage was post-processed using DeepEMhancer in highRes mode^76^.

### Pulldown assay

The complex formation of GFP-CLS and different AaLS mutants were assessed by their coproduction in *E. coli* cells, followed by a pulldown assay, using an analogous procedure previously described^22^. *E. coli* BL21(DE3)-gold cells were transformed with pACYC_GFP-CLS and a pET15 vector encoding corresponding AaLS mutant possessing a StrepII tag at the C-terminus. Cells were then cultured at 37 °C in LB medium (6 mL) containing 100 µg/mL ampicillin and 35 µg/mL chloramphenicol until OD_600_ reached 0.6–0.8, at which point protein production was induced with 0.2 mM IPTG and 2 µM tetracycline. After culturing at 25 °C for 20 h, cells were harvested by centrifugation (4500 × *g*, 4 °C, 10 min) and stored at −20 °C until purification. The cell pellet was resuspended in 400 µL of 2× phosphate-buffered saline (PBS) containing 1 mM EDTA, referred to as 2 × PBS-E. After bacteriolysis by sonication, the insoluble fraction was removed by centrifugation (22,500 × *g*, 20 °C, 5 min). The Strep-tagged AaLS protein was purified using StrepTactin HP resin ( ~ 100 µL as 50% suspension) in a 0.5-mL spin column. The resin was washed with 2 × PBS-E (200 µL × 3), followed by elution with 2 × PBS-E containing 2.5 mM D-biotin (35 µL × 3), and used for native AGE analysis.

For other RS/LS complexes, pET28 carrying a corresponding LS gene and pACYC_Ptet vectors encoding the cognate RS were used. Cell culture was performed in LB medium supplemented with 50 µg/mL kanamycin and 35 µg/mL chloramphenicol. All the other procedures correspond to the above-described AaLS-based pulldown assay. Protein concentration was determined by absorbance at 280 nm using the extinction coefficient calculated for corresponding LS (Supplementary Table 4).

### Native agarose gel electrophoresis (AGE)

Samples were prepared in PBS-E and added with a 4× native-PAGE loading buffer [0.2 M BisTris-HCl buffer (pH 7.2) containing 40% (v/v) glycerol, 0.016% (w/v) bromophenol blue]. Electrophoresis was performed using 1.2% (w/v) agarose gels and a running buffer [0.2 M Tris base, pH adjusted with phosphoric acid to ~7.6] at R.T. and 70 V for 40 min. Fluorescent bands were visualized using a ChemiDoc MP (Bio-Rad, Hercules, CA, USA) in the fluorescein detection mode (460–490 nm excitation and 532/28 nm emission filters). The same gels were stained with ReadyBlue Protein Gel Stain (Merck-Millipore). The band intensities were quantified using Image J, and the ratio between fluorescent and ReadyBlue imaging was used to estimate GFP association efficiency. The data are presented as relative to that for the wild-type protein using the following Eq. (1),

1 $${\rm{Relative}}\; {\rm{GFP}}\; {\rm{band}}\; {\rm{intensity}}(\%)=(I{fl}/I{RB})/(I{fl},{wt}/I{RB},{wt})\times 100$$

where *I*_fl_ and *I*_RB_ represent the observed band intensity of interest from fluorescent and ReadyBlue staining, respectively; *I*_fl,wt_ and *I*_RB,wt_ are those obtained with AaLS-wt, which was analyzed in the same gel. Experiments were performed in quadruplicate, and means ± standard deviation, along with individual data points, are shown in Fig. 2e.

### Rosetta interface and local energy analysis

Residue-level contributions of the AaRS CLS to the AaLS pre-C3 binding site were evaluated using Rosetta v2025.33 with the REF2015 and REF2015_cart all-atom score functions^77,78^. The CLS-bound C5 11-pentamer structure (PDB ID: 9RYJ) was used to define the native binding geometry. A local model containing the CLS and the two AaLS pentamers that contact it was extracted. Equivalent regions from the empty 11-pentamer cages of wild-type AaLS (PDB ID: 9RYO) and the R29A, L121A, and I125A mutants (PDB IDs: 9RYQ, 9RYV, 9RYX) were aligned to this template, after which the CLS coordinates were transplanted to impose an identical cryo-EM–derived binding pose in all backgrounds.

For each background, the extracted CLS–AaLS assembly was subjected to restrained relaxation in Rosetta using Cartesian and torsional FastRelax protocols, with coordinate constraints applied to preserve proximity to the experimental geometry. Structural flexibility was allowed only in the CLS and in the full AaLS protomers that interact with it, while all remaining regions were kept fixed. Following relaxation, total interface energies (ΔG_Rosetta_) and buried hydrophobic solvent-accessible surface area (ΔSASA_hydrophobic_) were obtained using InterfaceAnalyzer. Residue-level interaction energies were taken directly from the interface_energy application, which reports, for each CLS residue, its total pairwise interaction energy with all residues of the contacting AaLS protomers. Mutant effects were quantified as;

2 $$\Delta \Delta {\rm{G}}\scriptsize{\rm{R}}{\rm{osetta}}\normalsize=\Delta {\rm{G}}\scriptsize{{\rm{R}}{\rm{osetta}},{\rm{WT}}}\normalsize -\Delta {\rm{G}}\scriptsize{\rm{R}}{\rm{osetta}},{\rm{mut}}$$

Local free energy analysis was performed using the Rosetta residue_energy_breakdown application with the REF2015_cart score function. Cartesian relaxation was applied to the corresponding molecular models, followed by per-residue total score calculation. Local loop energy was estimated as the sum of the values for the residues 129–133.

Binding energetics for CLS or AaLS protomer docking into a pre-C3 region were evaluated using Rosetta InterfaceAnalyzer, as described above. Total ΔSASA (polar + hydrophobic) was taken into account for this comparison (Fig. 3e, f).

### Molecular dynamics (MD)

MD simulations were performed using the AMBER22 software suite^79^. The cryo-EM atomic model (C5 symmetry) of the AaRS/AaLS complex was used as the initial structure. For the R29A mutant, the CLS peptide was positioned on the atomic model of the 11-pentamer assembly in the same orientation as in the wild-type structure. Hydrogen atoms were added by the LEaP module of AMBER20. The simulation system contains CLS of AaRS and two pentamers of AaLS placed in a water box of approximately 157 Å × 134 Å × 106 Å. To neutralize the system, 40 or 50 sodium ions were put in the box. AMBER ff14SB sets and TIP3P were utilized for the protein and water molecules, respectively. Long range electrostatic interactions were treated by the particle mesh Ewald method. Non-bonded interactions were cut off at 10 Å. After carrying out a short minimization to remove artificial repulsions in the initial structure, 1.0 µs MD simulations in a constant-NPT (300 K, 1 atm) ensemble were performed after the 100 ps heating stage with NVT ensemble (the time step is 2.0 fs and hydrogen atoms were constrained with SHAKE procedure). At the heating step, the temperature was raised gradually from 0 K to 300 K with the weak restraints (10 kcal/mol/A^2^) to the atoms of the designed protein. We performed MD simulations three times for each of the wild-type and mutant. The root mean square deviation (RMSD) values were calculated using the cpptraj module in AmberTools20 to evaluate equilibration (Supplementary Fig. 16). Based on the RMSD analysis, interaction energies were calculated using the cpptraj module of AmberTools20 over the final 500 ns of the total 1.0 µs simulation.

### SDS-PAGE for RS/LS complex formation

Samples were prepared in an SDS-PAGE sample buffer [62.5 mm Tris-HCl buffer (pH 6.8) containing 2.5% (w/v) sodium dodecyl sulfate (SDS), 10% (v/v) glycerol, 5% (v/v) β-mercaptoethanol, 0.2 mM dithiothreitol, and 0.004% (w/v) bromophenol blue] and heated at 95 °C for 10 min. Electrophoresis was performed using 2 µg proteins, 15% polyacrylamide gels, unless specified, and a Tris-Tricine buffer system at 80 V for 10 min followed by 150 V for 60 min. As a protein standard, PageRuler™ Prestained Protein Ladder (Thermo Fisher Scientific) was used. Protein bands were visualized using ReadyBlue Protein Gel Stain. The band intensity ratio between RS and LS was used to estimate RS encapsulation efficiency. Experiments were performed in triplicate, and means ± standard deviation, along with individual data points, are shown in Fig. 4e.

### Cryo-EM of lumazine synthase orthologs

Purified cages were concentrated and cryo-EM grids prepared as described above for AaLS, with the cages concentrated to 0.5 mg/mL. The cryo-EM micrographs were acquired on a Glacios microscope (Thermo Fisher Scientific) fitted with a Falcon 4 detector operated at 200 kV accelerating voltage, magnification of ×190,000, and corresponding pixel size of 0.73 Å/px. The collected micrographs were analyzed using Fiji^80^.

### Organism and gene selection

Organisms for evolutionary analysis were initially selected based on a previous report that predicted the quaternary state of LSs^81^. The dataset was subsequently expanded to increase taxonomic diversity. Additional microorganisms were manually selected to ensure balanced representation across categories of riboflavin biosynthetic requirement and availability levels.

LS and RS genes were identified by searching the NCBI Genome database (https://www.ncbi.nlm.nih.gov/datasets/genome/), prioritizing reference and representative genomes where available. Sequences annotated as “LS” and “RS” were extracted. For organisms harboring multiple LS and/or RS genes, gene pairs located within the same operon were prioritized for analysis. All genome and gene identifiers are provided in Supplementary Tables 1 and 2.

### RS/LS complex formation prediction

RS/LS complex formation was predicted using AlphaFold 3 (https://alphafoldserver.com/)^70^. Guided by the experimentally observed AaRS/AaLS architecture, three copies of RS (homotrimer) and fifteen copies of LS (three pentamers) were used as input to capture the CLS peptides binding at the pre-C3 interface. For plant variants, the N-terminal chloroplast localization signal peptide was removed prior to modeling, with the cleavage site determined by multiple sequence alignment as described in the Phylogenetic analysis section. For each variant, three independent AlphaFold runs were performed using distinct random seeds, and each run produced five models (15 models total per variant). All predicted structures were manually inspected. A variant was classified as complex-forming if at least one model exhibited the expected CLS–pre-C3 interaction interface between LS and RS. A prediction score was also calculated as the fraction of the 15 models supporting the canonical CLS–pre-C3 interface, with each supporting model contributing 1/15 to the score.

### Taxonomic analysis

The taxonomic tree was constructed using the NCBI Taxonomy Common Tree tool (https://www.ncbi.nlm.nih.gov/Taxonomy/CommonTree/wwwcmt.cgi) and visualized using FigTree v1.4.4 (http://tree.bio.ed.ac.uk/software/figtree/). The tree was annotated to display the distribution of AlphaFold 3-predicted complex-forming and non-complex-forming variants across different taxonomic groups.

### Phylogenetic analysis

Phylogenetic analysis was performed using RS protein sequences. Multiple sequence alignment was conducted using MUSCLE as implemented in MEGA v12 software^82^, where the N-terminal chloroplast-localization signal peptides for the plant variants were trimmed. The optimal evolutionary model was determined using the Model Selection feature in MEGA, with the LG + G + I model (Le and Gascuel substitution model with gamma-distributed rate variation and invariant sites) selected based on the lowest Bayesian Information Criterion score. The maximum likelihood phylogenetic tree was constructed using the LG + G + I model with 1000 bootstrap replicates. The phylogenetic tree was visualized and annotated using FigTree v1.4.4 to display the distribution of AlphaFold 3-predicted complex-forming and non-complex-forming variants.

### Riboflavin requirement/availability analysis

To enable consistent cross-species comparison, riboflavin requirement and environmental riboflavin availability were coded into three discrete categories (Low, Mod, High) using explicit deterministic rules.

Availability reflects access to extracellular riboflavin (riboflavin/FMN/FAD) via cross-feeding or environmental vitamin pools. Availability was scored High only for clearly vitamin-rich niches characterized by dense metabolic exchange (e.g., mammalian gut and oral cavity, fermented foods/dairy fermentations, plant rhizosphere/compost, engineered dense consortia). Availability was scored Low for oligotrophic or extreme environments with negligible vitamin inflow (e.g., deep ocean/sediments, vents, thermal/acidic/hyper-saline systems, nutrient-poor soils). All other environments (typical soils, aquatic mesotrophic systems, respiratory mucosa, intracellular habitats with partial host supply) were scored as Mod. Riboflavin requirement reflects intrinsic demand for flavin cofactors (FAD/FMN) driven by routine metabolism.

Requirement was scored High when a major flavin-intensive driver was present: photosynthetic electron transport, nitrogen fixation, bioluminescence, thermophilic high-flux respiration, extremely rapid growth, or extensive secondary metabolism. Requirement was scored Low for genome-reduced obligate intracellular symbionts, minimal fermenters, and very slow extremophiles with streamlined redox throughput. All remaining general heterotrophs, facultative pathogens/commensals, and typical fermenters were scored Mod. When evidence was mixed, assignment followed the dominant routine lifestyle (primary niche + primary energy metabolism). All the categorization and its rationale are summarized in Supplementary Table 3.

### Reporting summary

Further information on research design is available in the Nature Portfolio Reporting Summary linked to this article.

## Supplementary information

Supplementary Information

Description of Additional Supplementary Files

Supplementary Movie 1

Reporting Summary

Transparent Peer Review file

## Source data

Source Data

## Data Availability

Cryo-EM micrographs, reconstructions, and atomic models have been deposited in the Electron Microscopy Public Image Archive, the Electron Microscopy Data Bank, and the Protein Data Bank under the following accession codes: EMPIAR-13016 (WT AaLS/AaRS assemblies), EMD-54381 and PDB 9RYI (12-pentamer RS-bound cage), EMD-54382 and PDB 9RYJ (11-pentamer RS C-termini (C5) cage), EMD-54383 and PDB 9RYK (11-pentamer RS-bound cage), EMD-54385 and PDB 9RYM (10-pentamer RS-trimer–bound cage); EMPIAR-13017 (WT AaLS), EMD-54386 and PDB 9RYN (12-pentamer cage), EMD-54387 and PDB 9RYO (11-pentamer cage); EMPIAR-13018 (R29A AaLS), EMD-54388 and PDB 9RYP (12-pentamer cage), EMD-54389 and PDB 9RYQ (11-pentamer cage); EMPIAR-13019 (L121A AaLS), EMD-54392 and PDB 9RYU (12-pentamer cage), EMD-54393 and PDB 9RYV (11-pentamer cage); EMPIAR-13020 (I125A AaLS), EMD-54394 and PDB 9RYW (12-pentamer cage), EMD-54395 and PDB 9RYX (11-pentamer cage). AlphaFold3 RS/LS complex formation prediction and MD simulation data are available from Rodbuk (10.57903/UJ/CVWGR3 (AlphaFold); 10.57903/UJ/TUNWSU (MD)). Source Data are provided as a Source Data file. Source data are provided with this paper.
