# Supplementary material for: A molecular basis for stoichiometric enzyme encapsulation in the vitamin B2 biosynthesis compartment
Source: Nat Commun. 2026 May 16;17:6498. doi: 10.1038/s41467-026-73260-4 (PMC13376624; doi:10.1038/s41467-026-73260-4)
Supplement: Supplementary file 1 — Supplementary Information [file 41467_2026_73260_MOESM1_ESM.pdf]

Supplementary Information for

A molecular basis for stoichiometric enzyme encapsulation in the vitamin B2 biosynthesis compartment

Lukasz Koziej[1], Jedrzej Pankowski[1,2†], Monika Stefanska[1†], Daniel Jankowski[1], Agnieszka Gawin[1], V. Vishal Malolan[1,3], Juha T. Huiskonen[4], Takahiro Kosugi[5,6,7‡], and Yusuke Azuma[1*]

1. Malopolska Centre of Biotechnology, Jagiellonian University, 30-387 Krakow, Poland
2. Faculty of Biochemistry, Biophysics, and Biotechnology, Jagiellonian University, 30-387 Krakow, Poland
3. Doctoral School of Exact and Natural Sciences, Jagiellonian University, 30-348 Krakow, Poland
4. Institute of Biotechnology, Helsinki Institute of Life Science HiLIFE, University of Helsinki, 00014 Helsinki, Finland
5. Research Center of Integrative Molecular Systems (CIMoS), Institute for Molecular Science (IMS), National Institutes of Natural Sciences (NINS), Okazaki, Aichi, 444-8585, Japan
6. Molecular Science Program, SOKENDAI (The Graduate University for Advanced Studies), Hayama, Kanagawa, 240-0193, Japan
7. PRESTO, Japan Science and Technology Agency, Kawaguchi, Saitama, 332-0012, Japan

† These authors contributed equally

‡ Current address: Faculty of Pharmacy, Institute of Medical, Pharmaceutical and Health Sciences, Kanazawa University, Kanazawa, Ishikawa, 920-1192, Japan

*E-mail: yusuke.azuma@uj.edu.pl

This file includes:

Supplementary Figs 1-16

Supplementary Tables 1-8

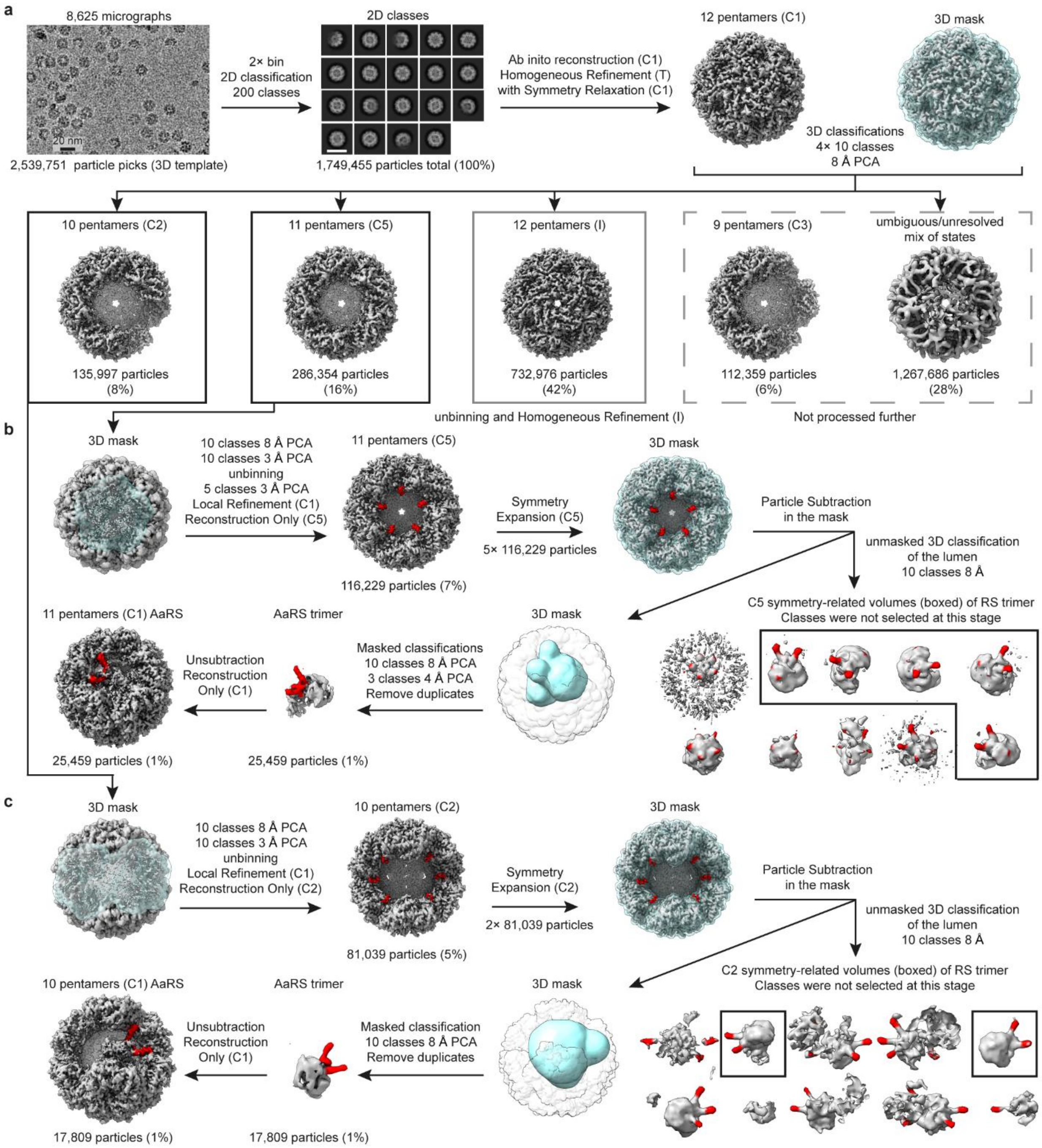

Supplementary Fig. 1: Cryo-EM single-particle reconstruction of AaRS/AaLS inclusion complexes. **a**, The schematic illustrates the cryo-EM data processing workflow, including 2D classification and masked 3D classification to obtain particles corresponding to fully assembled 12 pentamer icosahedral cages (I), as well as partial assemblies with 11 (C5), or 10 (C2) pentamers. **b**, Further processing of the 11-pentamer assembly. Focused 3D classification was used to remove residual particles exhibiting weak pentamer density. The cryo-EM density corresponding to the symmetrized (C5) C-termini of AaRS (highlighted in red) became visible after this procedure. Following C5 symmetry expansion, the cryo-EM density corresponding to the AaLS cage was subtracted from particles via masking (cyan). Subsequent classification revealed unsymmetrized (C1) AaRS trimer binding via two C-termini to the unoccupied pentamer-pentamer interaction interfaces. **c**, Processing of the 10-pentamer assembly, as in b but using C2 symmetry. The absolute and/or relative (% of total) number of particles is indicated after each selection step.

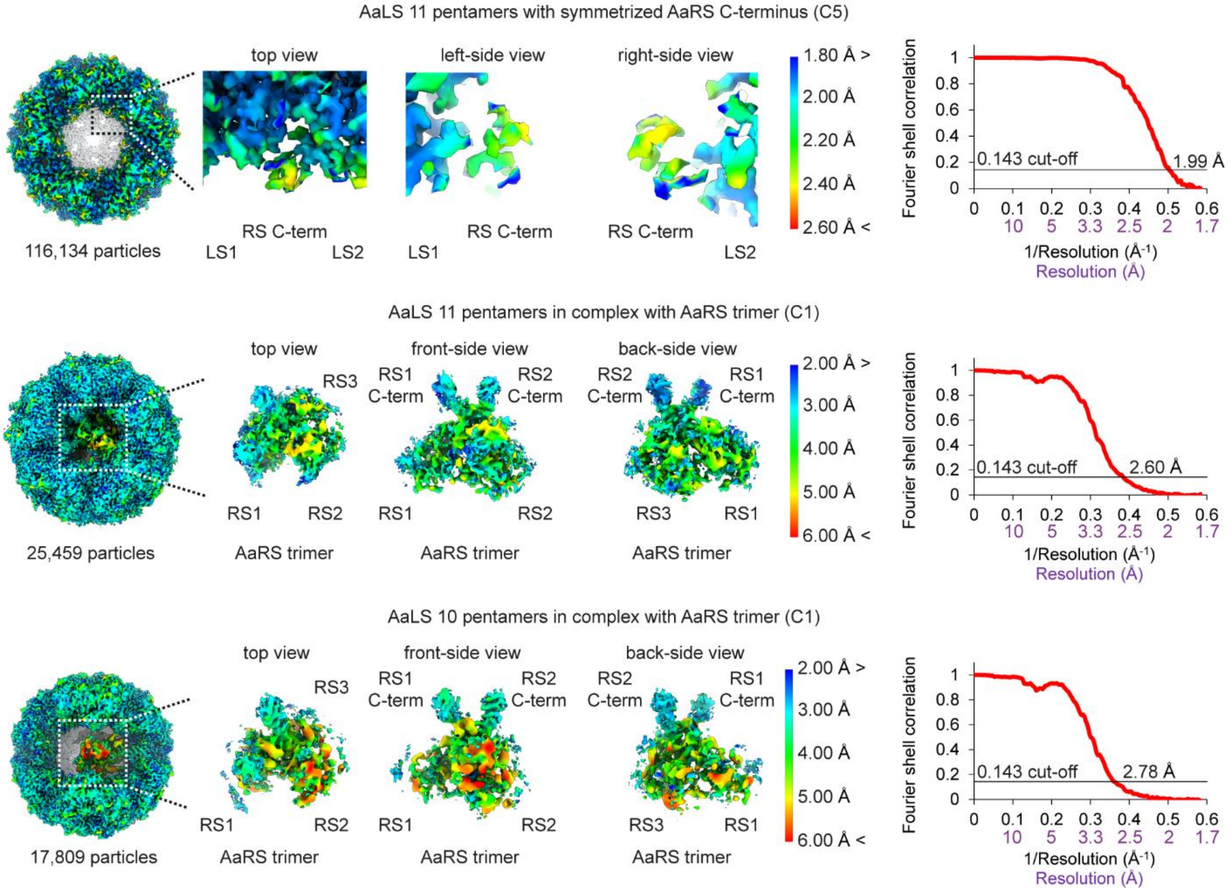

Supplementary Fig. 2: Local and global resolution estimation for cryo-EM reconstructions of AaLS cages with AaRS cargo. Shown from top to bottom are three assemblies: a C5-symmetrized 11-pentamer cage with five AaRS C-termini (top panels), and an unsymmetrized 11- (middle panels) and 10-pentamer (bottom panels) cages with the AaRS trimer. Maps are filtered and colored by local resolution, calculated using the 0.143 FSC cut-off. Particle numbers used for reconstruction are indicated below each map. Cages are oriented with the missing pentamers facing the viewer. For C5-symmetrized assembly (top panels), the insets show pentamer–pentamer interface at the AaRS C-terminal binding site, shown from the top, left (LS1), and right (LS2) monomer. For unsymmetrized assemblies (middle and bottom panels), insets highlight the AaRS trimer (monomers designated RS1, RS2, RS3) shown from top, front, and back views. A color vertical bar indicates the local resolution range. Gold-standard FSC(GS-FSC) curves for each reconstruction are presented in the rightmost panel, with global resolution values marked at the 0.143 FSC cut-off. Source data are provided as a Source Data file.

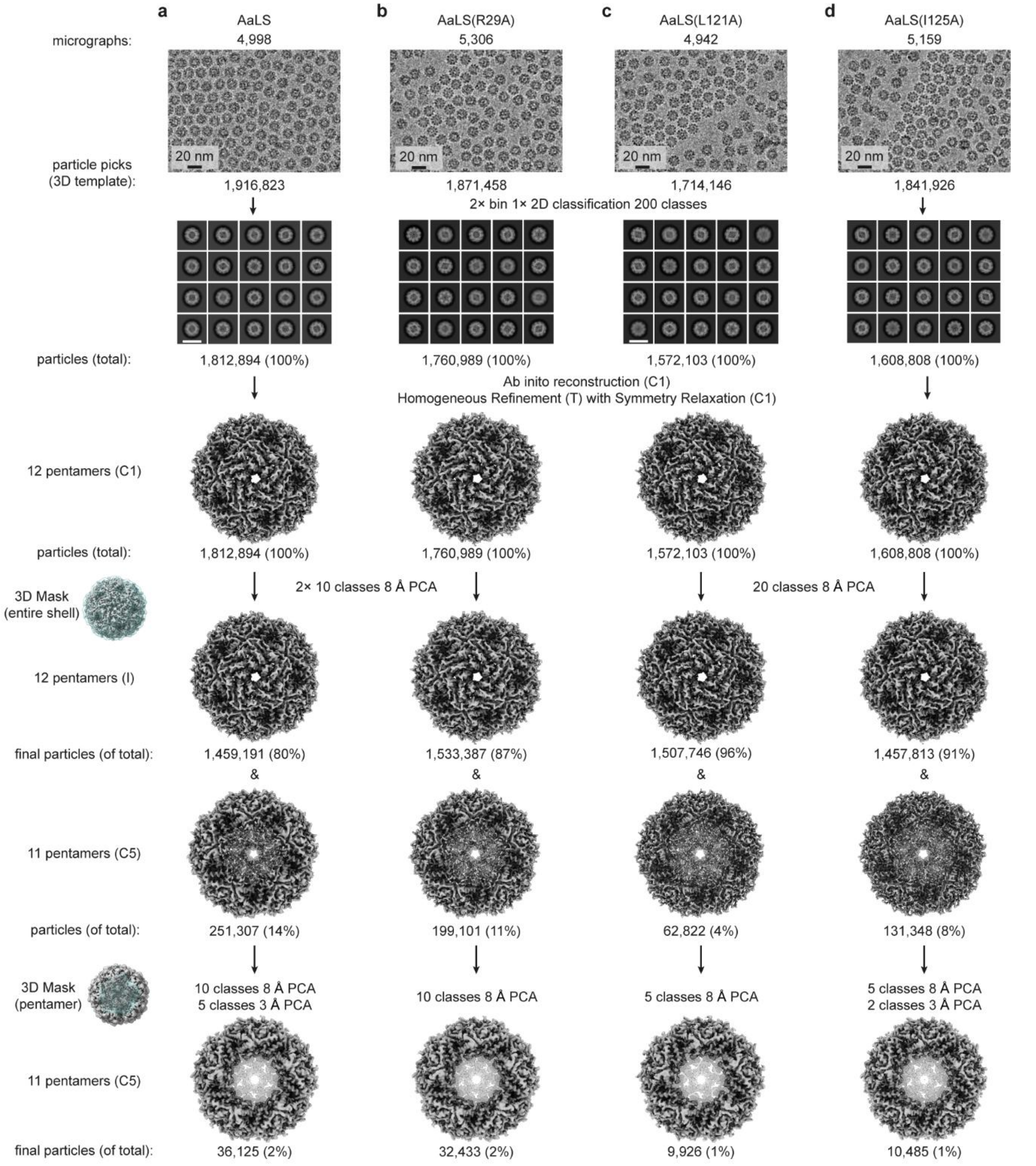

Supplementary Fig. 3: Cryo-EM single-particle reconstruction workflow for wild-type and interface-mutant AaLS assemblies. a-d, The schematic illustrates the processing of four cryo-EM datasets: AaLS (**a**), AaLS(R29A) (**b**), AaLS(L121A) (**c**), and AaLS(I125A) (**d**). The datasets were processed (from top to bottom) starting with 2D classification and masked 3D classification to obtain particles corresponding to fully assembled 12 pentamer icosahedral (I) cages, as well as partial assemblies with 11 pentamers (C5). The assemblies with 11 pentamers were processed further by focused 3D classification to remove residual particles exhibiting weak pentamer density. The absolute and relative (% of total) number of particles is indicated after each selection step.

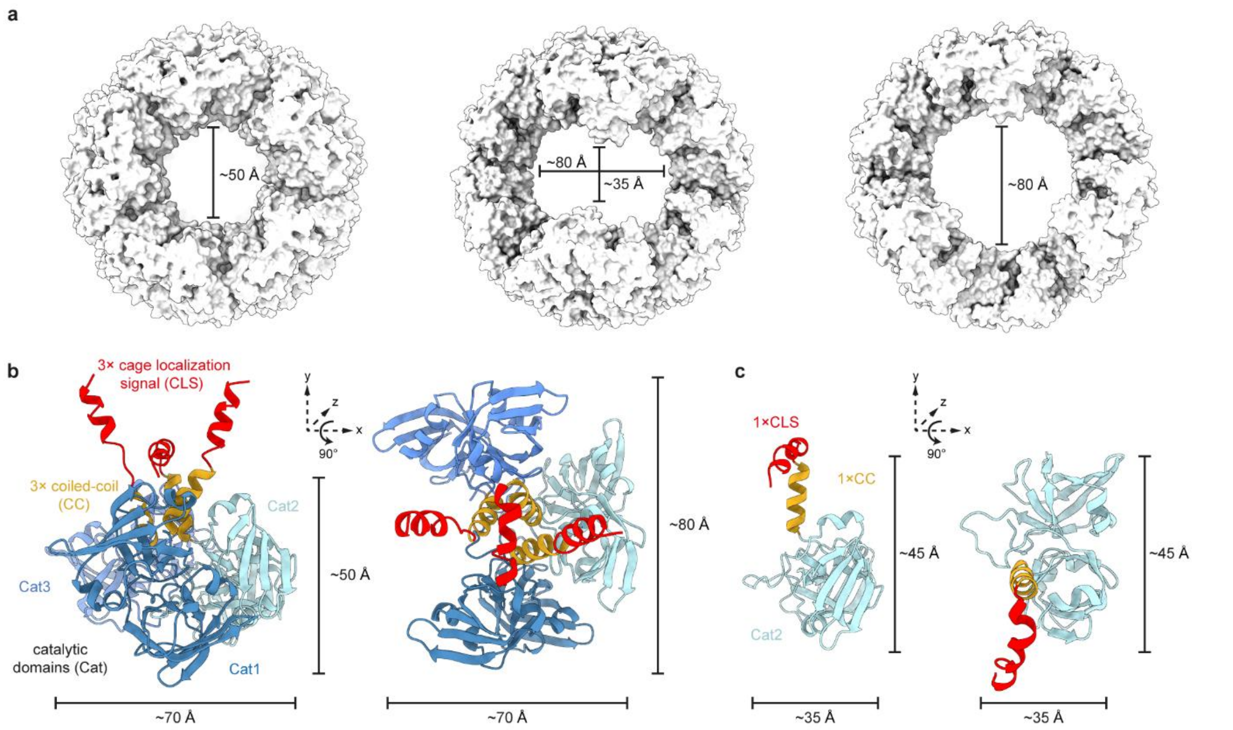

Supplementary Fig. 4: Size comparison between AaLS cage pores and guest AaRS. a, Pore dimensions of partially assembled AaLS cages shown for 11-pentamer (left), 10-pentamer (middle) and 9-pentamer (right) assemblies, with approximate pore diameters indicated (Å). **b,** AaRS homotrimer in cartoon representation shown in two orientations, with overall dimensions indicated; CLS shown in red, coiled-coil (CC) in orange, and catalytic domains in blue. **c,** AaRS monomer shown in two orientations for comparison, colored as in b. Comparison of RS dimensions with LS pore sizes indicates that entry of the AaRS trimer is geometrically compatible with the 9-pentamer assembly, whereas the AaRS monomer is compatible with the 11-pentamer pore.

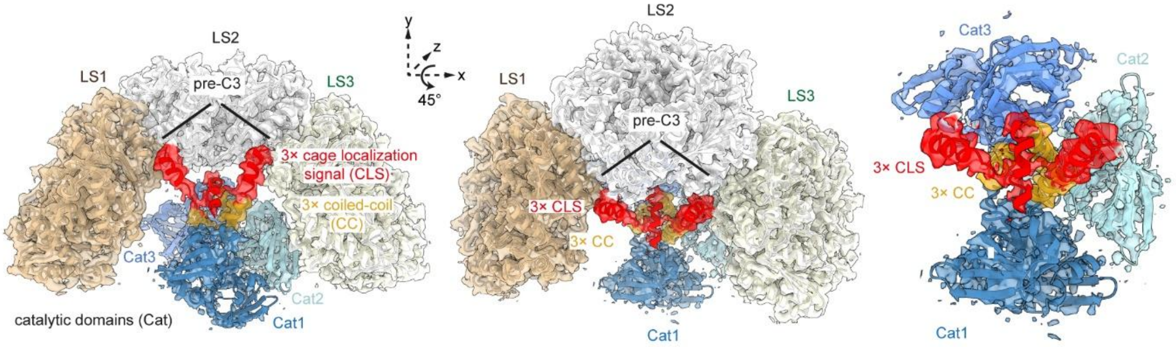

Supplementary Fig. 5: AaRS homotrimer fitted in cryo-EM density of the AaLS/RS inclusion complex. Left, view of three interacting AaLS pentamers (LS1–LS3) from the symmetry-expanded 11-pentamer assembly (PDB ID: 9RYK), corresponding to that shown in Fig. 1l but using a map without post-processing. Pentamers are shown in distinct white–brown shades, and the AaRS cage-localization signal (CLS), coiled-coil (CC) and catalytic domains (Cat) are shown in red, orange and blue, respectively. CLS binding at the pre-C3 region is indicated. Middle, rotated view. Right, enlarged view of the same orientation with LS1–LS3 pentamers omitted.

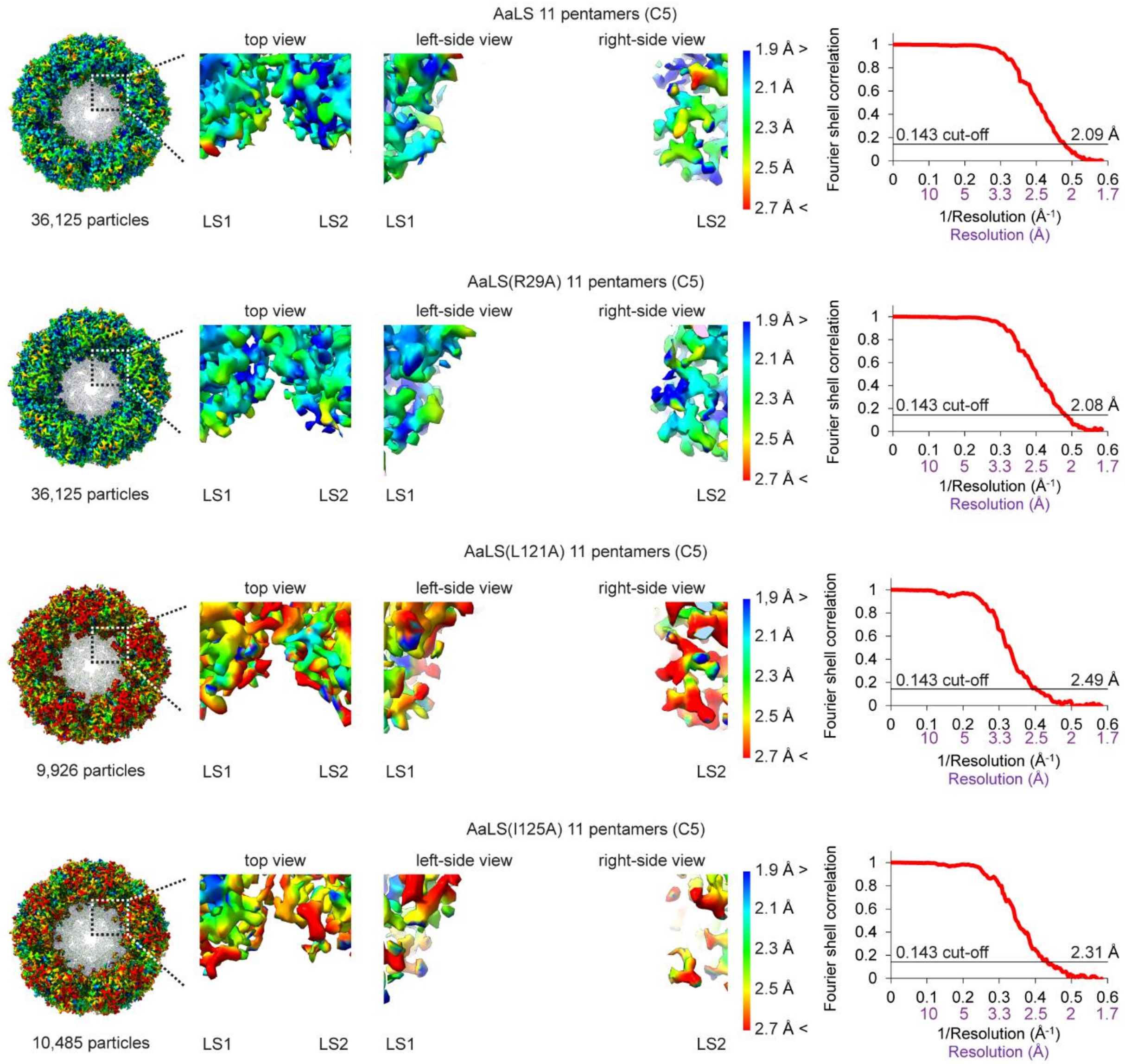

Supplementary Fig. 6: Local and global resolution estimation for cryo-EM reconstructions of 11-pentamer AaLS assemblies from wild-type and interface mutants. Shown are four C5-symmetrized AaLS assemblies (panels from top to bottom): AaLS, AaLS(R29A), AaLS(L121A), and AaLS(I125A). Maps are filtered and colored by local resolution, calculated using the 0.143 FSC cut-off. The number of particles used for each reconstruction is indicated below each map. Cages are oriented with the missing pentamer facing the viewer. Insets magnify the pentamer–pentamer interface to assess the local resolution at the binding site of the AaRS C-terminus, shown from the top of the pentamer, and from the side of the left (LS1), and right monomer (LS2), with a color vertical bar indicating the local resolution range. Gold-standard FSC (GS-FSC) curves for each reconstruction are presented in the rightmost panel, with global resolution values marked at the 0.143 FSC cut-off. Source data are provided as a Source Data file.

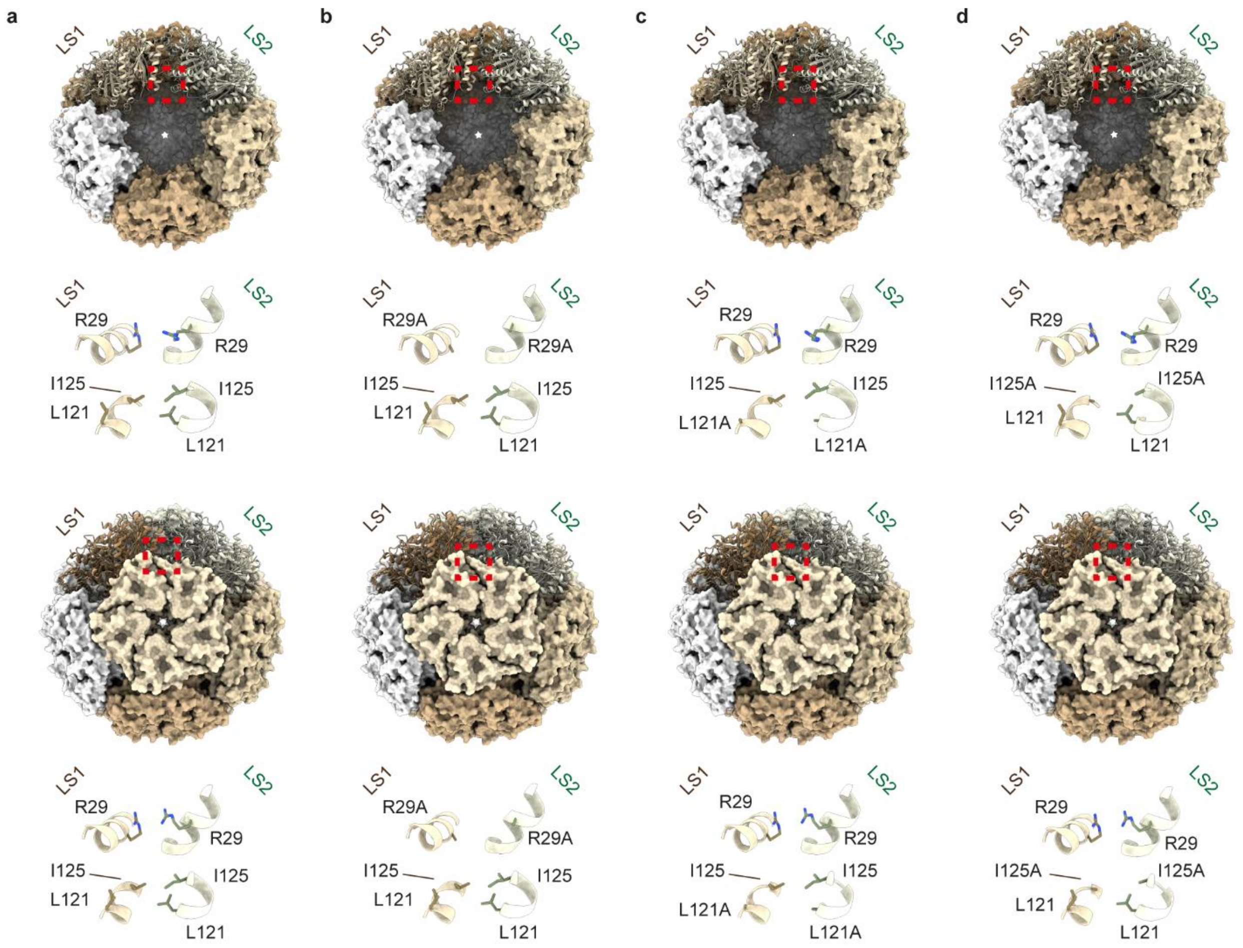

Supplementary Fig. 7: Structural comparison between wildtype and interface-mutant AaLS assemblies. a–d, Cryo-EM–derived models of the 11-pentamer AaLS cages (top panels) for wildtype (**a**, PDB ID: 9RYO) and the R29A (**b**, 9RYQ), I121A (**c**, 9RYV), and L125A (**d**, 9RYX) variants, shown in white–brown surface representation. The two AaLS protomers forming the pre-C3 interface are highlighted in ribbon form as LS1 (light brown, left) and LS2 (green, right). Red boxes indicate the interface regions enlarged immediately below, where the corresponding residues (R29, I121, L125) are shown in stick representation. Beneath these, the 12-pentamer cages for each variant (AaLS-wt, 9RYN; R29A, 9RYP; L121A, 9RYU; I125A, 9RYW) are shown using the same coloring scheme, followed by enlarged views of their interface residues. All variants preserve the wildtype cage architecture.

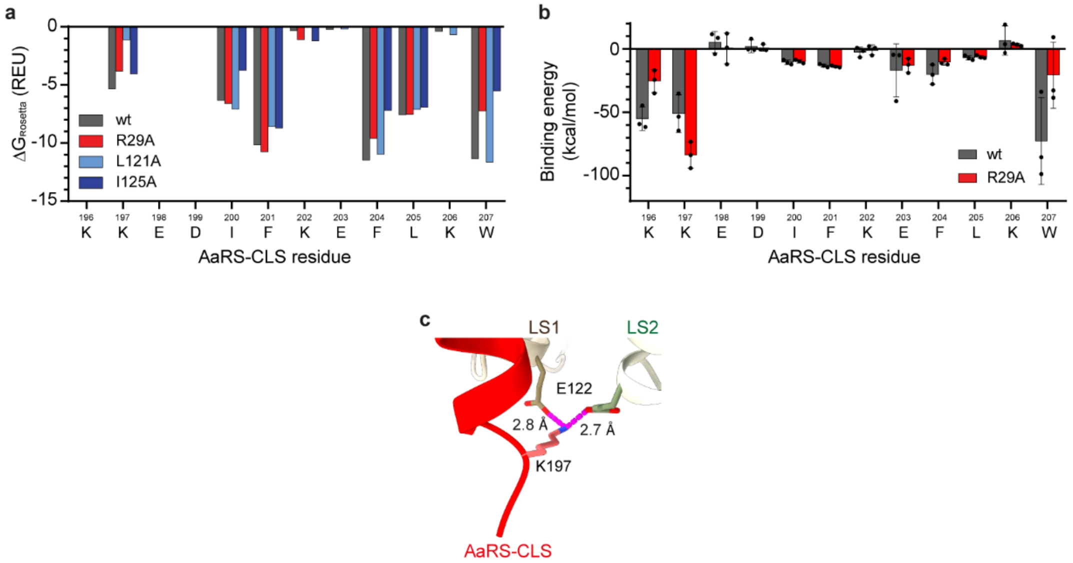

Supplementary Fig. 8: Computational simulation and energy calculation of AaRS-CLS binding. a, Per-residue binding energy contribution for AaRS-CLS in complex with AaLS, calculated using Rosetta Interface Analyzer ($\Delta G_{rosetta}$, in Rosetta Energy Units, REU). These values were used to derive the ΔΔG comparisons shown in Fig. 2g. **b,** Per-residue binding energy decomposition for AaRS-CLS in complex with AaLS, calculated from molecular dynamics (MD) simulation. These individual energy components sum to the total binding energy presented in Fig. 2j. The W207 breakdown here is identical to that shown in Fig. 2j. Data present mean ± standard deviations with individual data points from triplicate simulations. **c,** MD snapshot representing the electrostatic interactions between K197 of AaRS-CLS (red) and E122 residues from two AaLS protomers forming the pre-C3 interface (LS1, light brown; LS2, green). The side chains of these residues are displayed in stick representation, and the possible salt bridges are indicated by magenta dashed lines with measured interatomic distances labeled. Source data are provided as a Source Data file.

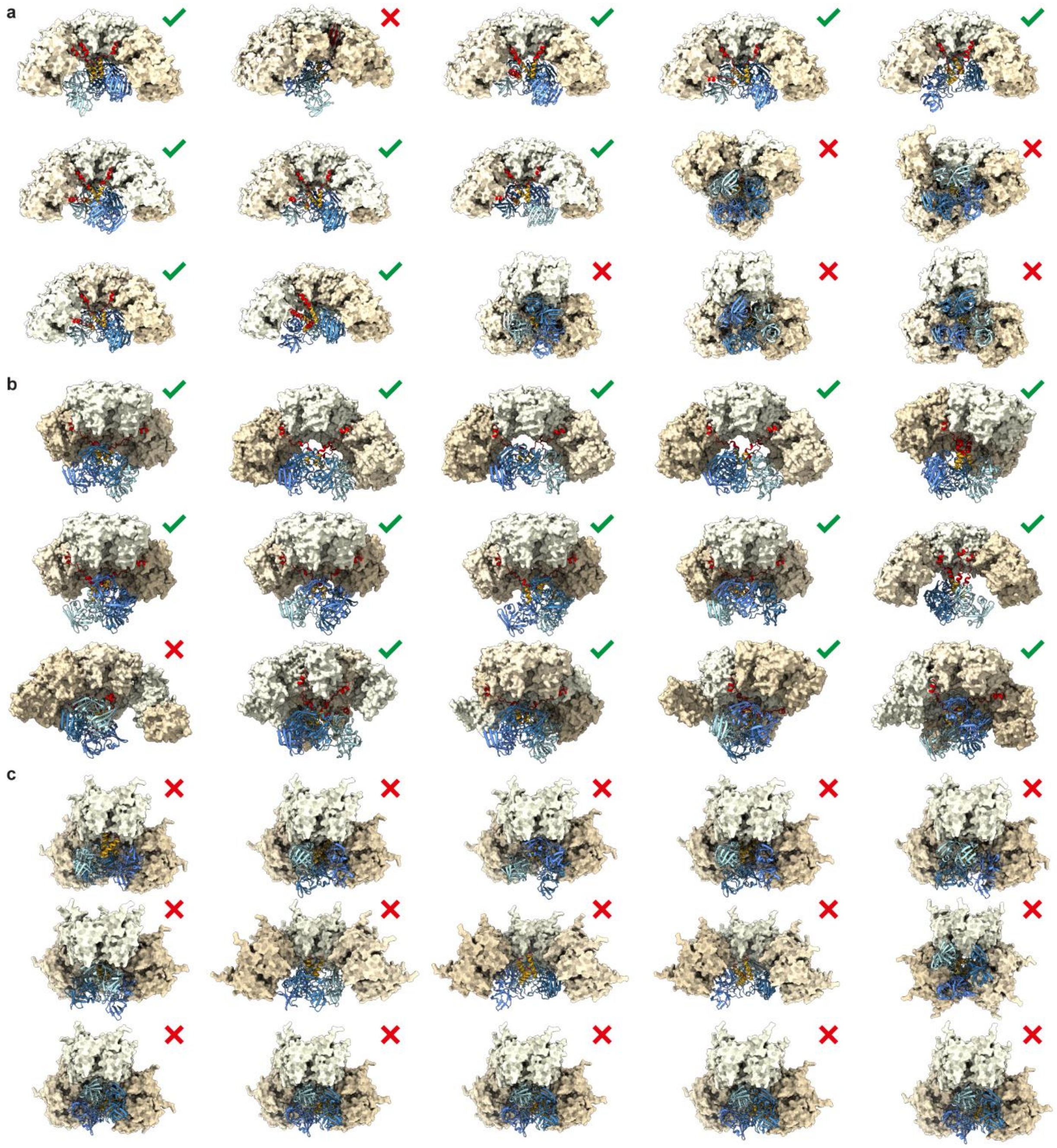

Supplementary Fig. 9: AlphaFold predictions of RS/LS complex formation for *Aquifex aeolicus*, *Bacillus subtilis,* and *Thermotoga maritima*. a–c, Complete sets of AlphaFold models generated for RS and LS from *A. aeolicus* (**a**), *B. subtilis* (**b**), and *T. maritima* (**c**). For each species, models were computed using 15 LS chains (3 pentamers) and 3 RS chains (a homotrimer), with 3 independent prediction runs using different random seeds (5 models per run; arranged as 5 × 3 panels). LS pentamers are shown as white–brown surfaces; RSs are shown in cartoon with domains colored distinctly: catalytic domain (blue), coiled-coil (orange), and CLS (red). Green tick marks indicate models in which RS and LS are predicted to form a complex via the canonical CLS–pre-C3 interface; red crosses indicate models lacking this interaction.

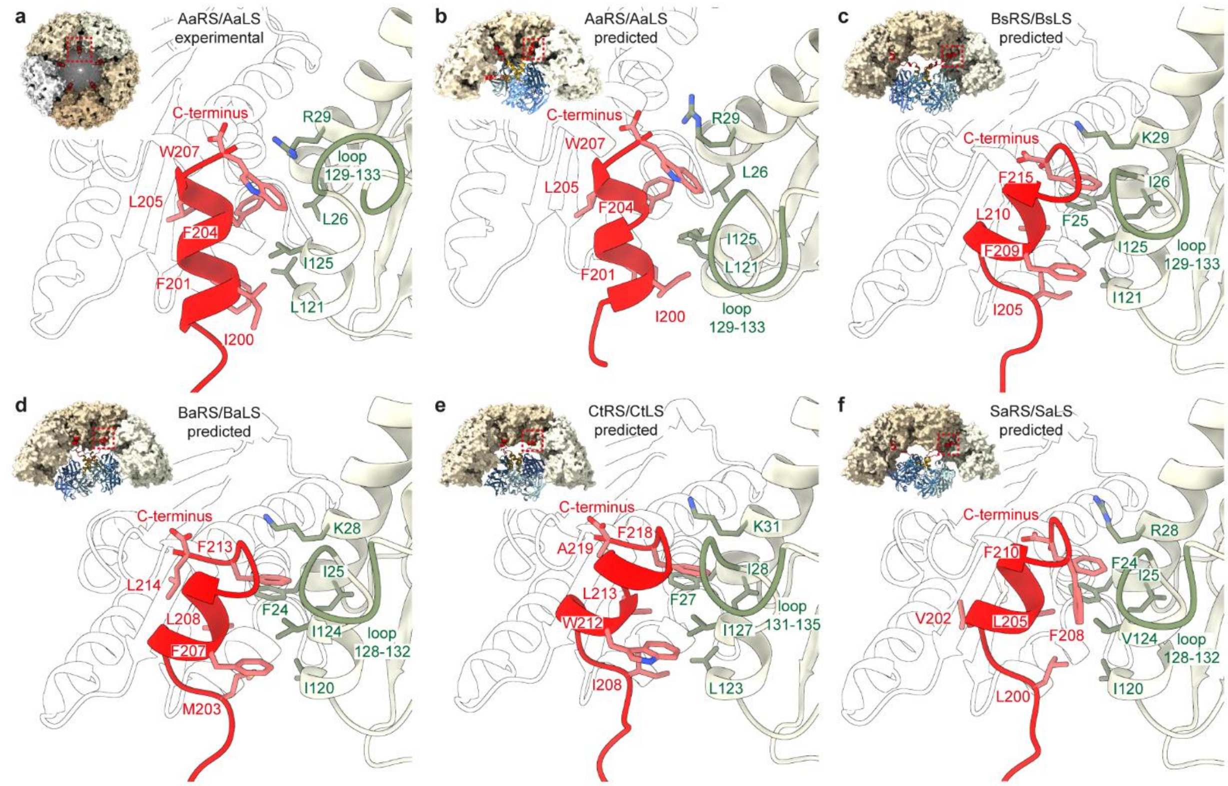

Supplementary Fig. 10: CLS–LS interactions in experimental and AlphaFold3-predicted complexes. a, C5-symmetrized cryo-EM structure of the *Aquifex aeolicus* riboflavin synthase/lumazine synthase (AaRS/AaLS) complex (PDB ID: 9RYJ; inset) showing CLS engagement at the pre-C3 interface (red box). CLS residues (red) and interacting residues from the LS protomer on the right (green) are shown as sticks, with the loop 129–133 highlighted. **b–f**, Representative AlphaFold3 models of RS/LS complexes at the corresponding pre-C3 site, predicted for variants from *Aquifex aeolicus* (AaRS/AaLS; **b**), *Bacillus subtilis* (BsRS/BsLS; **c**), *Bacillus anthracis* (BaRS/BaLS; **d**), *Chlorobaculum tepidum* (CtRS/CtLS; **e**), and *Staphylococcus aureus* (SaRS/SaLS; **f**). CLS and LS residues at positions structurally equivalent to those in AaRS/AaLS are shown.

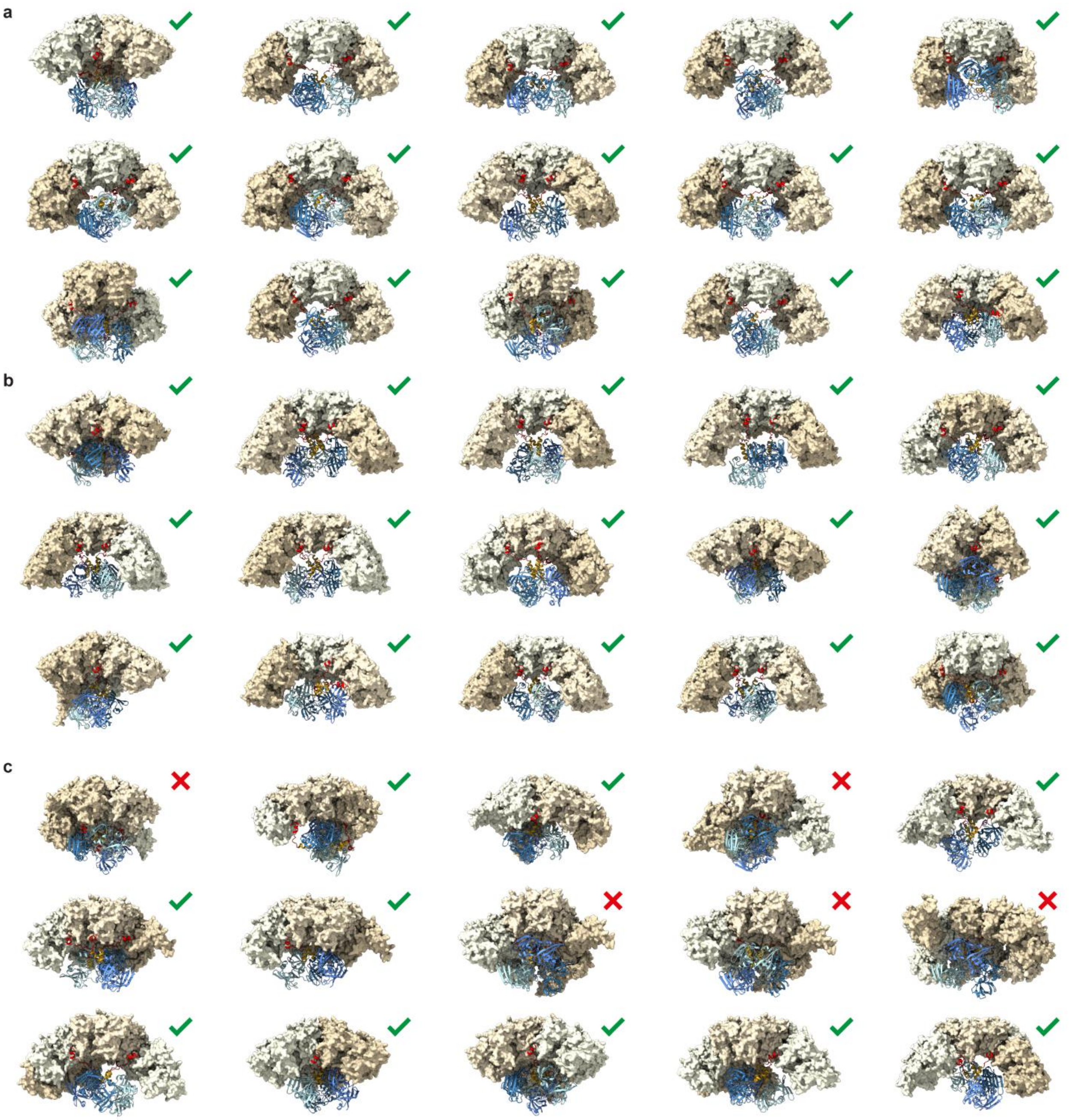

Supplementary Fig. 11: AlphaFold predictions of RS/LS complex formation for *Bacillus anthracis*, *Chlorobaculum tepidum*, and *Staphylococcus aureus*. a–c, Complete sets of AlphaFold models generated for RS and LS from *B. anthracis* (**a**), *C. tepidum* (**b**), and *S. aureus* (**c**). For each species, models were computed using 15 LS chains (3 pentamers) and 3 RS chains (a homotrimer), with 3 independent prediction runs using different random seeds (5 models per run; arranged as 5 × 3 panels). LS pentamers are shown as white–brown surfaces; RSs are shown in cartoon with domains colored distinctly: catalytic domain (blue), coiled-coil (yellow), and CLS (red). Green tick marks indicate models in which RS and LS are predicted to form a complex via the canonical CLS–pre-C3 interface; red crosses indicate models lacking this interaction.

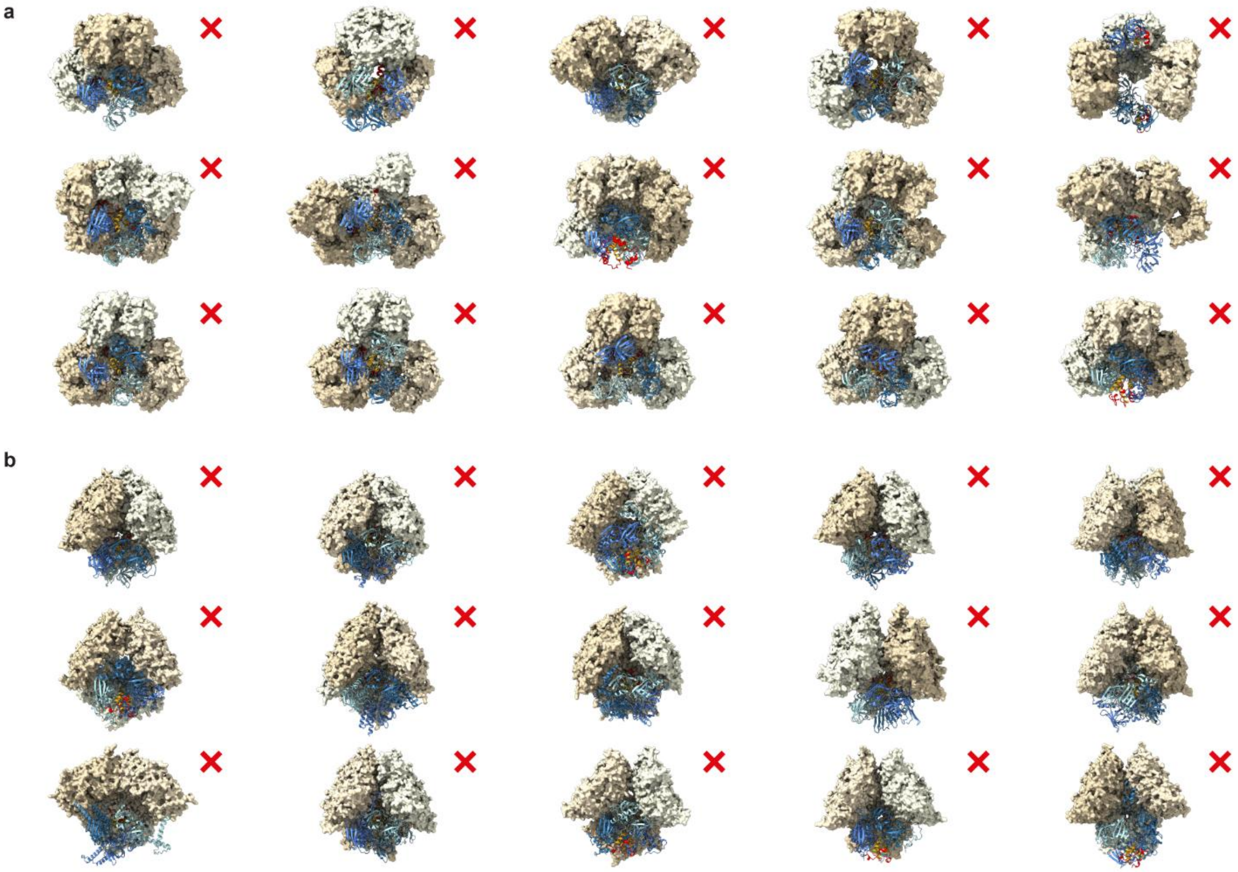

Supplementary Fig. 12: AlphaFold predictions of RS/LS complex formation for *Helicobacter pylori* and *Spinacia oleracea*. a–b, Complete sets of AlphaFold models generated for RS and LS from *H. pylori* (**a**), and *S. oleracea* (**b**). For each species, models were computed using 15 LS chains (3 pentamers) and 3 RS chains (a homotrimer), with 3 independent prediction runs using different random seeds (5 models per run; arranged as 5 × 3 panels). LS pentamers are shown as white–brown surfaces; RSs are shown in cartoon with domains coloured distinctly: catalytic domain (blue), coiled-coil (yellow), and CLS (red). Red crosses indicate that no models show formation of the canonical CLS–pre-C3 interaction.

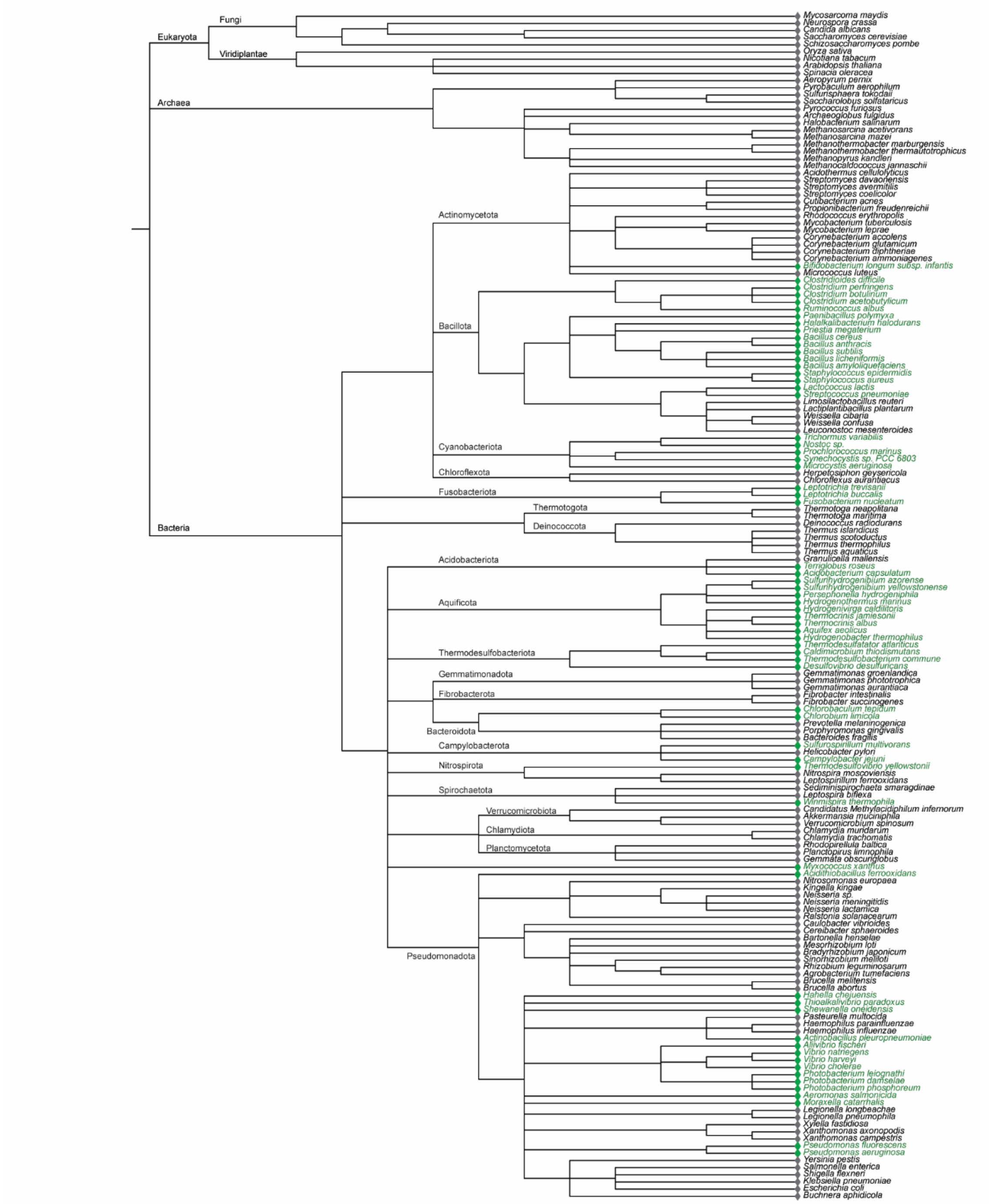

Supplementary Fig. 13: Taxonomy classification of the analyzed organisms. Species in which RS and LS are predicted to form a complex via the CLS-preC3 interface are highlighted in green. Organism names, phylum-level assignments, and domain-level groupings are indicated. A circular diagram of the same tree is presented in Fig. 4c.

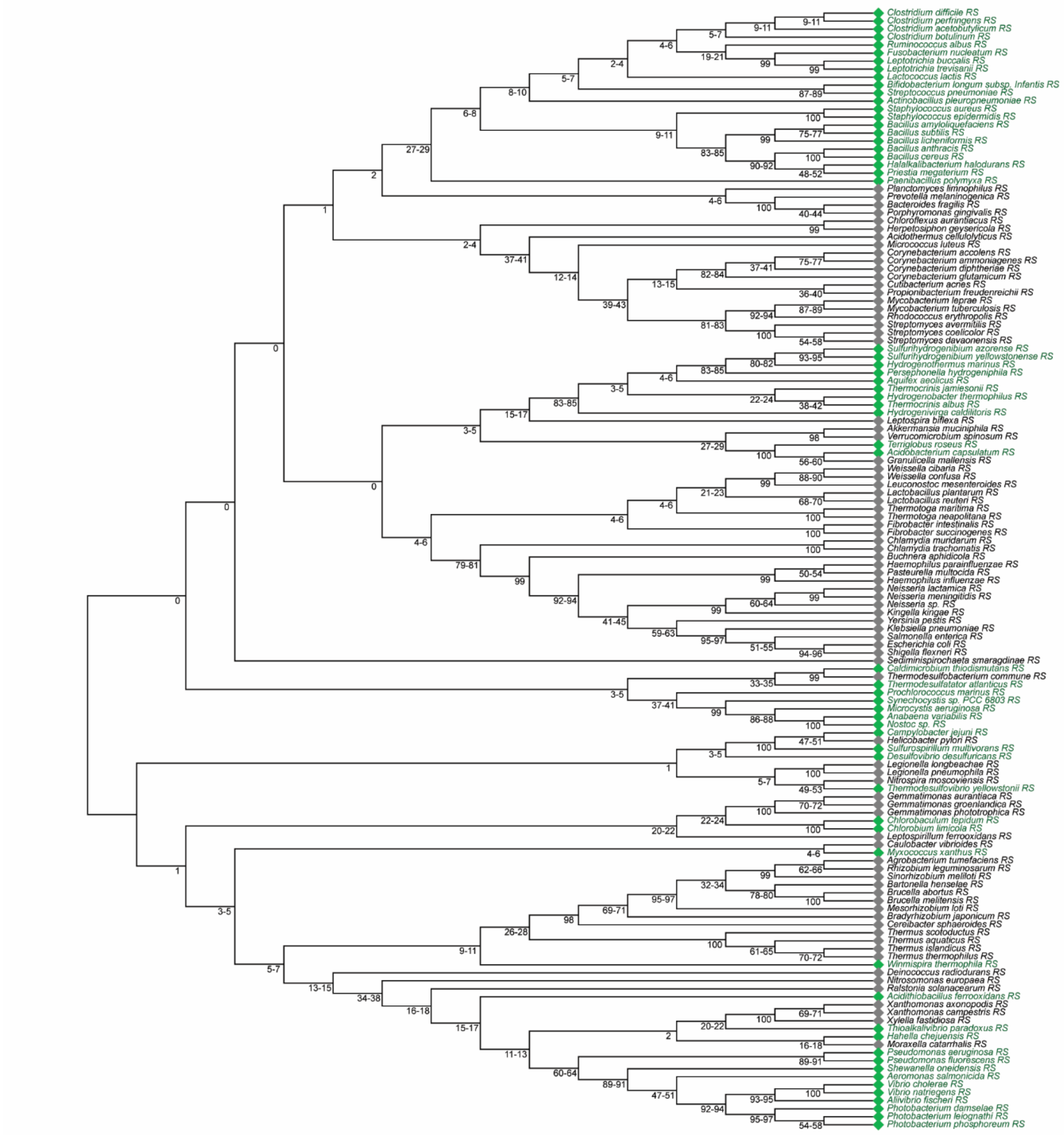

Supplementary Fig. 14: Phylogenetic analysis of RS from bacteria. Species in which RS and LS are predicted to form a complex via the CLS-preC3 interface are highlighted in green. The tree was constructed using the LG+G+I model with 1000 bootstrap replicates. Bootstrap values are shown at branch nodes.

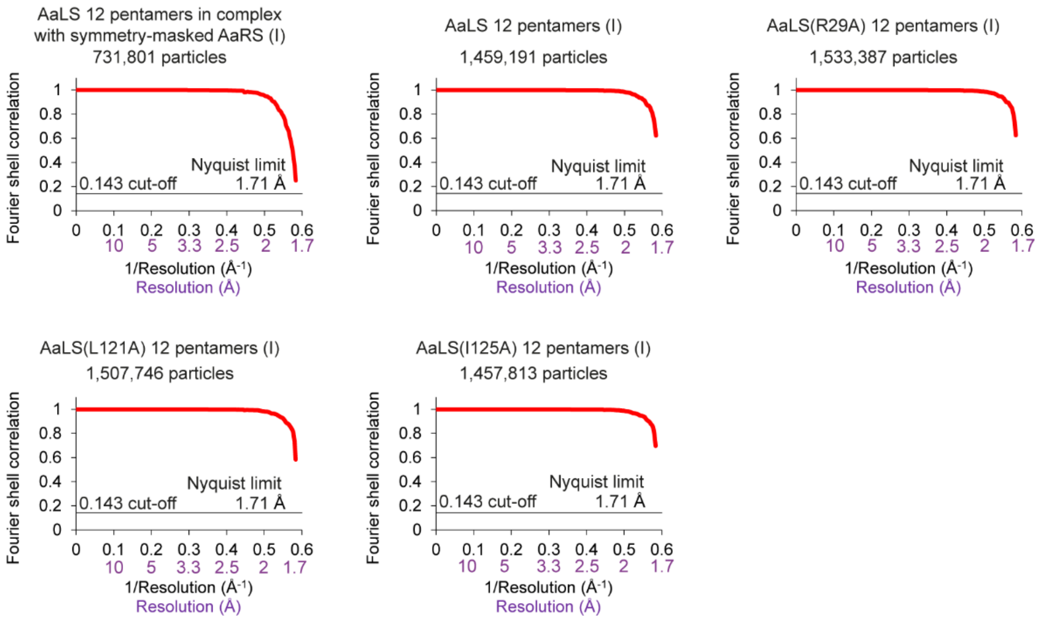

Supplementary Fig. 15: Global resolution estimation for cryo-EM reconstructions of icosahedral AaLS cages. Gold-standard Fourier shell correlation (GS-FSC) curves are shown for five reconstructions, with dataset names indicated above each curve. Due to the large number of particles (> 500,000) and the application of icosahedral (I) symmetry during each reconstruction, the curves approach the Nyquist frequency limit of 1.71 Å at the 0.143 FSC cut-off, beyond which further decline cannot be determined. Source data are provided as a Source Data file.

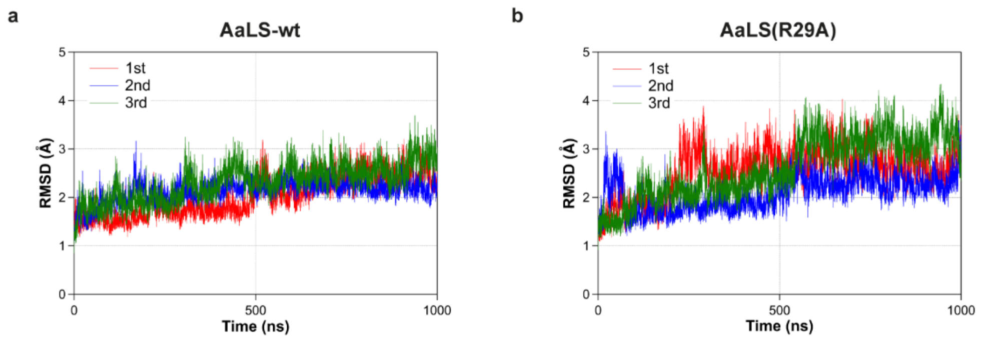

Supplementary Fig. 16: Time evolution of the root mean square deviation (RMSD) values in MD simulation. The values are calculated for main-chain atoms as relative to the initial structure. Each color indicates three independent simulation results obtained for wildtype AaLS (left, AaLS-wt) and R29A mutant (right, AaLS(R29A)). Source data are provided as a Source Data file.

s

Supplementary Table 1. Organisms analyzed in this study – Eukaryota and Archaea

Organisms	Domain	Phylum	NCBI RefSeq assembly	Genome assembly	Protein LS ID	Protein RS ID	RS/LS complex prediction[a]	Prediction score[b]
Aeropyrum pernix	Archaea	Crenarchaeota	GCF_000011125.1	ASM1112v1	WP_010866328.1	WP_373863049.1	N	0%
Archaeoglobus fulgidus	Archaea	Euryarchaeota	GCF_000008665.1	ASM866v1	WP_010879619.1	WP_010878913.1	N	0%
Halobacterium salinarum	Archaea	Euryarchaeota	GCF_004799605.1	ASM479960v1	WP_010902429.1	WP_168731194.1	N	0%
Methanocaldococcus jannaschii	Archaea	Euryarchaeota	GCF_000091665.1	ASM9166v1	WP_064496458.1	WP_010870697.1	N	0%
Methanopyrus kandleri	Archaea	Euryarchaeota	GCF_000007185.1	ASM718v1	WP_011018653.1	WP_011018731.1	N	0%
Methanosarcina acetivorans	Archaea	Euryarchaeota	GCF_000007345.1	ASM734v1	WP_011021820.1	WP_011021819.1	N	0%
Methanosarcina mazei	Archaea	Euryarchaeota	GCF_000970205.1	ASM97020v1	WP_011032198.1	WP_011032199.1	N	0%
Methanothermobacter marburgensis	Archaea	Euryarchaeota	GCF_000145295.1	ASM14529v1	WP_013296554.1	WP_048901271.1	N	0%
Methanothermobacter thermautotrophicus	Archaea	Euryarchaeota	GCF_027554905.1	ASM2755490v1	WP_010877002.1	WP_048061180.1	N	0%
Pyrobaculum aerophilum	Archaea	Crenarchaeota	GCF_000007225.1	ASM722v1	WP_011009286.1	WP_011008358.1	N	0%
Pyrococcus furiosus	Archaea	Euryarchaeota	GCF_008245085.1	ASM824508v1	WP_011011175.1	WP_011011173.1	N	0%
Sulfolobus solfataricus	Archaea	Crenarchaeota	GCF_003852155.1	ASM38521v1	WP_009988789.1	WP_009988788.1	N	0%
Sulfolobus tokodaii	Archaea	Crenarchaeota	GCF_000011205.1	ASM1120v1	WP_052846879.1	WP_010978357.1	N	0%
Candida albicans	Eukaryota	Ascomycota	GCF_000182965.3	ASM18296v3	XP_019330652.1	XP_721932.2	N	0%
Mycosarcoma maydis	Eukaryota	Basidiomycota	GCF_000328475.2	Umaydis521_2.0	XP_011389515.1	XP_011388024.1	N	0%
Neurospora crassa	Eukaryota	Ascomycota	GCF_000182925.2	NC12	XP_963689.1	XP_963907.1	N	0%
Saccharomyces cerevisiae	Eukaryota	Ascomycota	GCF_000146045.2	R64	NP_014498.1	NP_009815.1	N	0%
Schizosaccharomyces pombe	Eukaryota	Ascomycota	GCF_000002945.2	ASM294v3	NP_595463.1	NP_588312.1	N	0%
Arabidopsis thaliana (thale cress)	Eukaryota	Streptophyta	GCF_000001735.4	TAIR10.1	NP_563773.1	NP_565482.1	N	0%
Nicotiana tabacum	Eukaryota	Streptophyta	GCF_000715075.1	ASM71507v2	XP_016478266.1	XP_016474961.2	N	0%
Oryza sativa	Eukaryota	Streptophyta	GCF_034140825.1	AGIS1.0	XP_015637168.1	XP_015618419.1	N	0%
Spinacia oleracea	Eukaryota	Streptophyta	GCF_020520425.1	BTI_SOV_V1	NP_001413367.1	XP_021860852.2	N	0%

a, Y indicates that AlphaFold predicts RS/LS complex formation through the canonical CLS–pre-C3 interaction. N indicates that this canonical interaction is not predicted; cases marked N may reflect alternative interaction modes, weak or ambiguous association, or lack of detectable complex formation.

b, Prediction score reflects the percentage of AlphaFold models that support the canonical interaction. For each species, 15 models were generated (three runs × five models), and each supporting model contributed 1/15 to the score.

Supplementary Table 2. Organisms analyzed in this study – Bacteria

Organisms	Phylum	NCBI RefSeq assembly	Genome assembly	Protein LS	Protein RS	RS/LS complex prediction[a]	Prediction score[b]
Acidobacterium capsulatum	Acidobacteriota	GCF_000022565.1	ASM2256v1	WP_015897418.1	WP_015898444.1	Y	13%
Acidothermus cellulolyticus	Acidobacteriota	GCF_000015025.1	ASM1502v1	WP_011720106.1	WP_011720108.1	N	0%
Granulicella mallensis	Acidobacteriota	GCF_000178955.2	ASM17895v2	WP_014263797.1	WP_014264424.1	N	0%
Terriglobus roseus	Acidobacteriota	GCF_000265425.1	ASM26542v1	WP_014787223.1	WP_014786804.1	Y	20%
Bifidobacterium longum subsp. Infantis	Actinomycetota	GCF_900637215.1	49888_B01	WP_012576812.1	WP_012576810.1	Y	20%
Corynebacterium accolens	Actinomycetota	GCF_023520795.1	ASM2352079v1	WP_005279097.1	WP_005279094.1	N	0%
Corynebacterium ammoniagenes	Actinomycetota	GCF_001941425.1	ASM194142v1	WP_003845922.1	WP_003845926.1	N	0%
Corynebacterium diphtheriae	Actinomycetota	GCF_001457455.1	NCTC11397	WP_003851610.1	WP_014319118.1	N	0%
Corynebacterium glutamicum	Actinomycetota	GCF_000011325.1	ASM1132v1	WP_003856018.1	WP_011014470.1	N	0%
Cutibacterium acnes	Actinomycetota	GCF_006739385.1	ASM673938v1	WP_002516069.1	WP_002519613.1	N	0%
Micrococcus luteus	Actinomycetota	GCF_900475555.1	44257_B01	WP_010078768.1	WP_010078770.1	N	0%
Mycobacterium leprae	Actinomycetota	GCF_003253775.1	ASM325377v1	WP_010907798.1	WP_010907796.1	N	0%
Mycobacterium tuberculosis	Actinomycetota	GCF_000195955.2	ASM19595v2	NP_215932.2	NP_215928.1	N	0%
Propionibacterium freudenreichii	Actinomycetota	GCF_900087655.1	PFRJS14	WP_036943453.1	WP_013161982.1	N	0%
Rhodococcus erythropolis	Actinomycetota	GCF_000696675.2	ASM69667v2	WP_020907710.1	WP_003944892.1	N	0%
Streptomyces avermitilis	Actinomycetota	GCF_000009765.2	ASM976v2	WP_010988303.1	WP_010988300.1	N	0%
Streptomyces coelicolor	Actinomycetota	GCF_008931305.1	ASM893130v1	WP_003977385.1	WP_003977382.1	N	0%
Streptomyces davaonensis	Actinomycetota	GCF_000349325.1	ASM34932v1	WP_015661855.1	WP_015661852.1	N	0%
Aquifex aeolicus	Aquificota	GCF_000008625.1	ASM862v1	WP_010880027.1	WP_010881107.1	Y	60%
Hydrogenivirga caldilitoris	Aquificota	GCF_003664005.1	ASM366400v1	WP_121009344.1	WP_121009168.1	Y	20%
Hydrogenobacter thermophilus	Aquificota	GCF_000164905.1	ASM16490v1	WP_012963291.1	WP_012963018.1	Y	80%
Persephonella hydrogeniphila	Aquificota	GCF_900215515.1	IMG-taxon 2728369220	WP_097000102.1	WP_096999984.1	Y	73%
Sulfurihydrogenibium azorense	Aquificota	GCF_000021545.1	ASM2154v1	WP_012673659.1	WP_012674053.1	Y	20%
Sulfurihydrogenibium yellowstonense	Aquificota	GCF_000173615.1	ASM17361v1	WP_007547782.1	WP_007546525.1	Y	87%
Thermocrinis albus	Aquificota	GCF_000025605.1	ASM2560v1	WP_012992304.1	WP_012991678.1	Y	13%
Thermocrinis jamiesonii	Aquificota	GCF_000702425.1	ASM70242v1	WP_029551666.1	WP_029551322.1	Y	93%
Bacillus amyloliquefaciens	Bacillota	GCF_019396925.1	ASM1939692v1	WP_003153372.1	WP_007409425.1	Y	100%
Bacillus anthracis	Bacillota	GCF_000008445.1	ASM844v1	WP_000230891.1	WP_000493929.1	Y	100%
Bacillus cereus	Bacillota	GCF_046524075.1	ASM4652407v1	WP_000230895.1	WP_000493908.1	Y	100%
Bacillus licheniformis	Bacillota	GCF_034478925.1	ASM3447892v1	WP_003183115.1	WP_003183118.1	Y	93%
Bacillus subtilis	Bacillota	GCF_000009045.1	ASM904v1	NP_390206.1	NP_390208.1	Y	93%
Clostridium acetobutylicum	Bacillota	GCF_000218855.1	ASM21885v1	WP_010963913.1	WP_010963911.1	Y	93%
Clostridium botulinum	Bacillota	GCF_000063585.1	ASM6358v1	WP_003358088.1	WP_011987155.1	Y	93%
Clostridium difficile	Bacillota	GCF_018885085.1	ASM1888508v1	WP_004454467.1	WP_004454463.1	Y	100%
Clostridium perfringens	Bacillota	GCF_016027375.1	ASM1602737v1	WP_003471176.1	WP_003471205.1	Y	100%
Halalkalibacterium halodurans	Bacillota	GCF_005671335.1	ASM567133v1	WP_010897720.1	WP_010897718.1	Y	100%
Lactobacillus plantarum	Bacillota	GCF_009913655.1	ASM991365v1	WP_003640238.1	WP_076642475.1	N	0%
Lactobacillus reuteri	Bacillota	GCF_003703885.1	ASM370388v1	WP_122481266.1	WP_122481268.1	N	0%
Lactococcus lactis	Bacillota	GCF_003176835.1	ASM317683v1	WP_003130993.1	WP_103054691.1	Y	93%
Leuconostoc mesenteroides	Bacillota	GCF_000014445.1	ASM1444v1	WP_011679599.1	WP_011679597.1	N	0%
Paenibacillus polymyxa	Bacillota	GCF_022811565.1	ASM2281156v1	WP_017427524.1	WP_019687882.1	Y	100%
Priestia megaterium	Bacillota	GCF_006094495.1	ASM609449v1	WP_013056007.1	WP_013056005.1	Y	13%

Ruminococcus albus	Bacillota	GCF_000179635.2	ASM17963v2	WP_013498286.1	WP_013498284.1	Y	100%
Staphylococcus aureus	Bacillota	GCF_000013425.1	ASM1342v1	YP_500388.1	YP_500390.1	Y	67%
Staphylococcus epidermidis	Bacillota	GCF_006094375.1	ASM609437v1	WP_002456446.1	WP_002467848.1	Y	53%
Streptococcus pneumoniae	Bacillota	GCF_001457635.1	NCTC7465	WP_001099502.1	WP_000493842.1	Y	93%
Weissella cibaria	Bacillota	GCF_001951095.1	ASM195109v1	WP_010371275.1	WP_010371279.1	N	0%
Weissella confusa	Bacillota	GCF_004771075.1	ASM477107v1	WP_135388606.1	WP_135388610.1	N	0%
Bacteroides fragilis	Bacteroidota	GCF_000025985.1	ASM2598v1	WP_005785230.1	WP_005788568.1	N	0%
Chlorobaculum tepidum	Bacteroidota	GCF_000006985.1	ASM698v1	WP_010933692.1	WP_010932438.1	Y	100%
Chlorobium limicola	Bacteroidota	GCF_000020465.1	ASM2046v1	WP_012467224.1	WP_012465793.1	Y	100%
Porphyromonas gingivalis	Bacteroidota	GCF_000010505.1	ASM1050v1	WP_012457864.1	WP_012457761.1	N	0%
Prevotella melaninogenica	Bacteroidota	GCF_000144405.1	GCA_000144405.1	WP_004359555.1	WP_013265046.1	N	0%
Chlamydia muridarum	Chlamydiota	GCF_000006685.1	ASM668v1	WP_010229385.1	WP_010231218.1	N	0%
Chlamydia trachomatis	Chlamydiota	GCF_000008725.1	ASM872v1	NP_220251.1	NP_219915.1	N	0%
Chloroflexus aurantiacus	Chloroflexota	GCF_000018865.1	ASM1886v1	WP_012258065.1	WP_012258063.1	N	0%
Herpetosiphon geysericola	Chloroflexota	GCF_001306135.1	ASM130613v1	WP_054533245.1	WP_054537461.1	N	0%
Anabaena variabilis	Cyanobacteriota	GCF_009856605.1	ASM985660v1	WP_011318514.1	WP_011318240.1	Y	100%
Microcystis aeruginosa	Cyanobacteriota	GCF_001578075.1	ASM157807v1	WP_061432143.1	WP_061432315.1	Y	60%
Nostoc sp.	Cyanobacteriota	GCF_036919735.1	ASM3691973v1	WP_334923292.1	WP_334925493.1	Y	73%
Prochlorococcus marinus	Cyanobacteriota	GCF_000015665.1	ASM1566v1	WP_011821125.1	WP_011819821.1	Y	100%
Synechocystis sp. PCC 6803	Cyanobacteriota	GCF_000009725.1	ASM972v1	WP_010872197.1	WP_010873257.1	Y	53%
Deinococcus radiodurans	Deinococcota	GCF_020546685.1	ASM2054668v1	WP_010886802.1	WP_010886800.1	N	0%
Hydrogenothermus marinus	Deinococcota	GCF_003688665.1	ASM368866v1	WP_121922857.1	WP_121922783.1	Y	73%
Thermus aquaticus	Deinococcota	GCF_001399775.1	ASM139977v1	WP_003046858.1	WP_003048180.1	N	0%
Thermus islandicus	Deinococcota	GCF_000421625.1	ASM42162v1	WP_022799339.1	WP_022798788.1	N	0%
Thermus scotoductus	Deinococcota	GCF_000381045.1	ASM38104v1	WP_015716093.1	WP_015716822.1	N	0%
Thermus thermophilus	Deinococcota	GCF_000091545.1	ASM9154v1	WP_008634050.1	WP_011228413.1	N	0%
Fibrobacter intestinalis	Fibrobacterota	GCF_900167415.1	IMG-taxon 2582581325	WP_078777261.1	WP_078777266.1	N	0%
Fibrobacter succinogenes	Fibrobacterota	GCF_000146505.1	ASM14650v1	WP_014547328.1	WP_014547326.1	N	0%
Fusobacterium nucleatum	Fusobacteriota	GCF_003019295.1	ASM301929v1	WP_005902111.1	WP_011015638.1	Y	100%
Leptotrichia buccalis	Fusobacteriota	GCF_000023905.1	ASM2390v1	WP_015769773.1	WP_015769771.1	Y	100%
Leptotrichia trevisanii	Fusobacteriota	GCF_007990365.1	ASM799036v1	WP_155282940.1	WP_155282942.1	Y	100%
Gemmatimonas aurantiaca	Gemmatimonadota	GCF_000010305.1	ASM1030v1	WP_012682963.1	WP_012682961.1	N	0%
Gemmatimonas groenlandica	Gemmatimonadota	GCF_013004105.1	ASM1300410v1	WP_171224683.1	WP_171224684.1	N	0%
Gemmatimonas phototrophica	Gemmatimonadota	GCF_000695095.2	ASM69509v2	WP_026850552.1	WP_026850550.1	N	0%
Leptospirillum ferrooxidans	Nitrospirota	GCF_000284315.1	ASM28431v1	WP_014449319.1	WP_014449174.1	N	0%
Nitrospira moscoviensis	Nitrospirota	GCF_001273775.1	ASM127377v1	WP_083447791.1	WP_053380568.1	N	0%
Thermodesulfovibrio yellowstonii	Nitrospirota	GCF_000020985.1	ASM2098v1	WP_012545200.1	WP_012544953.1	Y	93%
Gemmata obscuriglobus	Planctomycetota	GCF_003149495.1	ASM314949v1	WP_010039459.1	WP_010044026.1	N	0%
Planctomyces limnophilus	Planctomycetota	GCF_000092105.1	ASM9210v1	WP_013111844.1	WP_230849309.1	N	0%
Rhodopirellula baltica	Planctomycetota	GCF_000196115.1	ASM19611v1	WP_011120220.1	WP_011123705.1	N	0%
Acidithiobacillus ferrooxidans	Pseudomonadota	GCF_000021485.1	ASM2148v1	WP_009568607.1	WP_009560911.1	Y	67%
Actinobacillus pleuropneumoniae	Pseudomonadota	GCF_003290385.1	ASM329038v1	WP_005596393.1	WP_005596391.1	Y	93%
Aeromonas salmonicida	Pseudomonadota	GCF_028355655.1	ASM2835565v1	WP_005317595.1	WP_058393748.1	Y	87%
Agrobacterium tumefaciens	Pseudomonadota	GCF_013318015.2	ASM1331801v2	WP_025593086.1	WP_003512512.1	N	0%
Aliivibrio fischeri	Pseudomonadota	GCF_000011805.1	ASM1180v1	WP_005418092.1	WP_011261426.1	Y	93%

Bartonella henselae	Pseudomonadota	GCF_019930925.1	ASM1993092v1	WP_011180660.1	WP_011180659.1	N	0%
Bradyrhizobium japonicum	Pseudomonadota	GCF_013752735.1	ASM1375273v1	WP_014494775.1	WP_014494774.1	N	0%
Brucella abortus	Pseudomonadota	GCF_000369945.1	Bruc_abor_544_V1	WP_002971482.1	WP_002969422.1	N	0%
Brucella melitensis	Pseudomonadota	GCF_000007125.1	ASM712v1	WP_004691907.1	WP_002969422.1	N	0%
Buchnera aphidicola	Pseudomonadota	GCF_000007365.1	ASM736v1	WP_011053953.1	WP_011053640.1	N	0%
Campylobacter jejuni	Pseudomonadota	GCF_000009085.1	ASM908v1	YP_002343820.1	YP_002344609.1	Y	13%
Caulobacter vibrioides	Pseudomonadota	GCF_000022005.1	ASM2200v1	YP_002516305.1	YP_002516303.1	N	0%
Cereibacter sphaeroides	Pseudomonadota	GCF_000012905.2	ASM1290v2	WP_002720942.1	WP_011338466.1	N	0%
Desulfovibrio desulfuricans	Pseudomonadota	GCF_017815575.1	ASM1781557v1	WP_022658445.1	WP_209817722.1	Y	93%
Escherichia coli	Pseudomonadota	GCF_000005845.2	ASM584v2	NP_414949.1	NP_416179.1	N	0%
Haemophilus influenzae	Pseudomonadota	GCF_020736045.1	ASM2073604v1	WP_011272561.1	WP_005657019.1	N	0%
Haemophilus parainfluenzae	Pseudomonadota	GCF_016127215.1	ASM1612721v1	WP_005695997.1	WP_032822576.1	N	0%
Hahella chejuensis	Pseudomonadota	GCF_000012985.1	ASM1298v1	WP_011399663.1	WP_011399665.1	Y	100%
Helicobacter pylori	Pseudomonadota	GCF_025998455.1	ASM2599845v1	WP_108520341.1	WP_267285914.1	N	0%
Kingella kingae	Pseudomonadota	GCF_900475905.1	49595_E01	WP_003786819.1	WP_003785701.1	N	0%
Klebsiella pneumoniae	Pseudomonadota	GCF_000240185.1	ASM24018v2	YP_005225407.1	YP_005227281.1	N	0%
Legionella longbeachae	Pseudomonadota	GCF_000091785.1	ASM9178v1	WP_003632017.1	WP_012978754.1	N	0%
Legionella pneumophila	Pseudomonadota	GCF_001941585.1	ASM194158v1	WP_010946914.1	WP_010946912.1	N	0%
Mesorhizobium loti	Pseudomonadota	GCF_003148495.1	ASM314849v1	WP_109668609.1	WP_109668607.1	N	0%
Moraxella catarrhalis	Pseudomonadota	GCF_002080125.1	ASM208012v1	WP_003662899.1	WP_003666743.1	Y	47%
Myxococcus xanthus	Pseudomonadota	GCF_000012685.1	ASM1268v1	WP_011554749.1	WP_011554750.1	Y	40%
Neisseria lactamica	Pseudomonadota	GCF_003351565.1	ASM335156v1	WP_003714718.1	WP_114935368.1	N	0%
Neisseria meningitidis	Pseudomonadota	GCF_022869645.1	ASM2286964v1	WP_002222781.1	WP_002234904.1	N	0%
Neisseria sp.	Pseudomonadota	GCF_030527965.1	ASM3052796v1	WP_303087249.1	WP_303086976.1	N	0%
Nitrosomonas europaea	Pseudomonadota	GCF_000009145.1	ASM914v1	WP_011113010.1	WP_011113008.1	N	0%
Pasteurella multocida	Pseudomonadota	GCF_002073255.2	ASM207325v2	WP_005716415.1	WP_005756710.1	N	0%
Photobacterium damselae	Pseudomonadota	GCF_038086725.1	ASM3808672v1	WP_005300681.1	WP_005300686.1	Y	100%
Photobacterium leiognathi	Pseudomonadota	GCF_048537465.1	Sc16.3	WP_008986352.1	WP_008986350.1	Y	100%
Photobacterium phosphoreum	Pseudomonadota	GCF_002954725.1	ASM295472v1	WP_036795804.1	WP_045031198.1	Y	87%
Pseudomonas aeruginosa	Pseudomonadota	GCF_000006765.1	ASM676v1	NP_252742.1	NP_252744.1	Y	53%
Pseudomonas fluorescens	Pseudomonadota	GCF_900215245.1	IMG-taxon 2617270901	WP_003194576.1	WP_053258235.1	Y	100%
Ralstonia solanacearum	Pseudomonadota	GCF_001587155.1	ASM158715v1	WP_003261773.1	WP_014617733.1	N	0%
Rhizobium leguminosarum	Pseudomonadota	GCF_004306555.1	ASM430655v1	WP_018241519.1	WP_028733636.1	N	0%
Salmonella enterica	Pseudomonadota	GCF_016028495.1	ASM1602849v1	WP_001021372.1	WP_000493971.1	N	0%
Shewanella oneidensis	Pseudomonadota	GCF_000146165.2	ASM14616v2	WP_011073313.1	WP_011073315.1	Y	100%
Shigella flexneri	Pseudomonadota	GCF_000006925.2	ASM692v2	NP_706303.2	NP_707562.1	N	0%
Sinorhizobium meliloti	Pseudomonadota	GCF_037023865.1	ASM3702386v1	WP_003529297.1	WP_003529304.1	N	0%
Sulfurospirillum multivorans	Pseudomonadota	GCF_000568815.1	ASM56881v1	WP_025345531.1	WP_025345597.1	Y	20%
Thioalkalivibrio paradoxus	Pseudomonadota	GCF_000227685.2	ASM22768v3	WP_025367507.1	WP_006748815.1	Y	27%
Vibrio cholerae	Pseudomonadota	GCF_008369605.1	ASM836960v1	WP_000864130.1	WP_000493874.1	Y	100%
Vibrio harveyi	Pseudomonadota	GCF_030060435.1	ASM3006043v1	WP_005440184.1	WP_283311559.1	Y	100%
Vibrio natriegens	Pseudomonadota	GCF_001456255.1	ASM145625v1	WP_014231012.1	WP_020333699.1	Y	100%
Xanthomonas axonopodis	Pseudomonadota	GCF_041519315.1	NCPPB972v1_ONT	WP_003490334.1	WP_057682145.1	N	0%
Xanthomonas campestris	Pseudomonadota	GCF_013388375.1	ASM1338837v1	WP_011035936.1	WP_011035934.1	N	0%
Xylella fastidiosa	Pseudomonadota	GCF_028891345.1	ASM2889134v1	WP_004086411.1	WP_004089677.1	N	0%

Yersinia pestis	Pseudomonadota	GCF_000222975.1	ASM22297v1	WP_002208666.1	WP_002210939.1	N	0%
Leptospira biflexa	Spirochaetota	GCF_000017685.1	ASM1768v1	WP_012389112.1	WP_012388393.1	N	0%
Sediminispirochaeta smaragdinae	Spirochaetota	GCF_000143985.1	ASM14398v1	WP_013256051.1	WP_013256053.1	N	0%
Winmispira thermophila	Spirochaetota	GCF_000184345.1	ASM18434v2	WP_014624148.1	WP_014624146.1	Y	80%
Caldimicrobium thiodismutans	Thermodesulfobacteriota	GCF_001548275.1	ASM154827v1	WP_068513597.1	WP_068515009.1	Y	100%
Thermodesulfatator atlanticus	Thermodesulfobacteriota	GCF_000421585.1	ASM42158v1	WP_022853385.1	WP_022852834.1	Y	100%
Thermodesulfobacterium commune	Thermodesulfobacteriota	GCF_000734015.1	ASM73401v1	WP_038060139.1	WP_038060609.1	Y	100%
Thermotoga maritima	Thermotogota	GCF_000230655.2	ASM23065v3	WP_004082371.1	WP_004082373.1	N	0%
Thermotoga neapolitana	Thermotogota	GCF_000018945.1	ASM1894v1	WP_015919250.1	WP_015919252.1	N	0%
Akkermansia muciniphila	Verrucomicrobiota	GCF_009731575.1	ASM973157v1	WP_102746922.1	WP_102744646.1	N	0%
Methylacidiphilum infernorum	Verrucomicrobiota	GCF_000019665.1	ASM1966v1	WP_012464000.1	WP_048810387.1	N	0%
Verrucomicrobium spinosum	Verrucomicrobiota	GCF_000172155.1	ASM17215v1	WP_009962131.1	WP_009964422.1	N	0%

a, Y indicates that AlphaFold predicts RS/LS complex formation through the canonical CLS–pre-C3 interaction. N indicates that this canonical interaction is not predicted; cases marked N may reflect alternative interaction modes, weak or ambiguous association, or lack of detectable complex formation.

b, Prediction score reflects the percentage of AlphaFold models that support the canonical interaction. For each species, 15 models were generated (three runs × five models), and each supporting model contributed 1/15 to the score.

Supplementary Table 3. Classification of bacteria based on riboflavin requirement and availability

Bacterium	RS/LS complex prediction[a]	Prediction score[b]	Requirement	Availability	Requirement Rationale	Availability Rationale
Acidithiobacillus ferrooxidans	Y	67%	Mod	Low	Chemolithoautotrophic ETC demand	Acid mine drainage; vitamin-poor extreme
Acidobacterium capsulatum	Y	13%	Mod	Low	Moderate chemoorganotroph	Acidic oligotrophic soils
Acidothermus cellulolyticus	N	0%	High	Low	Thermophilic cellulolysis; high flux	Acidic hot springs; low vitamins
Actinobacillus pleuropneumoniae	Y	93%	Mod	Mod	Standard respiratory pathogen	Porcine respiratory tract; moderate supply
Aeromonas salmonicida	Y	87%	Mod	Mod	Facultative pathogen; moderate flux	Aquatic host interface; moderate input
Agrobacterium tumefaciens	N	0%	Mod	High	Plant pathogen; moderate demand	Rhizosphere/plant wounds; vitamin-rich
Akkermansia muciniphila	N	0%	Mod	High	Mucin specialist; moderate metabolism	Human gut mucosa; vitamin-rich
Aliivibrio fischeri	Y	93%	High	High	Bioluminescence elevates flavin use	Light organ/coastal waters; rich supply
Anabaena variabilis	Y	100%	High	Mod	Photosynthesis + N_2 fixation	Freshwater mesotrophic habitats
Aquifex aeolicus	Y	60%	High	Low	Thermophilic chemolithotrophy	Vents/hot springs; vitamin-poor
Bacillus amyloliquefaciens	Y	100%	High	High	Fast growth; enzyme secretion	Rhizosphere soils; vitamin-rich
Bacillus anthracis	Y	100%	Mod	Mod	Typical pathogen metabolism	Soil reservoir; intermediate vitamins
Bacillus cereus	Y	100%	Mod	Mod	Opportunist; moderate respiration	Soil/decaying matter; intermediate
Bacillus licheniformis	Y	93%	High	Mod	High secretory/ETC activity	General soils; moderate vitamins
Bacillus subtilis	Y	93%	High	Mod	Fast growth; industrial metabolism	Common soils; moderate vitamins
Bacteroides fragilis	N	0%	Mod	High	Commensal anaerobe; moderate flux	Human colon; vitamin-rich
Bartonella henselae	N	0%	Mod	Mod	Facultative intracellular; moderate	Intracellular/blood niche; intermediate
Bifidobacterium longum subsp. Infantis	Y	20%	Mod	High	Fermentative commensal; moderate	Infant gut; vitamin-rich
Bradyrhizobium japonicum	N	0%	High	High	Nitrogen fixation; high electron flow	Root nodules/rhizosphere; rich
Brucella abortus	N	0%	Mod	Mod	Facultative intracellular; moderate	Intracellular macrophages; intermediate
Brucella melitensis	N	0%	Mod	Mod	Facultative intracellular; moderate	Intracellular macrophages; intermediate
Buchnera aphidicola	N	0%	Low	Mod	Genome-reduced endosymbiont	Aphid cells; host-supplied vitamins
Caldimicrobium thiodismutans	Y	100%	Mod	Low	Sulfur disproportionation; moderate flux	Thermal anoxic springs; low vitamins
Campylobacter jejuni	Y	13%	Mod	High	Microaerophilic pathogen; moderate	Animal gut; vitamin-rich
Caulobacter vibrioides	N	0%	Low	Low	Full pathways; slow oligotroph	Oligotrophic freshwater; low vitamins
Cereibacter sphaeroides	N	0%	High	Mod	Anoxygenic photosynthesis; high ETC	Freshwater/mud; moderate vitamins
Chlamydia muridarum	N	0%	Low	Mod	Obligate intracellular; minimal	Intracellular; partial host supply
Chlamydia trachomatis	N	0%	Low	Mod	Obligate intracellular; minimal	Intracellular; partial host supply
Chlorobaculum tepidum	Y	100%	High	Low	Anoxygenic photosynthesis	Anoxic hot springs; low vitamins
Chlorobium limicola	Y	100%	High	Low	Green sulfur photosynthesis	Anoxic sediments; low vitamins
Chloroflexus aurantiacus	N	0%	High	Low	Phototrophic thermophile; high ETC	Hot spring mats; vitamin-poor
Clostridium acetobutylicum	Y	93%	Mod	Mod	Fermentative solventogenesis	Soils/compost; intermediate vitamins
Clostridium botulinum	Y	93%	Mod	Mod	Anaerobe; moderate metabolism	Soils/sediments; intermediate
Clostridium difficile	Y	100%	Mod	High	Gut anaerobe; moderate flux	Human colon; vitamin-rich
Clostridium perfringens	Y	100%	High	High	Ultra-fast growth; high turnover	Gut/tissues; vitamin-rich
Corynebacterium accolens	N	0%	Low	Mod	Skin commensal; streamlined	Skin/mucosa; vitamin-replete
Corynebacterium ammoniagenes	N	0%	Mod	Mod	General heterotroph; moderate	Soil/water; intermediate
Corynebacterium diphtheriae	N	0%	Mod	Mod	Pathogen; typical demand	Upper respiratory mucosa; intermediate
Corynebacterium glutamicum	N	0%	Mod	Mod	Industrial heterotroph; moderate	Soil/vegetation; intermediate
Cutibacterium acnes	N	0%	Low	Mod	Slow aerotolerant fermenter	Sebaceous skin; host vitamins
Deinococcus radiodurans	N	0%	Mod	Low	Aerobic heterotroph; robust stress	Extremes/oligotrophic; low vitamins

Desulfovibrio desulfuricans	Y	93%	Mod	Low	Sulfate reduction; moderate yield	Anaerobic sediments; low vitamins
Escherichia coli	N	0%	High	High	Fast growth; versatile respiration	Mammalian gut; vitamin-rich
Fibrobacter intestinalis	N	0%	Mod	High	Gut fiber fermenter; moderate	Ruminant large intestine; rich
Fibrobacter succinogenes	N	0%	Mod	High	Cellulolytic gut symbiont	Rumen; vitamin-rich
Fusobacterium nucleatum	Y	100%	Mod	High	Anaerobic biofilm fermenter	Oral cavity; vitamin-rich
Gemmata obscuriglobus	N	0%	Low	Low	Very slow oligotroph	Oligotrophic lakes; low vitamins
Gemmatimonas aurantiaca	N	0%	Mod	Mod	Slow phototroph; moderate flux	Agricultural soils; intermediate
Gemmatimonas groenlandica	N	0%	Mod	Low	Cold-adapted phototroph; moderate	Arctic soils; low vitamins
Gemmatimonas phototrophica	N	0%	High	Mod	Aerobic anoxygenic phototrophy	Soil/water interfaces; moderate
Granulicella mallensis	N	0%	Low	Low	Very slow oligotroph	Arctic peat soils; low vitamins
Haemophilus influenzae	N	0%	Mod	Mod	Fastidious pathogen; moderate	Nasopharynx; intermediate
Haemophilus parainfluenzae	N	0%	Mod	Mod	Low-turnover commensal	Nasopharyngeal mucosa; intermediate
Hahella chejuensis	Y	100%	High	Mod	Secondary metabolite producer	Coastal marine biofilms; moderate
Halalkalibacterium halodurans	Y	100%	Mod	Low	Halophilic/alkaliphilic; moderate	Soda/alkaline habitats; low vitamins
Helicobacter pylori	N	0%	Mod	Low	Microaerophile; moderate flux	Stomach mucosa; low external vitamins
Herpetosiphon geysericola	N	0%	High	Low	Thermophilic filamentous heterotroph	Hot-spring mats; low vitamins
Hydrogenivirga caldilitoris	Y	20%	High	Low	Thermophilic H_2/S oxidizer	Vent systems; vitamin-poor
Hydrogenobacter thermophilus	Y	80%	High	Low	H_2-oxidizing thermophile	Hot springs; vitamin-poor
Hydrogenothermus marinus	Y	73%	Low	Low	SLOW thermophile; streamlined	Geothermal marine; low vitamins
Kingella kingae	N	0%	Low	Mod	Slow host-associated betaproteobacterium	Pediatric oropharynx; intermediate
Klebsiella pneumoniae	N	0%	Mod	High	Fast opportunist; moderate–fast flux	Gut/environment; vitamin-rich
Lactobacillus plantarum	N	0%	Low	High	Fast fermenter; low anabolic need	Fermented foods/gut; vitamin-rich
Lactobacillus reuteri	N	0%	Low	High	Small-genome mucosal fermenter	Intestinal/oral; vitamin-rich
Lactococcus lactis	Y	93%	Mod	High	Fermentative; moderate flux	Dairy/plant surfaces; rich supply
Legionella longbeachae	N	0%	Low	Mod	Slow intracellular parasite	Amoeba-associated; intermediate
Legionella pneumophila	N	0%	Mod	Mod	Facultative intracellular; moderate	Freshwater amoebae; intermediate
Leptospira biflexa	N	0%	Low	Mod	Free-living saprophyte; slow	Freshwater/soil; variable moderate
Leptospirillum ferrooxidans	N	0%	Mod	Low	Fe(II) chemolithotroph; moderate	Acid mine drainage; vitamin-poor
Leptotrichia buccalis	Y	100%	Mod	High	Fermentative; moderate redox	Oral biofilm; vitamin-rich
Leptotrichia trevisanii	Y	100%	Mod	High	Oral fermenter; moderate	Oral mucosa/biofilm; vitamin-rich
Leuconostoc mesenteroides	N	0%	Low	High	Lactic fermenter; limited biosynthesis	Fermented dairy; vitamin-rich
Mesorhizobium loti	N	0%	High	High	N_2 fixation; high electron flow	Root nodules; vitamin-rich
Methylacidiphilum infernorum	N	0%	High	Low	Obligate methanotroph; high ETC	Acidothermophilic soils; vitamin-poor
Micrococcus luteus	N	0%	Low	High	Skin/oral commensal; slow	Skin/oral niches; vitamin-rich
Microcystis aeruginosa	Y	60%	High	Mod	Cyanobacterial photosynthesis	Freshwater lakes; moderate nutrients
Moraxella catarrhalis	Y	47%	Low	Mod	Streamlined respiratory commensal	Upper airway; intermediate vitamins
Mycobacterium leprae	N	0%	Low	Mod	Obligate intracellular; ultra-slow	Intracellular tissues; partial supply
Mycobacterium tuberculosis	N	0%	Low	Mod	Very slow pathogen; reduced flux	Lung/macrophages; partial supply
Myxococcus xanthus	Y	40%	High	High	Predatory/secondary metabolites	Soils with high microbial activity
Neisseria lactamica	N	0%	Mod	Mod	Commensal; modest metabolism	Nasopharynx; intermediate vitamins
Neisseria meningitidis	N	0%	Mod	Mod	Pathogen; typical aerobic demand	Nasopharyngeal mucosa; intermediate
Neisseria sp.	N	0%	Mod	Mod	Similar to N. meningitidis	Human mucosa; intermediate
Nitrosomonas europaea	N	0%	Mod	Low	Ammonia oxidation; moderate flux	Low-organic water/soil; low vitamins
Nitrospira moscoviensis	N	0%	Low	Low	Nitrite oxidizer; slow growth	Oligotrophic biofilms; vitamin-poor
Nostoc sp.	Y	73%	High	Mod	Photosynthesis + N_2 fixation	Terrestrial/aquatic moist niches; moderate

Paenibacillus polymyxa	Y	100%	High	High	N_2 fixation + enzyme secretion	Rhizosphere-enriched soils; rich
Pasteurella multocida	N	0%	Mod	Mod	Facultative anaerobe; moderate	Animal respiratory tract; intermediate
Persephonella hydrogeniphila	Y	73%	Low	Low	Hyperthermophile; minimal anabolism	Deep-sea vents; ultra-low vitamins
Photobacterium damselae	Y	100%	High	High	Bioluminescent pathogen	Coastal nutrient-rich waters
Photobacterium leiognathi	Y	100%	High	Mod	Bioluminescent symbiont	Marine water/light organs; moderate
Photobacterium phosphoreum	Y	87%	High	Mod	Bioluminescent marine bacterium	Marine water/sediments; moderate
Planctomyces limnophilus	N	0%	Mod	Mod	Aquatic heterotroph; moderate	Freshwater lakes; intermediate
Porphyromonas gingivalis	N	0%	Mod	High	Periodontal anaerobe; moderate	Oral pockets; vitamin-rich
Prevotella melaninogenica	N	0%	Mod	High	Oral fermenter; moderate	Oral biofilms; vitamin-rich
Priestia megaterium	Y	13%	High	Mod	Large cell; fast growth	Soils/agricultural; moderate vitamins
Prochlorococcus marinus	Y	100%	High	Low	Oxygenic photosynthesis (ETC)	Oligotrophic open ocean; vitamin-poor
Propionibacterium freudenreichii	N	0%	Low	High	Slow anaerobic fermenter	Cheese/fermented dairy; rich
Pseudomonas aeruginosa	Y	53%	Mod	Mod	Versatile opportunist; moderate	Diverse habitats; intermediate
Pseudomonas fluorescens	Y	100%	High	Mod	Rapid aerobic respiration	Rhizosphere/soil; moderate
Ralstonia solanacearum	N	0%	Mod	High	Plant pathogen; moderate flux	Plant xylem; nutrient/vitamin flow
Rhizobium leguminosarum	N	0%	High	High	N_2 fixation; high electron flow	Rhizosphere/root nodules; rich
Rhodococcus erythropolis	N	0%	Mod	Mod	Versatile degrader; moderate	Soils incl. hydrocarbons; intermediate
Rhodopirellula baltica	N	0%	Mod	Mod	Aerobic heterotroph; moderate	Coastal waters/biofilms; intermediate
Ruminococcus albus	Y	100%	Mod	High	Cellulose degrader; moderate	Ruminant gut; vitamin-rich
Salmonella enterica	N	0%	Mod	High	Enteric pathogen; moderate	Animal GI tract; vitamin-rich
Sediminispirochaeta smaragdinae	N	0%	Mod	Low	Halophilic spirochete; moderate	Saline sediments; low vitamins
Shewanella oneidensis	Y	100%	High	Low	Extensive EET/respiration systems	Freshwater sediments; low free vitamins
Shigella flexneri	N	0%	Mod	High	Enteric pathogen; moderate	Human gut; vitamin-rich
Sinorhizobium meliloti	N	0%	High	High	N_2 fixation; high energy demand	Root nodules; vitamin-rich
Staphylococcus aureus	Y	67%	Mod	Mod	Opportunistic pathogen; moderate	Skin/nasal mucosa; intermediate
Staphylococcus epiderModis	Y	53%	Mod	Mod	Streamlined but not minimal	Skin surface; intermediate
Streptococcus pneumoniae	Y	93%	Mod	Mod	Fermentative pathogen; moderate	Nasopharynx; intermediate
Streptomyces avermitilis	N	0%	High	Mod	Antibiotic producer; high flux	Soils; moderate vitamins
Streptomyces coelicolor	N	0%	High	Mod	Secondary metabolism; high flux	Soils/decaying matter; moderate
Streptomyces davaonensis	N	0%	High	Mod	Secondary metabolites; high demand	Soil microenvironments; moderate
Sulfurihydrogenibium azorense	Y	20%	Low	Low	Sulfur oxidizer; simple network	Hot-spring outflows; low vitamins
Sulfurihydrogenibium yellowstonense	Y	87%	Low	Low	Thermoacidic sulfur oxidizer	Acidic hot springs; low vitamins
Sulfurospirillum multivorans	Y	20%	Mod	Low	Organohalide respiration; moderate	Anoxic niches/sludge; low vitamins
Synechocystis sp. PCC 6803	Y	53%	High	Mod	Oxygenic photosynthesis	Freshwater; moderate nutrients
Terriglobus roseus	Y	20%	Mod	Mod	Soil heterotroph; moderate	Mesic soils; intermediate vitamins
Thermocrinis albus	Y	13%	High	Low	Aerobic sulfur oxidizer; vigorous	Boiling sulfur springs; vitamin-poor
Thermocrinis jamiesonii	Y	93%	Low	Low	Slow sulfur chemolithotroph	Boiling sulfur springs; vitamin-poor
Thermodesulfatator atlanticus	Y	100%	Low	Low	Thermophilic sulfate reducer	Hydrothermal sediments; vitamin-poor
Thermodesulfobacterium commune	Y	100%	Low	Low	Sulfate reducer; slow growth	Sulfidic hot springs; vitamin-poor
Thermodesulfovibrio yellowstonii	Y	93%	Low	Low	Thermophilic sulfate reducer	Geothermal anoxic niches; vitamin-poor
Thermotoga maritima	N	0%	High	Low	Thermophilic fermenter; high turnover	Geothermal vents; vitamin-poor
Thermotoga neapolitana	N	0%	High	Low	Thermophilic high activity	Geothermal vents; vitamin-poor
Thermus aquaticus	N	0%	Low	Low	Slow thermophile; streamlined	Boiling springs; vitamin-poor
Thermus islandicus	N	0%	Low	Low	Slow thermophile; minimal	Hot-spring mats; vitamin-poor
Thermus scotoductus	N	0%	Low	Low	Thermophile; modest biosynthesis	Subsurface hot aquifers; oligotrophic

Thermus thermophilus	N	0%	High	Low	Thermophilic aerobe; strong ETC	Hot springs; vitamin-poor
Thioalkalivibrio paradoxus	Y	27%	High	Low	Sulfur-oxidizing chemolithotroph	Soda lakes; high pH/salinity; low vitamins
Verrucomicrobium spinosum	N	0%	Mod	Mod	Free-living heterotroph; moderate	Freshwater lake; mesotrophic
Vibrio cholerae	Y	100%	Mod	Mod	Fast enteropathogen; moderate	Brackish/estuaries; intermediate
Vibrio harveyi	Y	100%	High	High	Bioluminescent; high energy	Coastal seawater; vitamin-rich
Vibrio natriegens	Y	100%	High	High	Record-fast growth; high demand	Estuarine waters; vitamin-rich
Weissella cibaria	N	0%	Low	High	Lactic fermenter; reduced biosynthesis	Fermented foods/oral; vitamin-rich
Weissella confusa	N	0%	Low	High	Slow lactic fermenter	Fermented foods/mucosa; vitamin-rich
Winmispira thermophila	Y	80%	High	Low	Thermophilic spirochete; cofactor-heavy	Hydrothermal sediments; vitamin-poor
Xanthomonas axonopodis	N	0%	Mod	High	Plant pathogen; moderate energy	Leaf tissues; vitamin flow
Xanthomonas campestris	N	0%	Mod	High	Plant pathogen; xanthan production	Plant vascular tissues; vitamin flow
Xylella fastidiosa	N	0%	Low	High	Xylem-limited; very slow growth	Plant xylem; host vitamin flow
Yersinia pestis	N	0%	Mod	Mod	Facultative pathogen; moderate	Flea/rodent cycle; intermediate

a, Y indicates that AlphaFold predicts RS/LS complex formation through the canonical CLS–pre-C3 interaction. N indicates that this canonical interaction is not predicted; cases marked N may reflect alternative interaction modes, weak or ambiguous association, or lack of detectable complex formation.

b, Prediction score reflects the percentage of AlphaFold models that support the canonical interaction. For each species, 15 models were generated (three runs × five models), and each supporting model contributed 1/15 to the score.

Supplementary Table 4. Proteins experimentally characterized in this study

Name	Sequence	MW[a]	ε_{280}[a]
AaLS-wt (His)	MEIYEGKLTAEGLRFGIVASRFNHALVDRLVEGAIDCIVRHGGREEDITLVRVPGSWE<br>IPVAAGELARKEDIDAVIAIGVLIRGATPHFDYIASEVSKGLANLSLELRKPITFGVI<br>TADTLEQAIERAGTKHGNKGWEAALSAIEMANLFKSLRLEHHHHHH*	17,771	13,980
AaRS	MFTGLVEDLGKVKNLTLSSKGAKLSVETKLEDVKLGDSVSVNGACLTVVDIKSSTLTF<br>DVSPETLKRTNLGKLKTGDYVNLERALRVGERLGGHIVQGHVDFTAPVKSFNFLGEHY<br>ELVIEIPEEWSIYVVEKGSIALDGISLTVNYVKENKVFINIIPHTYKSTNLQFKKVGD<br>LLNVETDILGKYVINYLNKLKKKEDIFKEFLKW*	23,196	21,430
AaLS-wt (Strep)	MEIYEGKLTAEGLRFGIVASRFNHALVDRLVEGAIDCIVRHGGREEDITLVRVPGSWE<br>IPVAAGELARKEDIDAVIAIGVLIRGATPHFDYIASEVSKGLANLSLELRKPITFGVI<br>TADTLEQAIERAGTKHGNKGWEAALSAIEMANLFKSLRLEGGWSHPQFEK*	18,103	19,480
AaLS-L26A (Strep)	MEIYEGKLTAEGLRFGIVASRFNHAAVDRLVEGAIDCIVRHGGREEDITLVRVPGSWE<br>IPVAAGELARKEDIDAVIAIGVLIRGATPHFDYIASEVSKGLANLSLELRKPITFGVI<br>TADTLEQAIERAGTKHGNKGWEAALSAIEMANLFKSLRLEGGWSHPQFEK*	18,061	19,480
AaLS-R29A (Strep)	MEIYEGKLTAEGLRFGIVASRFNHALVDALVEGAIDCIVRHGGREEDITLVRVPGSWE<br>IPVAAGELARKEDIDAVIAIGVLIRGATPHFDYIASEVSKGLANLSLELRKPITFGVI<br>TADTLEQAIERAGTKHGNKGWEAALSAIEMANLFKSLRLEGGWSHPQFEK*	18,018	19,480
AaLS-E32A (Strep)	MEIYEGKLTAEGLRFGIVASRFNHALVDRLVAGAIDCIVRHGGREEDITLVRVPGSWE<br>IPVAAGELARKEDIDAVIAIGVLIRGATPHFDYIASEVSKGLANLSLELRKPITFGVI<br>TADTLEQAIERAGTKHGNKGWEAALSAIEMANLFKSLRLEGGWSHPQFEK*	18,045	19,480
AaLS-L121A (Strep)	MEIYEGKLTAEGLRFGIVASRFNHALVDRLVEGAIDCIVRHGGREEDITLVRVPGSWE<br>IPVAAGELARKEDIDAVIAIGVLIRGATPHFDYIASEVSKGLANLSLELRKPITFGVI<br>TADTAEQAIERAGTKHGNKGWEAALSAIEMANLFKSLRLEGGWSHPQFEK*	18,061	19,480
AaLS-E122A (Strep)	MEIYEGKLTAEGLRFGIVASRFNHALVDRLVEGAIDCIVRHGGREEDITLVRVPGSWE<br>IPVAAGELARKEDIDAVIAIGVLIRGATPHFDYIASEVSKGLANLSLELRKPITFGVI<br>TADTLAQAIERAGTKHGNKGWEAALSAIEMANLFKSLRLEGGWSHPQFEK*	18,045	19,480
AaLS-I125A (Strep)	MEIYEGKLTAEGLRFGIVASRFNHALVDRLVEGAIDCIVRHGGREEDITLVRVPGSWE<br>IPVAAGELARKEDIDAVIAIGVLIRGATPHFDYIASEVSKGLANLSLELRKPITFGVI<br>TADTLEQAAERAGTKHGNKGWEAALSAIEMANLFKSLRLEGGWSHPQFEK*	18,061	19,480
AaLS-H132A (Strep)	MEIYEGKLTAEGLRFGIVASRFNHALVDRLVEGAIDCIVRHGGREEDITLVRVPGSWE<br>IPVAAGELARKEDIDAVIAIGVLIRGATPHFDYIASEVSKGLANLSLELRKPITFGVI<br>TADTLEQAIERAGTKAGNKGWEAALSAIEMANLFKSLRLEGGWSHPQFEK*	18,037	19,480
GFP-CLS	MSKGEELFTGVVPILVELDGDVNGHKFSVRGEGEGDATNGKLTLKFICTTGKLPVPWP<br>TLVTTLTYGVQCFSRYPDHMKRHDFFKSAMPEGYVQERTISFKDDGTYKTRAEVKFEG<br>DTLVNRIELKGIDFKEDGNILGHKLEYNFNSHNVYITADKQKNGIKANFKIRHNVEDG<br>SVQLADHYQQNTPIGDGPVLLPDNHYLSTQSVLSKDPNEKRDHMVLLEFVTAAGITHG<br>MDELYKGSGKKEDIFKEFLKW*	28,575	-
BaLS	MVFEGHLVGTGLKVGVVVGRFNEFITSKLLGGALDGLKRHGVEENDIDVAWVPGAFEI<br>PLIAKKMANSGKYDAVITLGTVIRGATTHYDYVCNEVAKGVASLSLQTDIPVIFGVLT<br>TETIEQAIERAGTKAGNKGYESAVAAIEMAHLSKHWAGSGGWSHPQFEK*	17,553	22,460
BaRS	MFTGIVEELGTITNMQQSGEAMKLTIHANKILSDVHLGDSIAVNGICLTVTSFTTTSF<br>TVDAMPETMKSTSLRLLKSHSKVNLERAMAANGRFGGHFVSGHIDGIGTILNKKQHYN<br>AIYYKIAISDELLRYCLHKGSIAVDGTSLTIFDIDESSITISLIPHTVSESVIGEKNA<br>GDIVNIECDMIGKYIERFITKPVKRTGSMTENFLQENGFL*	23,421	7,450
CtLS	MQVQNIEGSLNASGLKFALVVSRFNDFIGQKLVEGAIDCIVRHGGSADEITVIRCPGA<br>FELPSVTRKAMLSGKYDAIVTLGVIIRGSTPHFDVIAAEATKGIAQVGMEAAIPVSFG<br>VLTTENLEQAIERAGTKAGNKGFDAALAAIEMANLYKQLGSGGWSHPQFEK*	17,606	8,480
CtRS	MFTGIVKDVGAIAASARQGSGMRLKVRYTSEAEFGDLAIDESVSINGACQTAVAVGPG<br>WFEVDTVAETLKKTTLGSFRPGTKVNLERAVRPMDRLGGHFVLGHVDGVGRVLRIEEV<br>GGSRMISVAFDSRFDAWIVSAGSIAIDGVSLTVASVEPGQFTVAIIPYTFGHTTITGL<br>AAGSEVNLEFDILGKYVARQHTAAAAPSQEPSRITESWLSGQGFA*	23,062	20,970
HpLS	MQIIEGKLQLQGNERVAILTSRFNHIITDRLQEGAMDCFKRHGGDEDLLDIVLVPGAY<br>ELPFILDKLLESEKYDGVCVLGAIIRGGTPHFDYVSAEATKGIAHAMLKYSMPVSFGV<br>LTTDNIEQAIERAGSKAGNKGFEAMSTLIELLSLCQTLKGGSGGWSHPQFEK*	18,341	11,460

HpRS	MFSGLIHQIAKVKSFHNNILSIESDLNPKLGDSIAVNGACLTAIESSKTHFNVELSQK<br>TQNSVALENYKDLVHIEPALKADASLDGHFVQGHIDAIGVIEKIIHSANQVDFFISVS<br>KETLLLCVEQGSIAVDGVSLTLSKVEEKGFWLTIIPYTLENTLFKTYKLKRRVNIETD<br>MLVRSVASILKKTKGFEKNFSWNDADALTLGY*	22,809	16,960
SaLS	MNFEGKLIGKDLKVAIVVSRFNDFITGRLLEGAKDTLIRHDVNEDNIDVAFVPGAFEI<br>PLVAKKLASSGNYDAVITLGCVIRGATSHYDYVCNEVAKGVSKVNDQTNVPVIFGILT<br>TESIEQAVERAGTKAGNKGAEAAVSAIEMANLLKSIKAGSGGWSHPQFEK*	17,694	9,970
SaRS	MFTGIVEEIGVVKSVQIRQSVRTIEIEAHKITADMHIGDSISVNGACLTVIDFNQTSF<br>TVQVIKGTENKTYLADVKRQSEVNLERAMSGNGRFGGHFVLGHVDELGTVSKINETAN<br>AKIITIQCSQHINKQLVKQGSITVDGVSLTVFDKHDNSFDIHLIPETRRSTILSSKKL<br>GDKVHLETDVLFKYVENILNKDKDQLSVDKLRAFGF*	23,313	2,980
TmLS	MKVVQGDYRGEGLKIAVVVPRFNDLVTSKLLEGALDGLKRHGVSDENITVVRIPGSME<br>AIYTLKRLLDLGVHDAIIVLGAVIRGETYHFNVVANEIGKAVAQFNMTSDIPIVFGVL<br>TTDTLEQALNRAGAKSGNKGFEAAMVAIEMANLRKRLRRDVFESDSNGRGSGGWSHPQ<br>FEK*	19,265	9,970
TmRS	MFTGIVQKVERGHIRGERIFFKRTWEVKLGESIAVNGVCLTVSGLSEEEYWFDVGEET<br>RRRTNLFVSRFYNLEKSLALGSRVEGHLVTGHVDGTVRFVGMERRGNSYFMFFSMPSE<br>RWAIVPKGSITLNGISLTVVETSLDTFSVQVIPHTFENTNLQYLVPGDPVNYEIDIIA<br>RYLKGVIDRGRTERGF*	21,678	25,440
SoLS	MVRELEGYVTKAQSFRFAIVVARFNEFVTRRLMEGALDTFKKYSVNEDIDVVWMVPGA<br>YELGVTAQALGKSGKYHAIVCLGAVVKGDTSHYDAVVNSASSGVLSAGLNSGVPCVFG<br>VLTCDNMDQAINRAGGKAGNKGAESALTAIEMASLFEHHLKAGSGGWSHPQFEK*	18,106	18,450
SoRS	MFTGIVEEIGRVKQMGYGEDGGFQLKVVGDIVLKDVNLGDSIAVNGTCLTVTEFDTKA<br>SEFTLGIAPETLRKTALMDLEPGSVVNLERALLPSTRMGGHFVQGHVDGTGEIVSLVE<br>EGDSLWVKIKTSPEILRYIVPKGFIAIDGTSLTVVDVFDQKLCFNIMLVAYTQQNVVI<br>PLKKVGQKVNLEVDILGKYVERLLSSSGVLDPTKFT*	22,806	11,460

a, calculated using Expasy ProtParam (https://web.expasy.org/protparam/)

Supplementary Table 5. Plasmids used in this study

name	gene	C-tag	promoter /operator	Ori	marker	ref
pMG_AaLS-wt-H	lumazine synthase from *Aquifix aeolicus* (AaLS)	6x His	P_{T7}/ *lac*O and P_{sal}	pBR322	Amp^R	22
pACYC_Ptet_ AaRS	riboflavin synthase from *Aquifix aeolicus* (AaRS)	-	P_{tet}/ *tet*O	p15A	Cm^R	22
pET_AaLS-wt-S	AaLS	Strep II	P_{T7}/ *lac*O	ColE1	Amp^R	this study
pET_AaLS-L26A-S	AaLS mutant L26A	Strep II	P_{T7}/ *lac*O	ColE1	Amp^R	this study
pET_AaLS-R29A-S	AaLS mutant R29A	Strep II	P_{T7}/ *lac*O	ColE1	Amp^R	this study
pET_AaLS-E32A-S	AaLS mutant E32A	Strep II	P_{T7}/ *lac*O	ColE1	Amp^R	this study
pET_AaLS-L121A-S	AaLS mutant L121A	Strep II	P_{T7}/ *lac*O	ColE1	Amp^R	this study
pET_AaLS-E122A-S	AaLS mutant E122A	Strep II	P_{T7}/ *lac*O	ColE1	Amp^R	this study
pET_AaLS-I125A-S	AaLS mutant I125A	Strep II	P_{T7}/ *lac*O	ColE1	Amp^R	this study
pET_AaLS-H132A-S	AaLS mutant H132A	Strep II	P_{T7}/ *lac*O	ColE1	Amp^R	this study
pACYC_Ptet_sfGFP-CLS	superfolder green fluorescent protein fused to AaRS-CLS	-	P_{tet}/ *tet*O	p15A	Cm^R	22
pET28_CtLS-S	lumazine synthase from *Chlorobaculum tepidum* (CtLS)	Strep II	P_{T7}/ *lac*O	ColE1	Kan^R	this study
pACYC_Ptet_ CtRS	riboflavin synthase from *Chlorobaculum tepidum* (CtRS)	-	P_{tet}/ *tet*O	p15A	Cm^R	this study
pET28_SaLS-S	lumazine synthase from *Staphylococcus aureus* (SaLS)	Strep II	P_{T7}/ *lac*O	ColE1	Kan^R	this study
pACYC_Ptet_ SaRS	riboflavin synthase from *Staphylococcus aureus* (SaRS)	-	P_{tet}/ *tet*O	p15A	Cm^R	this study
pET28_HpLS-S	lumazine synthase from *Helicobacter pylori* (HpLS)	Strep II	P_{T7}/ *lac*O	ColE1	Kan^R	this study
pACYC_Ptet_ HpRS	riboflavin synthase from *Helicobacter pylori* (HpRS)	-	P_{tet}/ *tet*O	p15A	Cm^R	this study
pET28_TmLS-S	lumazine synthase from *Thermotoga maritima* (TmLS)	Strep II	P_{T7}/ *lac*O	ColE1	Kan^R	this study
pACYC_Ptet_ TmRS	riboflavin synthase from *Thermotoga maritima* (TmRS)	-	P_{tet}/ *tet*O	p15A	Cm^R	this study
pET28_SoLS-S	lumazine synthase from *Spinacia oleracea* (SoLS)	Strep II	P_{T7}/ *lac*O	ColE1	Kan^R	this study
pACYC_Ptet_ SoRS	riboflavin synthase from *Spinacia oleracea* (SoRS)	-	P_{tet}/ *tet*O	p15A	Cm^R	this study

a. P_{T7}/*lacO,* T7 promoter combined with lactose operator and the LacI repressor (*lacI*); P_{sal}, the salicylate promoter regulated by the NahR transcriptional activator; P_{tet}/*tetO*, the tetracycline promoter combined with tetracycline operator

b. Amp^R, β-lactamase; Cm^R, chloramphenicol acetyltransferase; Kan^R, aminoglycoside 3'-phosphotransferase

Supplementary Table 6. Oligonucleotides used in this study

Name	Sequence
FW_AaLS_L26A	**TCACGTTTTAATCATGCTgcaGTCGACCGTCTGGTGGAGG**
RV_AaLS_L26A	**CCTCCACCAGACGGTCGACtgcAGCATGATTAAAACGTGA**
FW_AaLS_R29A	**CACGTTTTAATCATGCTCTTGTCGACgcgCTGGTGGAGGGTGCAATTGATTGC**
RV_AaLS_R29A	**GCAATCAATTGCACCCTCCACCAGcgcGTCGACAAGAGCATGATTAAAACGTG**
FW_AaLS_E32A	**GTCGACCGTCTGGTGGcaGGTGCAATTGATTGCATA**
RV_AaLS_E32A	**TATGCAATCAATTGCACCtgCCACCAGACGGTCGAC**
FW_AaLS_L121A	**ATTACAGCTGACACCgcaGAACAGGCTATCGAGCGCGC**
RV_AaLS_L121A	**GCGCGCTCGATAGCCTGTTCtgcGGTGTCAGCTGTAAT**
FW_AaLS_E122A	**ATTACAGCTGACACCTTGGcACAGGCTATCGAGCGCGC**
RV_AaLS_E122A	**GCGCGCTCGATAGCCTGTgCCAAGGTGTCAGCTGTAAT**
FW_AaLS_I125A	**TGACACCTTGGAACAGGCTgcaGAGCGCGCCGGCACAAAA**
RV_AaLS_I125A	**TTTTGTGCCGGCGCGCTCtgcAGCCTGTTCCAAGGTGTCA**
FW_AaLS_H132A	**GAGCGCGCCGGCACAAAAgcaGGCAACAAAGGTTGGGA**
RV_AaLS_H132A	**TCCCAACCTTTGTTGCCtgcTTTTGTGCCGGCGCGCTC**

Supplementary Table 7. Cryo-EM data collection and structure refinement statistics for the AaLS-RS inclusion complex

**Assembly**	12 pentamers<br>I-symmetry-masked AaRS	11 pentamers<br>C5-symmetrized<br>C-terminus of AaRS	11 pentamers<br>AaRS trimer	10 pentamers<br>AaRS trimer
**EMPIAR**	13016			
**EMDB**	54381	54382	54383	54385
**PDB**	9RYI	9RYJ	9RYK	9RYM
Magnification	105,000 ×			
Voltage (keV)	300			
Electron exposure ($e^{-}/Å^{2}$)	40			
Defocus range	-0.6 to -2.1			
Pixel size (Å)	0.8456			
Micrographs (no.)	8,625			
Initial particles (no.)	1,749,455			
Final particles (no.)	731,801	116,134	25,459	17,809
Symmetry imposed	I	C5	C1	C1
Map resolution (Å)	1.71[1]	1.99	2.60	2.78
FSC threshold	0.143	0.143	0.143	0.143
Map resolution range<br>25th–75th percentile (Å)	1.71[1]	1.89–1.97	2.3–3.0	2.5–3.1
Map sharpening B-Factor ($Å^{2}$)	54.9	49.0	48.4	50.1
**Model building**				
Initial Model	AaLS PDB ID: 1HQK<br>AaRS: AlphaFold 3 prediction			
Model composition				
Protein chains	60	60	58	53
Protein residues	9,240	8,530	9,091	8,321
Non-hydrogen atoms	73,110	65,310	69,649	63,764
Waters	2,190	0	0	0
Ligands (PO_4)	60	0	0	0
Protein *B* factors (mean $Å^{2}$)	41.13	57.96	73.65	75.82
R.M.S. deviations				
Bond lengths (Å)	0.003	0.005	0.005	0.004
Bond angles (º)	0.894	0.951	0.917	0.895
Validation				
MolProbity score	0.83	0.91	0.99	0.87
Clash score	1.18	0.80	1.44	1.11
Poor rotamers (%)	0.00	0.00	0.00	0.00
Ramachandran				
Favored (%)	98.03	97.15	97.43	97.76
Allowed (%)	1.97	2.85	2.57	2.24
Outliers (%)	0.00	0.00	0.00	0.00
CC (volume)	0.95	0.94	0.87	0.84

[1] Nyquist limit

Supplementary Table 8. Cryo-EM data collection and structure refinement statistics for empty AaLS cages

Variant	AaLS		AaLS (R29A)		AaLS (L121A)		AaLS (I125A)	
Assembly	12 pentamers	11 pentamers	12 pentamers	11 pentamers	12 pentamers	11 pentamers	12 pentamers	11 pentamers
EMPIAR	13017		13018		13019		13020	
EMDB	54386	54387	54388	54389	54392	54393	54394	54395
PDB	9RYN	9RYO	9RYP	9RYQ	9RYU	9RYV	9RYW	9RYX
**Data collection and processing**								
Magnification	105,000 ×		105,000 ×		105,000 ×		105,000 ×	
Voltage (keV)	300		300		300		300	
Electron exposure (e^-/Å^2)	40		40		40		40	
Defocus range	-0.6 to -2.1		-0.6 to -2.1		-0.6 to -2.1		-0.6 to -2.1	
Pixel size (Å)	0.8456		0.8456		0.8456		0.8456	
Micrographs (no.)	4,998		5,306		4,942		5,159	
Initial particle images (no.)	1,812,894		1,760,989		1,714,146		1,608,808	
Final particle images (no.)	1,459,191	36,125	1,533,387	32,433	1,507,746	9,926	1,457,813	10,485
Symmetry imposed	I	C5	I	C5	I	C5	I	C5
Map resolution (Å)	1.71[1]	2.09	1.71[1]	2.08	1.71[1]	2.49	1.71[1]	2.31
FSC threshold	0.143	0.143	0.143	0.143	0.143	0.143	0.143	0.143
Map resolution range 25th–75th percentile (Å)	1.71[1]	1.9–2.2	1.71[1]	1.9–2.2	1.71[1]	2.3–2.6	1.71[1]	2.1–2.5
Map sharpening B-Factor (Å^2)	52.3	46.3	51.8	43.9	51.4	47.2	51.5	44.5
**Model building**								
Initial model	AaLS PDB ID: 1HQK							
Model composition								
Protein chains	60	55	60	55	60	55	60	55
Protein residues	9,240	8,470	9,240	8,470	9,240	8,470	9,240	8,470
Non-hydrogen atoms	73,110	64,735	72,750	64,405	72,930	64,570	72,810	64,570
Waters	2,190	0	2,190	0	2,190	0	2,070	0
Ligands (PO_4)	60	0	60	0	60	0	60	0
Protein *B* factors (mean Å^2)	39.24	65.89	38.03	64.47	38.38	81.16	38.58	73.44
R.M.S. deviations								
Bond lengths (Å)	0.003	0.004	0.004	0.006	0.004	0.007	0.005	0.004
Bond angles (º)	0.909	0.897	0.913	0.948	0.929	0.958	0.941	0.896
Validation								
MolProbity score	0.97	0.78	0.91	0.95	0.92	0.99	0.88	0.87
Clash score	1.24	0.82	0.97	1.24	0.68	1.34	1.44	1.17
Poor rotamers (%)	0.00	0.00	0.00	0.00	0.00	0.00	0.00	0.00
Ramachandran								
Favored (%)	97.37	97.91	97.37	97.49	96.80	97.37	98.03	97.83
Allowed (%)	2.63	2.09	2.63	2.51	3.20	2.63	1.97	2.17
Outliers (%)	0.00	0.00	0.00	0.00	0.00	0.00	0.00	0.00
CC (volume)	0.95	0.93	0.95	0.94	0.95	0.92	0.95	0.91

[1] Nyquist limit
