# Supplementary material for: A molecular basis for stoichiometric enzyme encapsulation in the vitamin B2 biosynthesis compartment
Source: Nat Commun. 2026 May 16;17:6498. doi: 10.1038/s41467-026-73260-4 (PMC13376624; doi:10.1038/s41467-026-73260-4)
Supplement: Supplementary file 2 — Description of Additional Supplementary Files [file 41467_2026_73260_MOESM2_ESM.pdf]

Description of Additional Supplementary Files

File Name: Supplementary Movie 1
Description: Molecular dynamics simulation of AaRS-CLS binding to the pre-C3 interface formed by two AaLS-wt pentamers. The movie shows 100 frames sampled every 10 ns from a 1.0 µs simulation, capturing dynamic electrostatic interactions at the binding interface, with R29–C-terminus and E122–K197 interactions highlighted.
