# Supplementary material for: A molecular basis for stoichiometric enzyme encapsulation in the vitamin B2 biosynthesis compartment
Source: Nat Commun. 2026 May 16;17:6498. doi: 10.1038/s41467-026-73260-4 (PMC13376624; doi:10.1038/s41467-026-73260-4)
Supplement: Supplementary file 4 — Reporting Summary [file 41467_2026_73260_MOESM4_ESM.pdf]

nature portfolio

Corresponding author(s): Yusuke Azuma

Last updated by author(s): Apr 19, 2026

Reporting Summary

Nature Portfolio wishes to improve the reproducibility of the work that we publish. This form provides structure for consistency and transparency in reporting. For further information on Nature Portfolio policies, see our Editorial Policies and the Editorial Policy Checklist.

Statistics

For all statistical analyses, confirm that the following items are present in the figure legend, table legend, main text, or Methods section.

n/a	Confirmed	
☐	☒	The exact sample size (*n*) for each experimental group/condition, given as a discrete number and unit of measurement
☐	☒	A statement on whether measurements were taken from distinct samples or whether the same sample was measured repeatedly
☐	☒	The statistical test(s) used AND whether they are one- or two-sided<br>*Only common tests should be described solely by name; describe more complex techniques in the Methods section.*
☒	☐	A description of all covariates tested
☐	☒	A description of any assumptions or corrections, such as tests of normality and adjustment for multiple comparisons
☐	☒	A full description of the statistical parameters including central tendency (e.g. means) or other basic estimates (e.g. regression coefficient) AND variation (e.g. standard deviation) or associated estimates of uncertainty (e.g. confidence intervals)
☐	☒	For null hypothesis testing, the test statistic (e.g. *F*, *t*, *r*) with confidence intervals, effect sizes, degrees of freedom and *P* value noted<br>*Give P values as exact values whenever suitable.*
☒	☐	For Bayesian analysis, information on the choice of priors and Markov chain Monte Carlo settings
☒	☐	For hierarchical and complex designs, identification of the appropriate level for tests and full reporting of outcomes
☒	☐	Estimates of effect sizes (e.g. Cohen's *d*, Pearson's *r*), indicating how they were calculated

Our web collection on statistics for biologists contains articles on many of the points above.

Software and code

Policy information about availability of computer code

Data collection	Cryo-EM micrographs were collected on a Titan Krios G3 microscope (Thermo Fisher Scientific) operated at 300 kV with a K3 direct electron detector (Gatan) in counting mode and BioQuantum Imaging Filter. Additional datasets were acquired on a Glacios microscope (Thermo Fisher Scientific) at 200 kV with a Falcon 4 detector. Movie stacks of 40 frames were recorded with defocus values ranging from 0.9-2.1 µm. Mass photometry measurements were performed on a Refeyn OneMP mass photometer with movies recorded at 100 frames/s.
Data analysis	Cryo-EM data were processed using cryoSPARC v4.5.3. Structural modeling used ChimeraX v1.10.1, WinCoot v0.9.8.96, ISOLDE v1.9, and Phenix v1.20.1-4487. AlphaFold 3 predictions were performed via alphafoldserver.com. Computational analyses used Rosetta v2025.33 and AMBER22. Phylogenetic analyses used MEGA v12 and FigTree v1.4.4. Mass photometry data were analyzed with DiscoverMP v2.0.3. Gel quantification used ImageJ. Statistical analyses used Microsoft Excel and GraphPad Prism.

For manuscripts utilizing custom algorithms or software that are central to the research but not yet described in published literature, software must be made available to editors and reviewers. We strongly encourage code deposition in a community repository (e.g. GitHub). See the Nature Portfolio guidelines for submitting code & software for further information.

Data

Policy information about availability of data

All manuscripts must include a data availability statement. This statement should provide the following information, where applicable:

- Accession codes, unique identifiers, or web links for publicly available datasets
- A description of any restrictions on data availability
- For clinical datasets or third party data, please ensure that the statement adheres to our policy

Cryo-EM micrographs, reconstructions, and atomic models have been deposited in the Electron Microscopy Public Image Archive, the Electron Microscopy Data Bank, and the Protein Data Bank under the following accession codes: WT AaLS/AaRS assemblies: EMPIAR-13016 (https://empiar.org/13016); 12-pentamer RS-bound cage, EMD-54381 (https://www.ebi.ac.uk/emdb/EMD-54381) and PDB 9RYI (https://www.rcsb.org/structure/9RYI); 11-pentamer RS C-termini (C5) cage, EMD-54382 (https://www.ebi.ac.uk/emdb/EMD-54382) and PDB 9RYJ (https://www.rcsb.org/structure/9RYJ); 11-pentamer RS-bound cage, EMD-54383 (https://www.ebi.ac.uk/emdb/EMD-54383) and PDB 9RYK (https://www.rcsb.org/structure/9RYK); 10-pentamer RS-trimer–bound cage, EMD-54385 (https://www.ebi.ac.uk/emdb/EMD-54385) and PDB 9RYM (https://www.rcsb.org/structure/9RYM). WT AaLS: EMPIAR-13017 (https://empiar.org/13017); 12-pentamer cage, EMD-54386 (https://www.ebi.ac.uk/emdb/EMD-54386) and PDB 9RYN (https://www.rcsb.org/structure/9RYN); 11-pentamer cage, EMD-54387 (https://www.ebi.ac.uk/emdb/EMD-54387) and PDB 9RYO (https://www.rcsb.org/structure/9RYO). R29A AaLS: EMPIAR-13018 (https://empiar.org/13018); 12-pentamer cage, EMD-54388 (https://www.ebi.ac.uk/emdb/EMD-54388) and PDB 9RYP (https://www.rcsb.org/structure/9RYP); 11-pentamer cage, EMD-54389 (https://www.ebi.ac.uk/emdb/EMD-54389) and PDB 9RYQ (https://www.rcsb.org/structure/9RYQ). L121A AaLS: EMPIAR-13019 (https://empiar.org/13019); 12-pentamer cage, EMD-54392 (https://www.ebi.ac.uk/emdb/EMD-54392) and PDB 9RYU (https://www.rcsb.org/structure/9RYU); 11-pentamer cage, EMD-54393 (https://www.ebi.ac.uk/emdb/EMD-54393) and PDB 9RYV (https://www.rcsb.org/structure/9RYV). I125A AaLS: EMPIAR-13020 (https://empiar.org/13020); 12-pentamer cage, EMD-54394 (https://www.ebi.ac.uk/emdb/EMD-54394) and PDB 9RYW (https://www.rcsb.org/structure/9RYW); 11-pentamer cage, EMD-54395 (https://www.ebi.ac.uk/emdb/EMD-54395) and PDB 9RYX (https://www.rcsb.org/structure/9RYX).
AlphaFold3 RS/LS complex formation prediction and MD simulation data are available from Rodbuk (DOI: 10.57903/UJ/CVWGR3 (AlphaFold); 10.57903/UJ/TUNWSU (MD)).

Research involving human participants, their data, or biological material

Policy information about studies with human participants or human data. See also policy information about sex, gender (identity/presentation), and sexual orientation and race, ethnicity and racism.

Reporting on sex and gender	n/a
Reporting on race, ethnicity, or other socially relevant groupings	n/a
Population characteristics	n/a
Recruitment	n/a
Ethics oversight	n/a

Note that full information on the approval of the study protocol must also be provided in the manuscript.

Field-specific reporting

Please select the one below that is the best fit for your research. If you are not sure, read the appropriate sections before making your selection.

[x] Life sciences [ ] Behavioural & social sciences [ ] Ecological, evolutionary & environmental sciences

For a reference copy of the document with all sections, see nature.com/documents/nr-reporting-summary-flat.pdf

Life sciences study design

All studies must disclose on these points even when the disclosure is negative.

Sample size	Cryo-EM: Sample sizes were determined by standard practices in single-particle cryo-EM. Datasets contained 5,000-10,000 micrographs per sample, yielding sufficient particles (>100,000) for high-resolution reconstruction. Biochemical assays were performed with n=3-5 independent biological replicates, consistent with standard practice for demonstrating reproducibility. Bioinformatics analysis included >100 bacterial organisms selected to ensure taxonomic diversity and representation across different riboflavin metabolic contexts. No statistical methods were used to predetermine sample sizes.
Data exclusions	Cryo-EM: Particles were excluded based on standard quality criteria during 2D and 3D classification in cryoSPARC. Particles showing poor alignment, heterogeneity, or low resolution features were excluded. These exclusion criteria were pre-established and applied systematically across all datasets. For the AaRS/AaLS complex dataset, 42% of particles corresponded to 12-pentamer cages, 16% to 11-pentamer cages, and 8% to 10-pentamer cages; remaining particles representing unresolved or heterogeneous states were excluded. All exclusion criteria are described in Methods.
Replication	All biochemical experiments (pulldown assays, SDS-PAGE) were performed with n=3-5 independent biological replicates, and all results were successfully reproduced. Cryo-EM datasets represent single preparations; structural reproducibility was confirmed across multiple mutants

	and conditions showing consistent cage architectures. AlphaFold3 predictions were performed in triplicate (three independent runs with different random seeds, yielding 15 models per variant) to assess prediction consistency.
Randomization	n/a
Blinding	n/a

Reporting for specific materials, systems and methods

We require information from authors about some types of materials, experimental systems and methods used in many studies. Here, indicate whether each material, system or method listed is relevant to your study. If you are not sure if a list item applies to your research, read the appropriate section before selecting a response.

Materials & experimental systems

n/a	Involved in the study	
☒	☐	Antibodies
☒	☐	Eukaryotic cell lines
☒	☐	Palaeontology and archaeology
☒	☐	Animals and other organisms
☒	☐	Clinical data
☒	☐	Dual use research of concern
☒	☐	Plants

Methods

n/a	Involved in the study	
☒	☐	ChIP-seq
☒	☐	Flow cytometry
☒	☐	MRI-based neuroimaging

Plants

Seed stocks	n/a
Novel plant genotypes	n/a
Authentication	n/a
