# Supplementary material for: A molecular basis for stoichiometric enzyme encapsulation in the vitamin B2 biosynthesis compartment
Source: Nat Commun. 2026 May 16;17:6498. doi: 10.1038/s41467-026-73260-4 (PMC13376624; doi:10.1038/s41467-026-73260-4)
Supplement: Supplementary file 6 — Source Data [file 41467_2026_73260_MOESM6_ESM.zip › Koziej_etal_2026_Uncropped-gels_R3.docx]

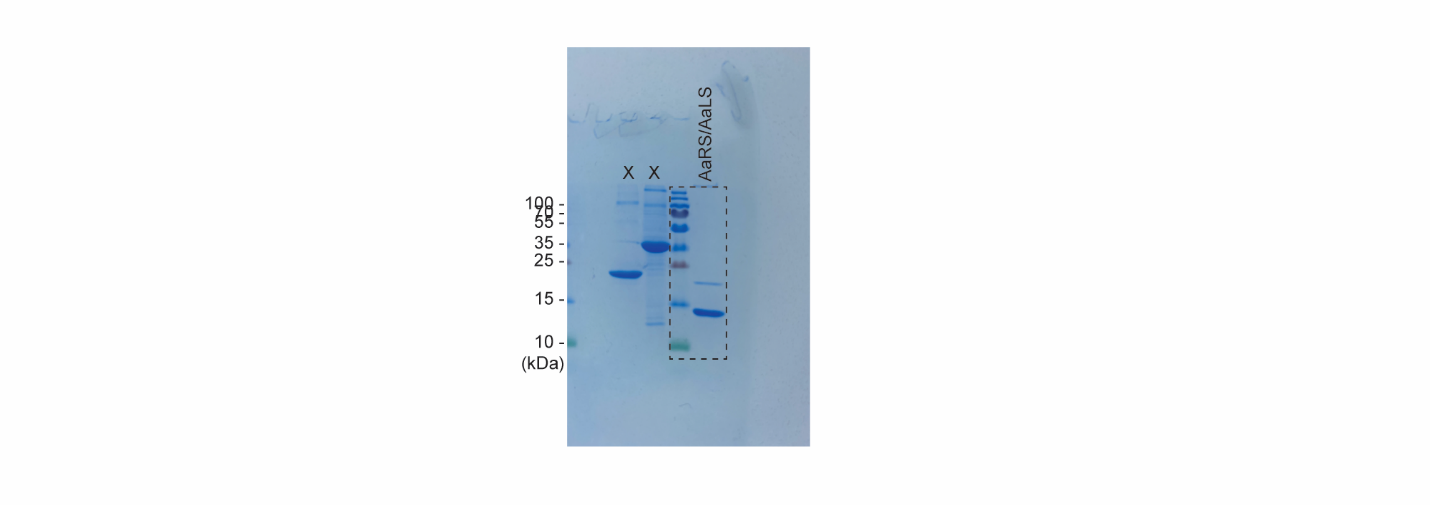


**Uncropped gel 1. Production and purification of AaRS/AaLS complex.** The region surrounded by the dashed line is cropped and shown in Fig. 1e. X indicates samples unrelated to this study.


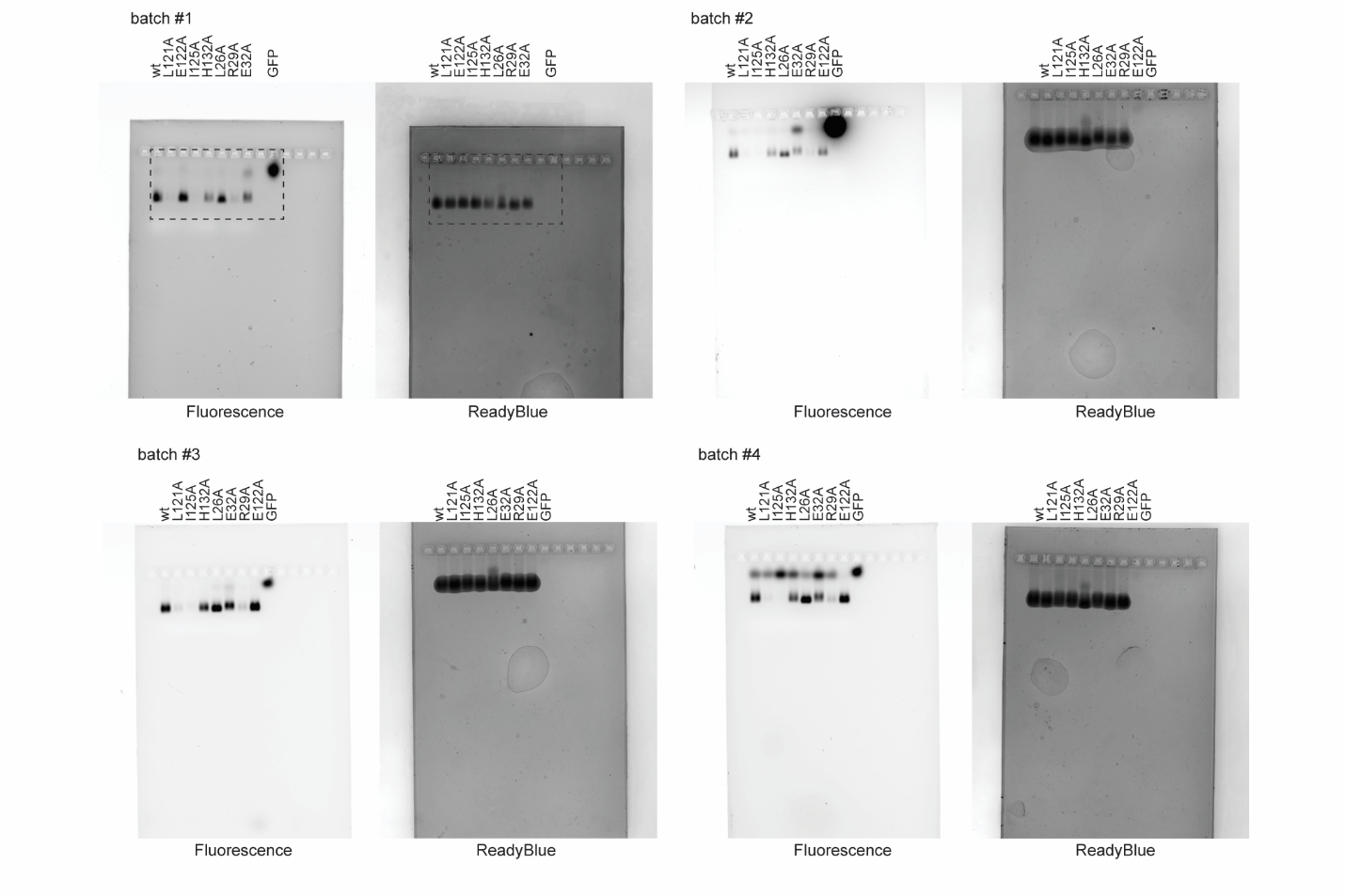


**Uncropped gel 2. Pulldown assay for GFP-CLS and AaLS mutants.** The regions surrounded by the dashed lines in batch #1 gel images are cropped and shown in Fig. 2e. Sample loading orders are different between batches. In batch #4, the upper fluorescent bands correspond to free GFP-CLS, which was likely not completely removed during affinity purification.


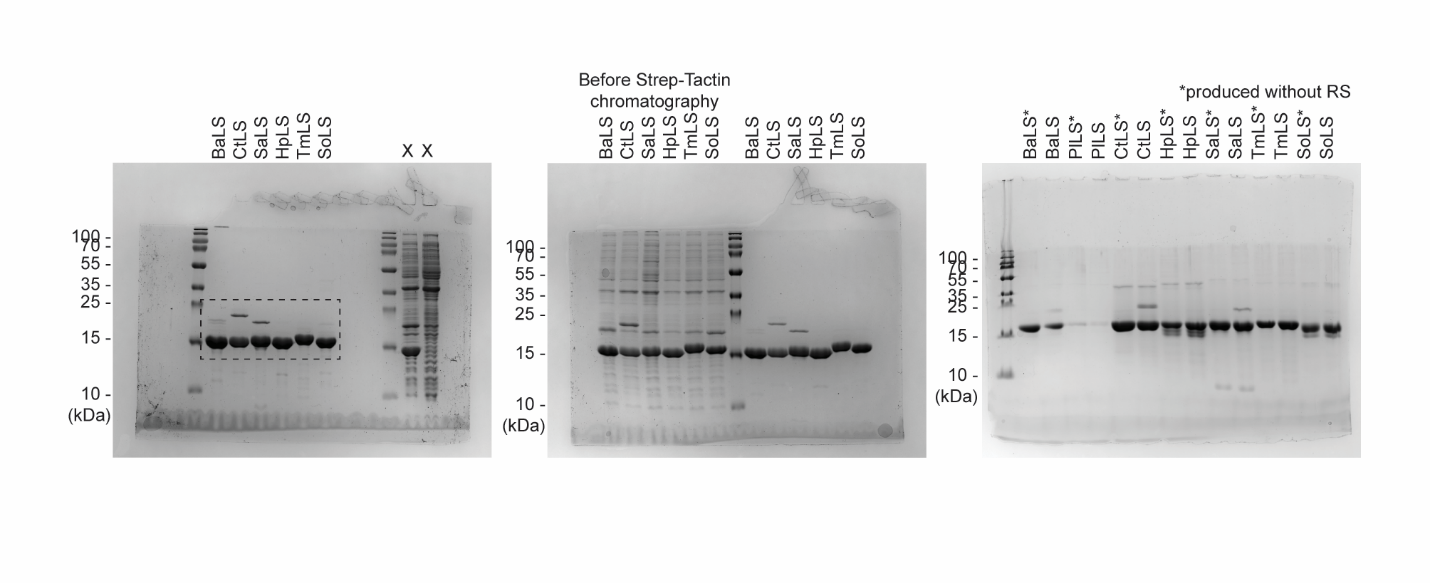


**Uncropped gel 3. Pulldown assay for RS/LS from different organisms.** The region surrounded by the dashed line in the left gel image is cropped and shown in Fig. 4e. X indicates samples unrelated to this study. PlLS corresponds to lumazine synthase from *Photobacterium leiognathi*, which was excluded from the later repetitions due to the poor production level in *E. coli*. Asterisks (*) indicate LSs produced alone (without coproduction with their cognate RSs).
