# Supplementary material for: A molecular basis for stoichiometric enzyme encapsulation in the vitamin B2 biosynthesis compartment
Source: Nat Commun. 2026 May 16;17:6498. doi: 10.1038/s41467-026-73260-4 (PMC13376624; doi:10.1038/s41467-026-73260-4)
Supplement: Supplementary file 5 — Transparent Peer Review file [file 41467_2026_73260_MOESM5_ESM.pdf]

nature portfolio

Peer Review File

A molecular basis for stoichiometric enzyme encapsulation in the vitamin B2 biosynthesis compartment

Corresponding Author: Dr Yusuke Azuma

This file contains all reviewer reports in order by version, followed by all author rebuttals in order by version.

Version 0:

Reviewer comments:

Reviewer #1

(Remarks to the Author)
Koziej and coworkers present an elegant study on a proposed hierarchical assembly of the vitamin B2 biosynthesis compartment that leads to stoichiometric encapsulation of the enzyme. The two main proteins of interest are the pentamer-forming lumazine synthase (LS) protein and the homotrimeric riboflavin synthase (RS) enzyme. Through a combination of cryo-EM, Rosetta modeling, and MD simulations, the authors provide compelling evidence that the recruitment of the RS to the interior of the LS shell is a necessary step of the process to prevent additional enzyme recruitment (beyond the trimer) and to prevent premature closure before the RS trimer can form. That being said, there are a few details in this mechanism that are (in my opinion) speculative, which could potentially be addressed. Nonetheless, this manuscript is certainly of interest to the broad question of how compartmentalization can be regulated. Please see my specific comments below.

1. The authors propose that the binding between the CLS peptide and the pre-C3 pentamers leads to a “gate and lock” mechanism mediated by the “up” state of loop 129-133. So, if I understand the gating mechanism correctly, the CLS/pre-C3 complex occludes the final pentamer from binding until the final step, after which CLS presumably dissociates in favor of the final pentamer competing the protein cage. If that’s the case, is there an energetic driving force for this series of steps? Is the idea that one corner of the pentamer is insufficient to displace one CLS peptide but the final step (which involves 5 corners) has enough of a thermodynamic driving force to move up to 3 CLS peptides? Can the authors assess free energies via Rosetta or related techniques to identify if this final step is thermodynamically favored?

2. The mechanism shown in Fig. 5 seems to be contingent on pre-assembly of the RS homotrimer. If so, I can see how binding of the RS trimer to the nucleated LS cage would eventually lead to complete RS/LS complexes where (1) overloading of enzyme is prevented and (2) the final pentamer completes the complex. But I don’t see anything in the mechanism that would prevent empty LS cages and LS cages with insufficient loading (i.e., if only a RS monomer binds to the nucleated LS cage before additional LS closes the cage). It seems more likely that there is a distribution of four end states: empty cages, insufficiently loaded cages, complete complexes, and incomplete complexes (such as the 11-pentamer and 10-pentamer particles that the authors see). Can the authors quantify or comment on the distribution of these states? Is the complete RS/LS complex favored and if so, why? Again, I can see an argument for the prevalence of the complete and incomplete RS/LS complexes if the RS equilibrium is greatly in favor of homotrimers compared to monomers.

3. Related to my comment above, if the RS-RS binding free energy is much stronger than the RS(CLP)-pentamer interaction or LS-LS interaction required to form pentamers, then the proposed mechanism is also much more likely. The latter is under the assumption that CLP only interacts with the pre-C3 interface (requiring two pentamers to form then associate first). Can the authors perform those calculations either using MD or Rosetta?

Reviewer #2

(Remarks to the Author)
Article Overview: In the manuscript "A molecular basis for stoichiometric enzyme encapsulation in the vitamin B2 biosynthesis compartment," Koziej et al. utilize cryo-EM to characterize the assembly mechanism of the Aquifex aeolicus riboflavin synthase (AaRS) and lumazine synthase (AaLS) complex. The authors identify an anchoring mechanism where the AaRS cage-localization signal (CLS) targets the pentamer interfaces of incomplete AaLS cages, suggesting a

conformational gate that extends the loading window. They further describe a "molecular lock" triggered by final cage closure that stabilizes the complex and prevents disassembly. This mechanism is then used to predict the prevalence of such compartments across diverse bacterial taxa, suggesting a modular and evolutionarily labile encapsulation strategy.

Overall Evaluation: This work is high-quality and technically impressive, particularly in its successful trapping and reconstruction of rare 10- and 11-pentamer assembly intermediates. The "molecular lock" provides a notable and compelling structural explanation for stoichiometric control that likely has broad interest in synthetic biology. However, the manuscript currently suffers from significant logical inconsistencies regarding the native state of the cargo (trimer vs. monomer) and overstates the novelty of interface-targeting compared to established bacterial microcompartment (BMC) literature. Given this framing, I believe this article is largely appropriate for Nature Communications but needs major revisions to address the inconsistencies at the heart of their mechanistic model. If the authors can meet these critiques, I would be happy to recommend their article for eventual publication in Nature Communications. Below I detail my concerns which I hope the authors find useful in improving the quality of this otherwise technically impressive manuscript.

Major Concerns:
• The current assembly model lacks internal consistency and leaves several significant logical gaps:
o Structural Inconsistencies: While the manuscript characterizes AaRS as a stable homotrimer in Figure 1d, the proposed mechanism in Figure 5 explicitly invokes "RS monomers" and a step-wise loading pathway.
o Logical Redundancy of Anti-Cooperativity: If the cage only accommodates a single trimer and its occupancy physically blocks further loading, the necessity of an "anti-cooperative" delay is unclear. Once the trimer is anchored, a mechanism favoring immediate closure would be more evolutionarily intuitive than one that maintains a "transiently open state".
o Functional Risks of Delayed Closure: The finding that RS presence actively hinders LS cage completion suggests that RS expression spikes could paradoxically inhibit the formation of functional, closed compartments altogether.
o Recommendation: The molecular logic of an extended loading window for a single-step incorporation event appears unnecessary in this framing. I would appreciate greater clarity on why the system would evolve to delay closure once the stoichiometric limit has already been met.
• The transition from the “up” to the “down” loop configuration lacks a rigorous mechanistic explanation:
o Stability Paradox: If CLS occupancy actively stabilizes the "up" conformation to maintain an open assembly, what specifically triggers the transition to the locked "down" state?
o Insufficient Justification: The reliance on "spontaneous fluctuations" and kinetic "patience" to explain final closure feels speculative rather than mechanistic.
o Recommendation: The authors should provide a more robust justification, potentially supported by energy landscape analysis, for how the system overcomes the loop-mediated barrier to engage the final R29-E32 molecular lock.
• The experimental results for the L26A mutation appear inconsistent with the structural model provided:
o Structural Contradiction: Figure 2c identifies L26 as a key residue supporting the hydrophobic packing of the CLS against both LS subunits. However, the pull-down assay in Figure 2e shows that the L26A mutation results in negligible loss of interaction efficiency (<30% change) compared to the 85–96% drops seen in other interface residues like L121A or I125A.
o Functional Conservation: The authors should clarify if an alanine substitution at this specific position is functionally conserved or if the local hydrophobic environment is robust enough to tolerate the smaller side chain without destabilizing the CLS anchor.
o Validity Check: To ensure the validity of the proposed binding mode, the authors should address this outlier and discuss why this specific hydrophobic contact is dispensable while neighboring ones are essential.
• Page 7, line 136: The way this assertion currently reads is that no other protein compartment uses the shell-shell assembly interface as a cargo target. This is not true, as this has been observed in carboxysomes (PMID: 37679318) and metabolosomes (PMID: 38735476). Cargo anchoring on pre-tessellated shell units, and their assembly interfaces, are more commonly the rule as opposed to an exception. However, I believe the authors can still get a “win” here by reframing their findings as targeting the “actively assembling interface” where a structural gap from the missing pentamer still exists. This is more likely to be new and is highly relevant for the molecular lock argument later in the manuscript. Apologies if this was the original intent, but the text as-is can give an improper impression.
• Page 20, lines 399-405: Bacterial microcompartment shells can autonomously assemble (PMID: 28642439 is the first of many polyhedral examples, “other” morphologies also exist PMID: 36932732, PMID: 36786617, PMID: 41268919). The role of condensation mechanisms in facilitating carboxysome and metabolosome formation (PMID: 32123388, PMID: 40790117, PMID: 35614058, plus the current citation #13) also inherently implies cooperative assembly with guest enzymes. Further, shell components can help to template bacterial microcompartment formation by providing seed sites (so called “shell-first”) for nucleation cascades (PMID: 35614058, PMID: 38425792 reviews both shell-first and other pathways). Please update this passage with these prior observations in mind.

Smaller Concerns:
• Page 20, lines 406-408: It may be worth comparing the stability of LS cages to findings from encapsulins and bacterial microcompartments, if available. It would help to drive this point home.
• Double check that “lumenal” is intended as opposed to “luminal”. The latter is more common, but I believe both are acceptable.
• Page 7, line 132: I believe the Figure reference should be for Fig. 1h, not 2h.
• Page 11, line 216: I believe “geometry” should be “geometries”.
• Out of my own curiosity, when the conformational lock occurs upon final pentamer insertion, are the AaRS proteins “dislodged” from their anchor site? What is their fate within the LS cage?

Reviewer #3

(Remarks to the Author)
Koziej et al. have presented a molecular basis for encapsulation of homotrimeric riboflavin synthase (RS) by the dodecahedron lumazine synthase (LS) protein cage in Aquifex aeolicus: i.e., the AaRS/AaLS complex. They disclose several high-quality cryo-EM maps and models of the empty AaLS and AaRS/AaLS complex to support their model for how the cage-localization signal (CLS) peptide of AaRS docks to the lumenal surface at pentamer-pentamer interfaces of the AaLS cage during assembly. They used a CLS-GFP mimic of the natural AaRS cargo to measure cage assembly through native-gel fluorescence and leveraged these data to explain how the fully mature AaRS/AaLS complex prevents self-disassembly through a stabilizing electrostatic interaction. They further co-opted MD and Rosetta calculations to interrogate the importance of residue interactions between CLS and AaLS protomers of the AaRS/AaLS complex. Finally, they employed phylogenetic analysis and sequence alignments to understand how prevalent these mechanisms for RS/LS cage formation are across different species and kingdoms of life. While the authors present high-quality data and associated analyses, they should try to improve the data presentation so it (1) is more intuitive to non-experts and (2) completely satisfies their claims (see specific comments below). In addition, the reviewer has identified some concerns (detailed below) that should be addressed before this manuscript is published. Most importantly, the proposed “gate and lock” mechanism, although interesting, appears to be not fully substantiated by the data. As suggested below, the authors may consider alternative approaches to testing this idea (e.g., cargo exchange experiments or more convincing electrophoretic analysis). The extrapolations made to other RS/LS systems in nature was also not fully supported by the data and should be addressed (see below).
1. Pg. 3, line 50: Can the authors be more specific about where the RS/LS complex occurs in nature, at least at the kingdom level?
2. Pg. 4, line 71: Please include RS:LS in reference to 3:60 stoichiometry for readers.
3. Fig. 1: Please draw the phosphoryl groups in their appropriate charged states at near-neutral pH.
4. Extended Data Fig. 1: It is difficult to understand how the text and images match in this figure. Can the authors revise this figure, perhaps with addition of arrows and numerical steps, to make it easier to interpret?
5. Pg. 7, line 132: There appears to be an incorrect reference to Fig. 2h.
6. Pg. 7, lines 134-135: It would be useful to include a close-up model of the red helix interacting with the clearly demarcated “pre-C3” area. Otherwise, it is hard to gather what it meant by this with the very small panels in Fig. 1h-j.
7. Pg. 7, lines 138-139: Why is asymmetry anticipated when there are many C3 axes of symmetry in the dodecahedron? Could one not also imagine three, symmetry-related binding sites for the CLS peptides?
8. Pg. 7, lines 138-142: Again, it is quite difficult to make sense of this from the very small figure panels. Can the authors consider breaking Fig. 1 into two separate main text figures or making surrounding panels (e.g, panel k) smaller to increase the size of the cryo-EM maps? Currently, Fig. 1k is larger than necessary, and it is also lacking labels. Ideally, the authors would show models of RS/LS fit inside the maps to support these statements, at least as an SI figure.
9. Pg. 8, lines 143-146: Fundamentally, the RS heterotrimer must be encapsulated by the pre-formed cage before the pore size becomes too small for entry. Can the authors comment on the oligomeric states of LS that would be compatible with RS entry based on these simple geometric criteria? It would be helpful to delineate what can be logically deduced and what the authors present as new knowledge regarding the timing of events during cage assembly.
10. Pg. 8, lines 150-154: Can reference to a model be made for this structural description? It is difficult to understand what is meant from these words alone.
11. Pg. 8, lines 161-163: A reference to the relevant model and map should be made here. Ideally, the model fit inside the map should be shown.
12. Fig. 2: Can the authors please label all the residues shown in this figure (e.g., panel b)?
13. Fig. 2: Why is GFP not stained by readyblue?
14. Pg. 11, line 215: Can the authors express “<±30%” in a more sensible way?
15. Pg. 12, line 247: Can the authors explain what is meant by “the incoming partner” and “units” in these two sentences?
16. Pgs. 12-13, lines 250-255: Is there any evidence for anti-cooperative cage assembly, and if so, can the authors please cite it?
17. Pg. 14, lines 288-291 (part 1): The authors should explain why GFP does not stain with readyblue to support the idea that “unbound GFP-CLS” is truly present. In other words, shouldn’t there be a loading control with only GFP-CLS to know where it migrates? Presumably this is why GFP was loaded, but why then is the protein not visible with Coomassie blue (readyblue) stain?
18. Pg. 14, lines 288-291 (part 2): Have the authors considered analyzing the relative rates of cargo exchange by adding purified RS to LS-encapsulated GFP-CLS (or conversely, by adding purified GFP-CLS to LS-encapsulated RS)? If the proposed gate and lock mechanism is truly mediated by E32, then the authors might expect faster (or higher yield) exchange for the E32A mutant relative to WT.
19. Fig. 4, panel b: Can the details of BsRS/BsLS be more closely examined (at least in the SI) to show how the CLS peptide engages with the LS surface at a pre-C3 (or related) site? This would help to understand how general the binding mode of AaRS/AaLS is.
20. Fig. 4, line 321: What are the criteria for organisms to be predicted to form RS/LS complexes?
21. Fig. 4, line 326: Can the authors explain why SoLS is not higher in RS/LS band intensity ratio? There is a visible band above SoLS that runs consistent with RS in the neighboring wells. By eye, it does appear to be higher than plotted below by densitometry, but this may not be so.
22. Pg. 16, line 336: Like the above comment, it is not clear how these predictions are made. Can the authors comment?
23. Pg. 17, lines 340: “Complete concordance” does not sit well with the reader when the AF models (Ext. Data Fig. 7) only show two out of the six RS/LS pairs tested in Fig. 4e. Furthermore, the graphics in Ext. Data Fig. 7 could be better labeled, and close-up views of the modeled CLS-LS interactions as in Fig. 2a-c would be helpful to support the authors’ conclusions.

Version 1:

Reviewer comments:

Reviewer #1

(Remarks to the Author)
Koziej and coworkers have performed additional analysis via Rosetta InterfaceAnalyzer to try to address the question I posed on thermodynamic driving forces for when the pentamer will preferentially associate. I appreciate this additional analysis, and I think the picture of the mechanism is much clearer. Of course, some elements of the proposed mechanism are still speculative, but at the same time, I recognize that the current model and interpretation is perhaps the best that can be done at this point. Given the broad interest of the proposed “gate and lock” mechanism and the integrated biochemical, structural, and computational analysis, I recommend publication.

Reviewer #2

(Remarks to the Author)
I would like to thank the authors for thoroughly, patiently and rigorously addressing each of my original comments. I have no further comments to add. As such, I believe this article is in excellent standing and strongly endorse its publication in Nature Communications.

Reviewer #3

(Remarks to the Author)
Thank you for adequately addressing all comments. The revised manuscript is substantially improved and (in my opinion) worthy of acceptance in this journal.

Open Access This Peer Review File is licensed under a Creative Commons Attribution 4.0 International License, which permits use, sharing, adaptation, distribution and reproduction in any medium or format, as long as you give appropriate credit to the original author(s) and the source, provide a link to the Creative Commons license, and indicate if changes were made.
In cases where reviewers are anonymous, credit should be given to 'Anonymous Referee' and the source.
The images or other third party material in this Peer Review File are included in the article’s Creative Commons license, unless indicated otherwise in a credit line to the material. If material is not included in the article’s Creative Commons license and your intended use is not permitted by statutory regulation or exceeds the permitted use, you will need to obtain permission directly from the copyright holder.
To view a copy of this license, visit https://creativecommons.org/licenses/by/4.0/

Reviewer #1 (Remarks to the Author):

Koziej and coworkers present an elegant study on a proposed hierarchical assembly of the vitamin B2 biosynthesis compartment that leads to stoichiometric encapsulation of the enzyme. The two main proteins of interest are the pentamer-forming lumazine synthase (LS) protein and the homotrimeric riboflavin synthase (RS) enzyme. Through a combination of cryo-EM, Rosetta modeling, and MD simulations, the authors provide compelling evidence that the recruitment of the RS to the interior of the LS shell is a necessary step of the process to prevent additional enzyme recruitment (beyond the trimer) and to prevent premature closure before the RS trimer can form. That being said, there are a few details in this mechanism that are (in my opinion) speculative, which could potentially be addressed. Nonetheless, this manuscript is certainly of interest to the broad question of how compartmentalization can be regulated. Please see my specific comments below.

> We appreciate reviewer 1 for being interested in this study. We agree that the proposed mechanism contains our speculations. Meanwhile, they are our best speculation that plausibly explains the observed results and their natural function. The details are described below.

1. The authors propose that the binding between the CLS peptide and the pre-C3 pentamers leads to a "gate and lock" mechanism mediated by the "up" state of loop 129-133. So, if I understand the gating mechanism correctly, the CLS/pre-C3 complex occludes the final pentamer from binding until the final step, after which CLS presumably dissociates in favor of the final pentamer competing the protein cage. If that's the case, is there an energetic driving force for this series of steps? Is the idea that one corner of the pentamer is insufficient to displace one CLS peptide but the final step (which involves 5 corners) has enough of a thermodynamic driving force to move up to 3 CLS peptides? Can the authors assess free energies via Rosetta or related techniques to identify if this final step is thermodynamically favored?

> We thank the reviewer for this important question about the energetic driving force for CLS displacement and final pentamer docking. We performed Rosetta InterfaceAnalyzer calculations to assess the thermodynamic favorability of the final assembly step. The simulations were carried out for two neighboring AaLS pentamers binding to either CLS or another AaLS protomer. The results suggest that pentamer-pentamer binding is thermodynamically much favored over CLS binding, with approximately 1.6-fold difference in ΔΔG.

The reviewer's interpretation regarding stoichiometry is insightful but appears unnecessary. The thermodynamic advantage of pentamer binding is observed even in single binding site comparisons (1 vs 1). This raises an important question the reviewer implies, how CLS remain bound at pre-C3 sites during assembly. We propose this reflects binding site selectivity. During early and intermediate assembly stages of AaLS, multiple open pre-C3 sites are available and upcoming pentamers likely bind to CLS-free sites rather than displacing CLS from occupied sites. This physical block delays pentamer docking specifically at CLS-occupied sites until the completion becomes necessary. At this final assembly step, the final pentamer must overcome CLS blocking to dock where the precise mechanism driving this displacement remains unknown. However, once displacement occurs and the final pentamer docks, this state should be thermodynamically favored. We have added the new energy calculation data in **Fig. 3e,f** and discuss the role of thermodynamic preference in the final pentamer docking in the corresponding results section (**page 16, line 321-331**).

2. The mechanism shown in Fig. 5 seems to be contingent on pre-assembly of the RS homotrimer. If so, I can see how binding of the RS trimer to the nucleated LS cage would eventually lead to complete RS/LS complexes where (1) overloading of enzyme is prevented and (2) the final pentamer completes the complex. But I don't see anything in the mechanism that would prevent empty LS cages and LS cages with insufficient loading (i.e., if only a RS monomer binds to the nucleated LS cage before additional LS closes the cage). It seems more likely that there is a distribution of four end states: empty cages, insufficiently loaded cages, complete complexes, and incomplete complexes (such as the 11-pentamer and 10-pentamer particles that the authors see). Can the authors quantify or comment on the distribution of these states? Is the complete RS/LS complex favored and if so, why? Again, I can see an argument for the prevalence of the complete and incomplete RS/LS complexes if the RS equilibrium is greatly in favor of homotrimers compared to monomers.

> We thank the reviewer for this important question about the distribution of assembly states. As described in the discussion section, we propose three mechanisms that prevent empty and insufficiently loaded cages, where two of them, anti-cooperative LS assembly and physical block by CLS binding, extend the time window for proper RS trimer recruitment, addressing the reviewer's concern about premature cage closure with only RS monomers bound. Additionally, cooperative assembly between RS and LS, whereby multiple pre-C3 sites recruit RS and multiple CLS peptides recruit LS pentamers, kinetically promoting heteroassembly formation. These mechanisms represent our interpretation based on the observed binding mode and multivalent interaction architecture.

The reviewer is correct that RS homotrimer formation is favored. Isolation of recombinantly expressed RS alone shows trimeric species (Fig. 1d), confirming this as the inherent equilibrium state of the enzyme. Fig. 5 depicts potential mechanisms that may occur during cellular co-production of RS and LS, recognizing that many of the proposed intermediates are experimentally undetectable (we have added this status in **Fig. 5 legend**). Whether LS cages containing only RS monomers exist remains unclear, as such species are undetectable by cryo-EM. We believe these immature species may exist transiently but likely represent a minimal population, as the described regulatory mechanisms are designed to prevent formation of such unwanted cage species. Quantifying the distribution of end states is challenging given that our structural methods capture predominantly stable, well-formed complexes and late-stage intermediates, with transient or unstable species remaining below the detection threshold.

3. Related to my comment above, if the RS-RS binding free energy is much stronger than the RS(CLP)-pentamer interaction or LS-LS interaction required to form pentamers, then the proposed mechanism is also much more likely. The latter is under the assumption that CLP only interacts with the pre-C3 interface (requiring two pentamers to form then associate first). Can the authors perform those calculations either using MD or Rosetta?

> We appreciate the reviewer's suggestion regarding energy calculations. However, we believe that the proposed cooperative assembly mechanism operates independently of the relative binding energies. Fig. 5 depicts both potential pathways (middle pathway showing monomer binding followed by trimerization, and left pathway showing pre-formed trimer binding) because the order of RS trimerization relative to LS binding remains unclear and may occur through either route. The multivalent interaction architecture provides kinetic advantage for heterocomplex formation regardless of which pathway predominates. We

apologize that these points were not sufficiently clear in the original figure. We have revised the **Fig. 5 legend** to clarify that the pathways illustrate representative examples of potential assembly routes, and that cooperative binding can occur through alternative routes beyond those depicted, such as two pentamers binding to an RS trimer followed by recruitment of additional pentamers to form further pre-C3 sites.

Reviewer #2 (Remarks to the Author):

Article Overview: In the manuscript "A molecular basis for stoichiometric enzyme encapsulation in the vitamin B2 biosynthesis compartment," Koziej et al. utilize cryo-EM to characterize the assembly mechanism of the Aquifex aeolicus riboflavin synthase (AaRS) and lumazine synthase (AaLS) complex. The authors identify an anchoring mechanism where the AaRS cage-localization signal (CLS) targets the pentamer interfaces of incomplete AaLS cages, suggesting a conformational gate that extends the loading window. They further describe a "molecular lock" triggered by final cage closure that stabilizes the complex and prevents disassembly. This mechanism is then used to predict the prevalence of such compartments across diverse bacterial taxa, suggesting a modular and evolutionarily labile encapsulation strategy.

Overall Evaluation: This work is high-quality and technically impressive, particularly in its successful trapping and reconstruction of rare 10- and 11-pentamer assembly intermediates. The "molecular lock" provides a notable and compelling structural explanation for stoichiometric control that likely has broad interest in synthetic biology. However, the manuscript currently suffers from significant logical inconsistencies regarding the native state of the cargo (trimer vs. monomer) and overstates the novelty of interface-targeting compared to established bacterial microcompartment (BMC) literature. Given this framing, I believe this article is largely appropriate for Nature Communications but needs major revisions to address the inconsistencies at the heart of their mechanistic model. If the authors can meet these critiques, I would be happy to recommend their article for eventual publication in Nature Communications. Below I detail my concerns which I hope the authors find useful in improving the quality of this otherwise technically impressive manuscript.

> We appreciate reviewers 2 for evaluating this study would be largely appreciated in the field of synthetic biology. We believe that there is no logical inconsistency in this manuscript, but our description might not sufficiently explain the proposed mechanism. We have reflected the reviewer’s comments to remove potential confusion from the readers and corrected misleading statements.

Major Concerns:

• The current assembly model lacks internal consistency and leaves several significant logical gaps:

o Structural Inconsistencies: While the manuscript characterizes AaRS as a stable homotrimer in Figure 1d, the proposed mechanism in Figure 5 explicitly invokes "RS monomers" and a step-wise loading pathway.

> We thank the reviewer for this comment. We clarify that there is no inconsistency between these descriptions. Fig. 1d characterizes the final isolated product where AaRS exists as homotrimers, the kinetically favored quaternary state of the enzyme. In contrast, Fig. 5 depicts the assembly process itself during which all precursor states, including RS monomers and partially assembled AaLS cage intermediates, must transiently exist before reaching the final assembled state.

Fig. 5 represents our inference of the assembly mechanism occurring within cells during co-production. The stepwise monomer pathway is shown to illustrate why multivalent binding by RS trimers is kinetically favored. RS trimers display three CLS peptides that can engage multiple pre-C3 sites on nascent LS cages simultaneously, while individual monomer binding would be kinetically unstable. We

do not experimentally observe these assembly intermediates, as we can only isolate and characterize the final products and the late-stage assembly intermediates we successfully captured in this study.

We have revised **Fig. 5 legend** to clarify that the trimer-bound intermediates are kinetically favored over monomer-bound forms due to multivalent binding, and that these intermediates represent theoretical models inferred from the observed binding site architecture rather than directly observed assembly states.

o Logical Redundancy of Anti-Cooperativity: If the cage only accommodates a single trimer and its occupancy physically blocks further loading, the necessity of an "anti-cooperative" delay is unclear. Once the trimer is anchored, a mechanism favoring immediate closure would be more evolutionarily intuitive than one that maintains a "transiently open state".

> Anti-cooperative assembly and physical blocking address distinct quality control challenges and are not redundant. Anti-cooperative assembly is an inherent property of AaLS cage formation, where the 129-133 loop provides a kinetic barrier that extends the time window for cargo loading, preventing premature formation of empty cages. The physical blocking mechanism provided by AaRS addresses the complementary problem of preventing overloading, as the spatial constraints imposed by the coiled-coil arrangement ensure that only one RS trimer can be accommodated within the lumen.

We highly appreciate the reviewer raising an excellent point about cage closure dynamics. If "its occupancy physically blocks further loading" refers to CLS-mediated blocking of final pentamer docking, then we agree entirely with the reviewer's logic. Once RS trimer binding is complete, immediate cage closure would indeed be intuitive. However, our experimental observations show the opposite. Co-production with AaRS increases the population of 10- and 11-pentamer AaLS intermediates, indicating that CLS-mediated anchoring at pentamer-pentamer interfaces actually blocks cage completion rather than promoting it.

We have been actively exploring whether RS maturation to trimers might signal or accelerate closure, as the reviewer suggests, but we have not yet obtained any evidence for such a mechanism. Our current structural data show that incomplete cages with bound RS maintain the open loop conformation, but the molecular details of how and when closure is triggered remain to be determined.

o Functional Risks of Delayed Closure: The finding that RS presence actively hinders LS cage completion suggests that RS expression spikes could paradoxically inhibit the formation of functional, closed compartments altogether.

> We agree with the reviewer that the proposed mechanisms carry inherent functional risks. The observation that RS anchoring delays cage completion raises important questions about how cells ensure robust compartment formation under varying expression conditions.

Much remains unknown about the regulatory layers that govern this system in native organisms. All experiments described in this manuscript were performed using recombinant expression in *E. coli*, which may not fully recapitulate the native assembly environment. Presumably, additional mechanisms exist to ensure proper complex formation, such as coordinated regulation of RS and LS expression levels.

It is also unclear how much assembly accuracy is actually required for cells to maintain sufficient vitamin B2 biosynthesis, as some degree of empty or incomplete cage formation may be tolerated. We acknowledge that what we describe here is a molecular-level mechanism revealed by capturing complex formation intermediates. Comprehensive understanding of RS/LS complex assembly regulation in native organisms will require investigating these additional layers of cellular control beyond the structural mechanisms we present.

o Recommendation: The molecular logic of an extended loading window for a single-step incorporation event appears unnecessary in this framing. I would appreciate greater clarity on why the system would evolve to delay closure once the stoichiometric limit has already been met.

> We agree with the reviewer that the continued delay after the stoichiometric limit is met appears counterintuitive. The system appears to prioritize preventing empty cage formation, which would be economically costly as a waste of synthesized proteins. This prevention comes at the expense of delayed closure, resulting in accumulation of incomplete but cargo-loaded intermediates (10- and 11-pentamer states). Whether this trade-off represents evolutionary optimization or simply a consequence of the binding geometry remains unclear. Our phylogenetic observation that organisms with low vitamin requirements still maintain RS/LS complex formation capacity suggests that either strict stoichiometric precision is not essential under all conditions or that even incomplete assembly provides sufficient catalytic benefit. We have added discussion text (**page 24, lines 478-490**) acknowledging these uncertainties and the need for quantitative examination in native biological contexts.

• The transition from the “up” to the “down” loop configuration lacks a rigorous mechanistic explanation:
o Stability Paradox: If CLS occupancy actively stabilizes the "up" conformation to maintain an open assembly, what specifically triggers the transition to the locked "down" state?
o Insufficient Justification: The reliance on "spontaneous fluctuations" and kinetic "patience" to explain final closure feels speculative rather than mechanistic.
o Recommendation: The authors should provide a more robust justification, potentially supported by energy landscape analysis, for how the system overcomes the loop-mediated barrier to engage the final R29-E32 molecular lock.

> We thank the reviewer for these important critiques. We first wish to clarify our interpretation of CLS function in this context. The 129-133 loop adopts the "up" conformation in all incomplete assemblies regardless of CLS presence, as observed in both guest-free and guest-loaded structures. Our interpretation is that CLS-mediated blocking functions primarily by occupying the same pentamer-pentamer interface that the final pentamer must engage, physically obstructing pentamer docking.

The reviewer is correct that the driving force for loop transition from "up" to "down" remains unknown. To address the request for energy landscape analysis, we performed Rosetta free energy calculations to characterize the energetic basis of this transition. Calculations on isolated AaLS protomer extracted from the corresponding cryo-EM structures reveal that the "up" state is inherently energetically favored, with W137 stabilized by interactions with the loop region, confirming the existence of an energy barrier for the loop transition. We propose thermal fluctuations and mechanical displacement as plausible mechanisms for overcoming this barrier. Yet, we cannot currently distinguish which mechanism

predominates, or whether both contribute, thus remaining both possibilities in the main text. Importantly, regardless of which mechanism drives the loop transition, the proposed anti-cooperative assembly and molecular lock mechanisms function to extend the guest loading window and ensure irreversible cage completion.

As described in our response to Reviewer 1, Rosetta InterfaceAnalyzer calculations comparing AaLS protomer binding to two adjacent pentamers versus CLS reveal that the completed assembly state is energetically favorable. While the loop conformational change mechanism yet remains unknown, these calculations demonstrate that once the transition occurs, the final state with pentamer docked and R29-E32 lock formed is thermodynamically stable and prevents reversal.

We have modified the manuscript text to include these Rosetta calculations and to clarify that the proposed mechanisms for how the barrier is overcome represent our interpretation of structural observations (**Fig. 3d, page 13 line 271-277**).

• The experimental results for the L26A mutation appear inconsistent with the structural model provided:
o Structural Contradiction: Figure 2c identifies L26 as a key residue supporting the hydrophobic packing of the CLS against both LS subunits. However, the pull-down assay in Figure 2e shows that the L26A mutation results in negligible loss of interaction efficiency (<30% change) compared to the 85–96% drops seen in other interface residues like L121A or I125A.
o Functional Conservation: The authors should clarify if an alanine substitution at this specific position is functionally conserved or if the local hydrophobic environment is robust enough to tolerate the smaller side chain without destabilizing the CLS anchor.
o Validity Check: To ensure the validity of the proposed binding mode, the authors should address this outlier and discuss why this specific hydrophobic contact is dispensable while neighboring ones are essential.

> We thank the reviewer for this insightful observation. The modest effect of the L26A mutation requires clarification regarding its structural role. While L26 participates in the hydrophobic packing shown in Fig. 2c, its primary function is to support the positioning of surrounding residues that form the hydrophobic surface, particularly the neighboring I125, rather than to make extensive direct contacts with the CLS peptide. The CLS-LS interface formed directly by L26 is relatively small compared to those formed by L121 or I125. The L26A mutation likely reduces structural support for I125 positioning, but this perturbation is evidently insufficient to significantly disrupt CLS binding, as I125 appears to maintain its approximate position despite the reduced support. We have added a brief clarification in the main text (**page 12, lines 237-239**) explaining L26's structural role and why L26A retains full CLS binding efficiency despite its position in the hydrophobic packing region.

• Page 7, line 136: The way this assertion currently reads is that no other protein compartment uses the shell-shell assembly interface as a cargo target. This is not true, as this has been observed in carboxysomes (PMID: 37679318) and metabolosomes (PMID: 38735476). Cargo anchoring on pre-tessellated shell units, and their assembly interfaces, are more commonly the rule as opposed to an

exception. However, I believe the authors can still get a “win” here by reframing their findings as targeting the “actively assembling interface” where a structural gap from the missing pentamer still exists. This is more likely to be new and is highly relevant for the molecular lock argument later in the manuscript. Apologies if this was the original intent, but the text as-is can give an improper impression.

> We believe the uniqueness statement described in the corresponding sentence is correct, though it can be refined for further clarity. In the referenced carboxysome and Pdu compartment systems, cargo recognizes lumenal surfaces formed by multiple shell protein junctions. In contrast, AaRS targets the pentamer-pentamer assembly interface. The CLS-mediated anchoring physically occupies this interface during cage construction, blocking final pentamer insertion until cage completion. This recognition mode is fundamentally distinct from cargo binding at lumenal junction on assembling shell surfaces, observed for other compartment systems. Nevertheless, to clarify this point further, we have revised the text (**page 8, line 146**) to emphasize that RS targets the “actively assembling” interface, as the reviewer suggests. We have also revised the Introduction (**page 4, lines 63-67**) to acknowledge that cargo recognition at lumenal regions where shell proteins converge has been commonly observed in related compartments including encapsulins, carboxysomes, and Pdu compartments.

• Page 20, lines 399-405: Bacterial microcompartment shells can autonomously assemble (PMID: 28642439 is the first of many polyhedral examples, “other” morphologies also exist PMID: 36932732, PMID: 36786617, PMID: 41268919). The role of condensation mechanisms in facilitating carboxysome and metabolosome formation (PMID: 32123388, PMID: 40790117, PMID: 35614058, plus the current citation #13) also inherently implies cooperative assembly with guest enzymes. Further, shell components can help to template bacterial microcompartment formation by providing seed sites (so called “shell-first”) for nucleation cascades (PMID: 35614058, PMID: 38425792 reviews both shell-first and other pathways). Please update this passage with these prior observations in mind.

> We agree with the reviewer's comment. The sentence "In contrast, LS possesses an inherent capacity for autonomous assembly into regular dodecahedra" suggests that other bacterial compartment shell proteins cannot autonomously assemble, which is not true. As the reviewer listed, some BCM shells proteins are known to form polyhedral assemblies. We apologize for the misleading statement.

Our intention was to emphasize that many bacterial compartments require guest-templating to achieve assemblies with naturally required geometry. This applies not only to systems employing condensation mechanisms but also to shell-first mechanisms that use guest networks as assembly scaffolds. In these systems where cargo enzymes direct shell construction, empty cage formation is unlikely to occur, and lumenal recognition, particularly at junction regions, is likely preferable for their highly cooperative assembly mechanisms. Meanwhile, LS represents a different case. Without guest templates, LS shell proteins spontaneously form native-like, homogeneous assembly with desired geometry. Notably, we have not observed any disassembled cage fragments when AaLS is produced recombinantly, indicating the assembly rapidly proceeds to completion. This self-standing, rapid assembly without guest templating raises the potential issue of empty or insufficiently loaded cage formation, which are functionally unfavorable. The delayed cage completion mechanisms described in this study provide a solution to prevent underloaded compartment formation.

We have revised the paragraph entirely (**page 23, lines 463-477**) to clarify that we do not claim BMC shells cannot assemble autonomously, but rather to highlight the distinction between guest-templating assembly mechanisms and LS self-assembly. While we appreciate these preceding studies the reviewer suggested, the condensation and the shell-first mechanism are not distinguished in this manuscript to focus on the differences from LS system. We also added rapid, self-standing assembly of LS which potentially holds a danger of empty cage formation. This more plausibly explains why LS employed the delayed completion mechanism.

Smaller Concerns:

• Page 20, lines 406-408: It may be worth comparing the stability of LS cages to findings from encapsulins and bacterial microcompartments, if available. It would help to drive this point home.

> Realizing that stability after assembly is not the major difference to explain the CLS-anchoring mechanism, we removed the stability status from the discussion paragraph. Alternatively, we added rapid, self-standing assembly properties of LS as discussed in the previous comments.

• Double check that "lumenal" is intended as opposed to "luminal". The latter is more common, but I believe both are acceptable.

> We have intentionally used "lumenal" as "luminal" may refer to photon-related contexts.

• Page 7, line 132: I believe the Figure reference should be for Fig. 1h, not 2h.

> Thank you for pointing out the mistake. We have corrected the figure indication accordingly.

• Page 11, line 216: I believe "geometry" should be "geometries".

> Thank you. We have corrected "geometry" to "geometries".

• Out of my own curiosity, when the conformational lock occurs upon final pentamer insertion, are the AaRS proteins "dislodged" from their anchor site? What is their fate within the LS cage?

> This is an excellent question. While we have not definitively proven this, we believe RS is dislodged from the pre-C3 binding sites upon cage closure. We observe substantial cryo-EM density inside fully assembled AaLS cages, but this density is not well-defined structurally, suggesting that the encapsulated RS is no longer rigidly bound at the pre-C3 sites and has become mobile within the lumenal space.

Reviewer #3 (Remarks to the Author):

Koziej et al. have presented a molecular basis for encapsulation of homotrimeric riboflavin synthase (RS) by the dodecahedron lumazine synthase (LS) protein cage in Aquifex aeolicus: i.e., the AaRS/AaLS complex. They disclose several high-quality cryo-EM maps and models of the empty AaLS and AaRS/AaLS complex to support their model for how the cage-localization signal (CLS) peptide of AaRS docks to the lumenal surface at pentamer-pentamer interfaces of the AaLS cage during assembly. They used a CLS-GFP mimic of the natural AaRS cargo to measure cage assembly through native-gel fluorescence and leveraged these data to explain how the fully mature AaRS/AaLS complex prevents self-disassembly through a stabilizing electrostatic interaction. They further co-opted MD and Rosetta calculations to interrogate the importance of residue interactions between CLS and AaLS protomers of the AaRS/AaLS complex. Finally, they employed phylogenetic analysis and sequence alignments to understand how prevalent these mechanisms for RS/LS cage formation are across different species and kingdoms of life. While the authors present high-quality data and associated analyses, they should try to improve the data presentation so it (1) is more intuitive to non-experts and (2) completely satisfies their claims (see specific comments below). In addition, the reviewer has identified some concerns (detailed below) that should be addressed before this manuscript is published. Most importantly, the proposed "gate and lock" mechanism, although interesting, appears to be not fully substantiated by the data. As suggested below, the authors may consider alternative approaches to testing this idea (e.g., cargo exchange experiments or more convincing electrophoretic analysis). The extrapolations made to other RS/LS systems in nature was also not fully supported by the data and should be addressed (see below).

1. Pg. 3, line 50: Can the authors be more specific about where the RS/LS complex occurs in nature, at least at the kingdom level?

> We have clarified that RS/LS complexes are found in bacteria (**page 3, line 50**). The complex was originally characterized in *Bacillus subtilis* (Ref. 3), and *Aquifex aeolicus* RS/LS complex formation has been confirmed in previous studies (Ref. 21). We intentionally keep the Introduction focused on the RS/LS system as a model rather than emphasizing specific organismal distribution. The phylogenetic distribution and sequence determinants of RS/LS complex formation across bacterial lineages are examined in detail in the Results section (**pages 17-20, Fig. 4**).

2. Pg. 4, line 71: Please include RS:LS in reference to 3:60 stoichiometry for readers.

> We have clarified the stoichiometry notation (**page 4, line 73**) to explicitly state "3:60 (RS:LS)".

3. Fig. 1: Please draw the phosphoryl groups in their appropriate charged states at near-neutral pH.

> We have revised **Fig. 1** to depict the phosphoryl groups of compounds (2) and (4) with explicit negative charges (-OPO_3^{2-}), reflecting their predominant ionization state at physiological pH (6.8-7.5).

4. Extended Data Fig. 1: It is difficult to understand how the text and images match in this figure. Can the authors revise this figure, perhaps with addition of arrows and numerical steps, to make it easier to interpret?

> We agree with the reviewer's comments. The scheme was difficult to follow. We have added arrows and reorganized the figure panels for clarification of the cryoEM data analysis flow (**Supplementary Fig. 1**).

5. Pg. 7, line 132: There appears to be an incorrect reference to Fig. 2h.

> Thank you for pointing out the mistake. We have corrected the figure reference from Fig. 2h to Fig. 1h accordingly.

6. Pg. 7, lines 134-135: It would be useful to include a close-up model of the red helix interacting with the clearly demarcated "pre-C3" area. Otherwise, it is hard to gather what it meant by this with the very small panels in Fig. 1h-j.

> Thank you for the suggestion. We have added a panel for amplified view to clarify what the pre-C3 site refers to (**Fig. 1i,l**). We also labeled the C3 symmetry region of AaLS dodecahedral structure in panel b.

7. Pg. 7, lines 138-139: Why is asymmetry anticipated when there are many C3 axes of symmetry in the dodecahedron? Could one not also imagine three, symmetry-related binding sites for the CLS peptides?

> We thank the reviewer for this clarification. We have revised the text to clarify what we meant by "asymmetric." While the five potential binding sites at each pentagonal interface are indeed symmetry-related (arranged with 5-fold rotational symmetry), we anticipated that their occupancy by the three CLS peptides would be non-uniform—that is, not all five symmetry-related sites would be equivalently occupied. The revised text now explicitly states this 3:5 mismatch and avoids the potentially confusing term "asymmetric" (**page 8, line 148-149**).

8. Pg. 7, lines 138-142: Again, it is quite difficult to make sense of this from the very small figure panels. Can the authors consider breaking Fig. 1 into two separate main text figures or making surrounding panels (e.g, panel k) smaller to increase the size of the cryo-EM maps? Currently, Fig. 1k is larger than necessary, and it is also lacking labels. Ideally, the authors would show models of RS/LS fit inside the maps to support these statements, at least as an SI figure.

> We thank to the reviewer's comments. We have made the AaRS model smaller (**Fig. 1m**) and added a new panel to clarify the pre-C3 sites (**Fig. 1i**). We also labeled the structure to make the main text description easier to follow. The model fitting within cryoEM density is now shown in **Fig. 1l** and **Supplementary Fig. 5**.

9. Pg. 8, lines 143-146: Fundamentally, the RS heterotrimer must be encapsulated by the pre-formed cage before the pore size becomes too small for entry. Can the authors comment on the oligomeric states of LS

that would be compatible with RS entry based on these simple geometric criteria? It would be helpful to delineate what can be logically deduced and what the authors present as new knowledge regarding the timing of events during cage assembly.

> We thank the reviewer for this important question about geometric constraints on cargo loading. Size comparison of the pentagonal pore with the AaRS trimer reveals that the guest cargo requires at least three pentamers missing from the complete cage to pass through the opening. This geometric constraint indicates that the majority of AaRS trimer loading must occur during early to intermediate assembly stages, when the cage structure remains sufficiently open. Our cryo-EM structures of AaRS bound to both 10- and 11-pentamer assemblies provide direct evidence that this represents the predominant loading pathway. The identical binding mode in both assemblies suggests AaRS recruitment occurs before these late-stage intermediates form, with the captured particles representing relatively abundant species during cage assembly.

We have added this geometric analysis to the manuscript (**page 8, lines 157-163, Supplementary Fig. 4**) to clarify what can be logically deduced from size considerations versus what our structural data demonstrate about the timing of cargo loading.

10. Pg. 8, lines 150-154: Can reference to a model be made for this structural description? It is difficult to understand what is meant from these words alone.

> We have added labels to the relevant figure panels (**Fig. 1m**) and clearer references in the main text (**page 9, lines 167-169**) to help readers visualize the structural features being described.

11. Pg. 8, lines 161-163: A reference to the relevant model and map should be made here. Ideally, the model fit inside the map should be shown.

> We have added a reference to the relevant model and map in the main text (**page 9, lines 178-180**). We now show the model fit inside the map in **Fig. 1l** and **Supplementary Fig. 5** to support these structural descriptions.

12. Fig. 2: Can the authors please label all the residues shown in this figure (e.g., panel b)?

> We have added labels for all residues shown in **Figs 2 and 3** to improve clarity.

13. Fig. 2: Why is GFP not stained by readyblue?

> Free GFP-CLS was present at quantities below the detection limit of ReadyBlue staining under these loading conditions, and was therefore visualized only by fluorescence detection. We have clarified this in the **Fig. 2 legend (line 207-209)**.

14. Pg. 11, line 215: Can the authors express "<±30%" in a more sensible way?

> We have revised the text (**page 12, line 235-239**) to report the specific binding efficiencies for each mutation rather than using the ambiguous "<±30%" notation.

15. Pg. 12, line 247: Can the authors explain what is meant by "the incoming partner" and "units" in these two sentences?

> We have clarified the text (**page 13, line 275**) by replacing "units" with "copies" to make clear we are referring to multiple instances of the 129-133 loop. The "incoming final pentamer" refers to the last pentamer that completes the dodecahedral cage, which we believe is clear from the context of cage assembly.

16. Pgs. 12-13, lines 250-255: Is there any evidence for anti-cooperative cage assembly, and if so, can the authors please cite it?

> The accumulation of 10- and 11-pentamer intermediates in our cryo-EM data provides evidence for anti-cooperative assembly, indicating that late-stage assembly steps are kinetically slowed. In typical cooperative protein cage assembly, such late-stage intermediates are rapidly consumed and exist only in trace concentrations (**Refs. 16,32,33**, now cited). The substantial populations of these near-complete intermediates directly demonstrate anti-cooperative behavior in the AaLS system.

17. Pg. 14, lines 288-291 (part 1): The authors should explain why GFP does not stain with readyblue to support the idea that "unbound GFP-CLS" is truly present. In other words, shouldn't there be a loading control with only GFP-CLS to know where it migrates? Presumably this is why GFP was loaded, but why then is the protein not visible with Coomassie blue (readyblue) stain?

> We have included a GFP loading control to demonstrate where free GFP migrates. The small CLS tag (12 amino acids, net charge +1) added to GFP in the experimental samples is unlikely to significantly alter the migration position compared to free GFP. Free GFP is present at quantities below the detection limit of ReadyBlue staining under these loading conditions, which is why it is visualized only by fluorescence detection. The fluorescence signal in the sample lanes confirms the presence of unbound GFP-CLS. We have clarified this in the figure legend (**page 11, line 207-209**).

18. Pg. 14, lines 288-291 (part 2): Have the authors considered analyzing the relative rates of cargo exchange by adding purified RS to LS-encapsulated GFP-CLS (or conversely, by adding purified GFP-CLS to LS-encapsulated RS)? If the proposed gate and lock mechanism is truly mediated by E32, then the authors might expect faster (or higher yield) exchange for the E32A mutant relative to WT.

> We appreciate this thoughtful suggestion. If technically feasible, such experiments could provide valuable insights into cargo-cage dynamics. However, several technical and mechanistic considerations currently prevent implementation of the proposed cargo exchange experiments.

We have previously attempted to load CLS cargo into pre-formed, isolated AaLS cages and found that such loading is undetectable without conditional destabilization of the cage structure (**ref. 22**). This barrier to external cargo entry limits our ability to test whether E32A facilitates cargo uptake. Moreover,

the E32-mediated lock appears to prevent cargo escape from inside rather than facilitate external cargo entry, suggesting these processes operate through different mechanisms. Even if E32A compromises cargo retention, this would not necessarily enable the external cargo entry required for exchange experiments. Furthermore, GFP-CLS presents only a single CLS peptide versus the three presented by RS trimers, likely providing insufficient binding affinity at pre-C3 sites. We attempted to solve the structure of GFP-CLS at pre-C3 sites, but could not detect the corresponding density, suggesting the cargo was washed out from the incomplete AaLS cages during purification. These differences in binding mode would complicate interpretation of any exchange experiments using GFP-CLS to report on RS-specific lock dynamics.

Overcoming these limitations would require developing new methods for controlled cargo loading and validation with native RS cargo, representing substantial method development beyond the current study's scope. Given the inability to load cargo into stable cages, mechanistic differences between cargo entry and retention, and limitations of available cargo surrogates, the proposed experiments would be difficult to implement and interpret conclusively at this time. Notably, E32A was originally designed to test E32's role in CLS recognition but showed minimal effect on binding. The observed increased GFP-CLS leakage was an unexpected finding that led to our interpretation of the E32-R29 salt bridge as a retention lock rather than a recognition element.

19. Fig. 4, panel b: Can the details of BsRS/BsLS be more closely examined (at least in the SI) to show how the CLS peptide engages with the LS surface at a pre-C3 (or related) site? This would help to understand how general the binding mode of AaRS/AaLS is.

> We have added a detailed view of the CLS-LS interaction, predicted for 5 different variants including BsRS/BsLS, to **Supplementary Fig. 10**. The predicted binding mode shows conservation of key features observed in the AaRS/AaLS system, including CLS engagement at pre-C3 interfaces through hydrophobic interaction.

20. Fig. 4, line 321: What are the criteria for organisms to be predicted to form RS/LS complexes?

> We have briefly mentioned the prediction criteria in the **Fig. 4c legend**. Organisms were classified as complex-forming if at least one AlphaFold model (out of 15 generated per organism) showed CLS binding to the pentamer-pentamer interface at the pre-C3 region. Detailed prediction methodology is described in the Materials and Methods section.

21. Fig. 4, line 326: Can the authors explain why SoLS is not higher in RS/LS band intensity ratio? There is a visible band above SoLS that runs consistent with RS in the neighboring wells. By eye, it does appear to be higher than plotted below by densitometry, but this may not be so.

> We agree that visual inspection of the SoRS/SoLS sample suggests a higher RS/LS ratio than other negative samples. This is exactly why we performed densitometry analysis, showing that the RS band intensity is substantially lower than the clear complex-forming samples. While we cannot definitively exclude the possibility that SoRS/SoLS forms complexes at low levels, we classified it as non-complex-forming based on the densitometry threshold applied consistently across all samples.

22. Pg. 16, line 336: Like the above comment, it is not clear how these predictions are made. Can the authors comment?

> The detailed classification criteria are described in the Materials and Methods section. As noted in our response to Comment 20, we have now also added a brief summary of the prediction criteria to **Fig. 4 legend** for clarity.

23. Pg. 17, lines 340: "Complete concordance" does not sit well with the reader when the AF models (Ext. Data Fig. 7) only show two out of the six RS/LS pairs tested in Fig. 4e. Furthermore, the graphics in Ext. Data Fig. 7 could be better labeled, and close-up views of the modeled CLS-LS interactions as in Fig. 2a-c would be helpful to support the authors' conclusions.

> We have added close-up views of the predicted CLS-LS interactions with labeling in **Supplementary Fig. 10**. All AlphaFold prediction results for the six tested RS/LS pairs are provided in **Supplementary Figs. 9, 11, and 12**. In Supplementary Fig. 10, we show representative examples to illustrate the range of predicted binding modes without redundancy, as the complete AlphaFold dataset will be accessible to readers in a public depository, Rodbuk, DOI: 10.57903/UJ/CVWGR3 (see Data availability) upon publication.
